# Batten disease protein CLN8 enables a non-canonical phospholipid synthesis pathway

**DOI:** 10.1038/s41556-026-02059-8

**Published:** 2026-09-15

**Authors:** Johannes Breithofer, Nermeen Fawzy, Clara Zitta, Martin Tischitz, Dominik Bulfon, Clemens Hofmann, Lennart Hartig, Carina Wagner, Gernot F. Grabner, Anita Pirchheim, Achim Lass, Ulrike Taschler, Keira Turner, Kasparas Petkevicius, Ulrich Stelzl, Dagmar Kratky, Rolf Breinbauer, Robert Zimmermann

**Affiliations:** 1 Institute of Molecular Biosciences, University of Graz, Graz, Austria; 2 Cell Death and Metabolism Unit, Danish Cancer Institute, Copenhagen, Denmark; 3 Institute of Organic Chemistry, Graz University of Technology, Graz, Austria; 4 Gottfried Schatz Research Center, Molecular Biology and Biochemistry, Medical University of Graz, Graz, Austria; 5 BioTechMed-Graz, Graz, Austria; 6 Field of Excellence BioHealth, University of Graz, Graz, Austria; 7 MRC Mitochondrial Biology Unit, University of Cambridge, Cambridge Biomedical Campus, Cambridge, UK; 8 Institute of Pharmaceutical Sciences, Pharmaceutical Chemistry, University of Graz, Graz, Austria

**Keywords:** Phospholipids, Lysosomes, Mechanisms of disease

## Abstract

According to text book knowledge, de novo glycerophospholipid (GPL) synthesis begins with the acylation of glycerol-3-phosphate to form phosphatidic acid, the precursor of all other GPLs. Here we describe an alternative GPL synthesis pathway that starts with the acyl-CoA-dependent acylation of glycerophosphoglycerol, resulting in the formation of lysophosphatidylglycerol. The acyltransferase reaction is catalysed by the Batten disease-associated protein ceroid lipofuscinosis neuronal 8 (CLN8). Tracer studies revealed that CLN8-derived lysophosphatidylglycerol is selectively converted into bis(monoacylglycero)phosphate (BMP), a GPL essential for lysosomal lipid homeostasis but not into phosphatidylglycerol or cardiolipin. *CLN8*-knockout cells and mice cannot utilize glycerophosphoglycerol for BMP synthesis, resulting in BMP deficiency and excess accumulation of phospholipids in lysosomes. The lipid synthesis pathway described herein is relevant for understanding lysosomal lipid metabolism and the pathogenesis of neurodegenerative diseases. BMP deficiency may contribute to or even underlie lysosomal cargo accumulation in certain forms of Batten disease and other lysosomal storage disorders.

## Abstract (web summary)

Two studies show that the ER-associated protein CLN8 catalyses the acyl CoA-dependent acylation of glycerophosphoglycerol, producing lysophosphatidylglycerol, which is then converted into BMP. These results provide insight into CLN8 Batten disease.

## Main

Membranes of different organelles are characterized by defined lipid and protein compositions, which determine their function. The lipid composition affects membrane fluidity and curvature and is essential for folding and activity of membrane-associated proteins. Intralumenal membranes of late endosomes and lysosomes contain bis(monoacylglycero)phosphate (BMP) as lipidomic signature^1^. BMP plays a central role in lysosomal lipid homeostasis. It is highly resistant to lysosomal hydrolases^2^, promotes the formation of intralumenal membranes^3^ and acts as a cofactor of lysosomal lipid hydrolases and transport proteins that mediate the breakdown and export of lipids^4^.

BMP is a structural isomer of phosphatidylglycerol (PG), indicating that PG acts as precursor of BMP. However, these lipids differ in their stereo- and acyl chain configuration. PG exhibits *sn-*3-glycerophosphate-*sn-*1-glycerol (*R*,*S*) backbone configuration and is acylated exclusively at the *R*-bound glycerol. Conversely, BMP has an *S*,*S*-backbone configuration and contains a single fatty acid (FA) group at each glycerol residue^5–7^, implicating that the conversion of PG into BMP requires both stereoconversion and positional rearrangement of FAs.

Previous studies suggest that PG is converted into BMP within lysosomes. This process requires the activity of lysosomal hydrolases degrading PG to lysophosphatidylglycerol (LPG)^8^, followed by the conversion of LPG into BMP via transacylation and transphosphatidylation reactions^9,10^. Here we show that the synthesis of BMP begins at the endoplasmic reticulum (ER) via an unusual reaction that is independent of established de novo GPL synthesis pathways starting with the acylation of glycerol-3-phosphate^11,12^. The ER-associated protein ceroid lipofuscinosis neuronal 8 (CLN8) catalyses the acyl-CoA-dependent acylation of glycerophosphoglycerol (GPG), producing LPG, which is selectively converted into BMP. *CLN8*-deficient cells and mice lack GPG acyltransferase activity and BMP. Mutations in the *CLN8* gene are linked to the neurodegenerative disorder Batten disease^13–16^, characterized by the accumulation of autofluorescent lipopigments (ceroid lipofuscin) in lysosomes of neurons and other cells. Our observations suggest that the lack of BMP is causal for the lysosomal storage disorder in *CLN8*-deficient cells.

## Results

### GPG is used for LPG and BMP synthesis

**Fig. 1:**
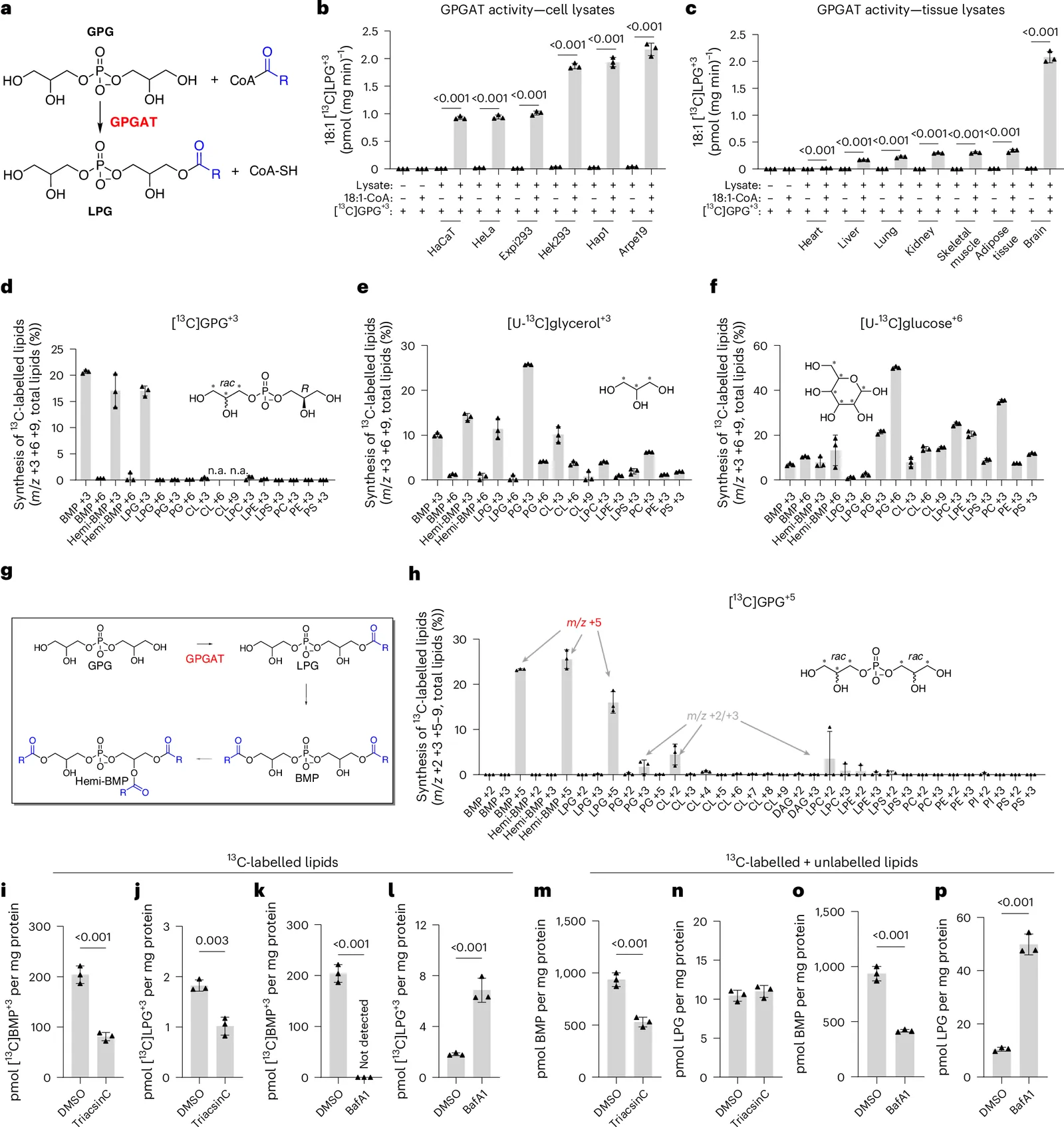
Mammalian cells utilize GPG for phospholipid synthesis. **a**, A schematic of the GPGAT reaction. **b**,**c**, GPGAT activity in lysates (1,000*g* supernatant) of human cell lines (**b**) and mouse tissues (**c**) using 100 µM [^13^C]GPG^+3^ and oleoyl-CoA as substrate (pH 7). [^13^C]LPG^+3^ formation was analysed by LC–MS (*n* = 3). **d–f**, Incorporation of [^13^C]GPG^+3^ (500 µM) (**d**), [U-^13^C]glycerol^+3^ (500 µM) (**e**) and [U-^13^C]glucose^+6^ (25 mM) (**f**) into phospholipids of Hek293 cells after 16 h incubation (the asterisk indicates ^13^C-atoms). In **f**, glucose of the medium was replaced by [U-^13^C]glucose^+6^. The results are presented as a percentage of labelled lipids relative to total lipids for each lipid class examined (*n* = 3, representative of two independent experiments). **g**, A schematic of GPG-dependent BMP and hemi-BMP synthesis. **h**, Incorporation of [^13^C]GPG^+5^ (250 µM) into lipids of Hek293 cells. Experiments were performed as described in **d**–**f** (*n* = 3). **i–l**, Effect of triacsin C (10 µM) (**i** and **j**) and bafilomycin A1 (BafA1, 0.1 µM) (**k** and **l**) on the incorporation of [^13^C]GPG^+3^ (500 µM) into LPG (**j** and **l**) and BMP (**i** and **k**). Incubations were performed for 18 h in the presence of inhibitors or DMSO as vehicle control (*n* = 3). **m–p**, Effect of triacsin C (**m** and **n**) and BafA1 (**o** and **p**) on endogenous BMP (**m** and **o**) and LPG (**n** and **p**) levels of Hek293 cells (labelled plus unlabelled). Experiments were performed as described in **i**–**l** (*n* = 3). Lipid species were analysed with LC–MS and are presented as sum of analysed subspecies. In **b** and **c**, *n* represents independent assay replicates derived from a cell or tissue lysate; in **d**–**f**,**h**–**p**, *n* represents independent well replicates. Data are presented as mean ± s.d. Statistical comparisons in **b**,**c**,**i**–**p** were performed with unpaired two-tailed Student’s *t*-test. *P* values are indicated. Source data

The deacylation of GPLs by (lyso)phospholipases A1/A2 generates glycerophosphodiesters (GPDs), including GPG, which constitutes the backbone of PG and BMP. GPDs are currently thought to be degraded by glycerophosphodiesterases, thereby providing building blocks for de novo lipid synthesis and signalling molecules^17^. To investigate whether mammalian cells can reuse GPG for GPL synthesis, we incubated cell and tissue lysates with GPG containing ^13^C-labelled glycerol (*sn*-[U-^13^C]glycero-1(3)-phospho-*sn*-3-glycerol (*rac*,*R*); *m*/*z* +3; [^13^C]GPG^+3^) in the presence and absence of oleoyl-CoA (Fig. 1a). These experiments revealed that [^13^C]GPG^+3^ is acylated in an acyl-CoA-dependent manner in the presence of lysates of human cell lines and mouse tissues (Fig. 1b,c). Among the tested tissues, the formation of ^13^C-labelled LPG ([^13^C]LPG^+3^) was highest in brain lysates. Thus, mammalian cells and tissues contain acyl-CoA:GPG acyltransferase (GPGAT) activity.

**Extended Data Fig. 1:**
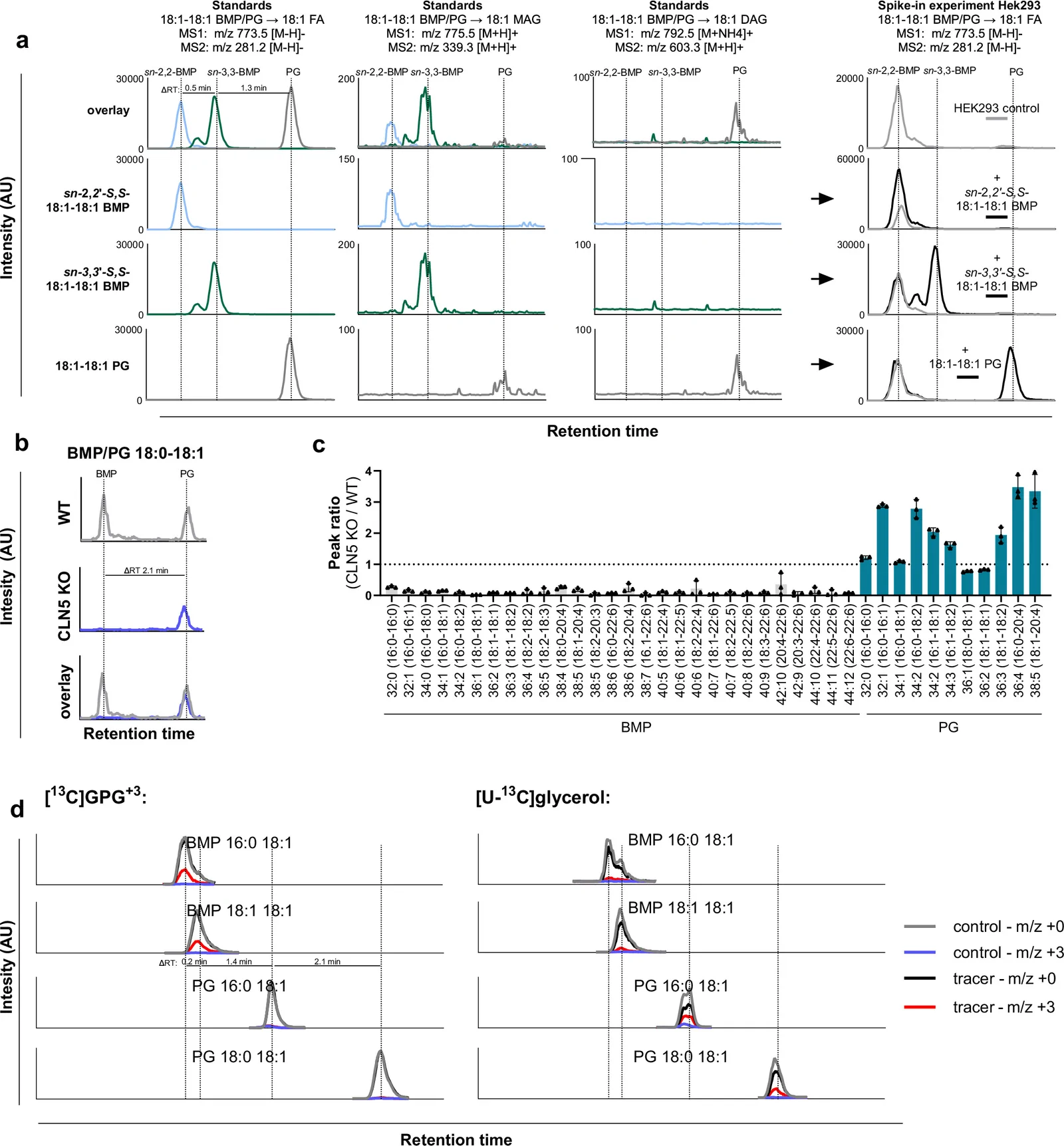
Validation of the LC-MS based method to quantify BMP and PG isomers. **a**, Representative chromatograms of *sn*-3,3′-*S,S*-18:1-18:1 BMP, 18:1-18:1 PG (857135, 840475, both Avanti Polar lipids), and *sn*-2,2′-*S,S*-18:1-18:1 BMP (L-B181, Echelon Biosciences) standards demonstrate baseline separation of the analytes under the applied chromatographic conditions. MS2 analysis of BMP and PG in positive-ion mode enables the detection of a characteristic monoacylglycerol (MAG) fragment for BMP and a diacylglycerol (DAG) fragment for PG. In our analytical setup, BMP and PG are detected with higher sensitivity in negative-ion mode through monitoring of acyl chain (FA) fragmentation products. Spike-in experiments further verified the identity of the endogenous 18:1-18:1 BMP and PG peaks detected in lipid extracts of Hek293 cells. All corresponding MRM transitions are shown as inserts. **b,c**, To validate the retention times of additional BMP and PG species, we applied the method described by Dong, W. et al.^50^. Peak intensities and retention times from BMP-deficient *CLN5*-KO Hek293 cells were compared with those from WT cells (representatively shown for BMP 18:0-18:1, **b**) and used as an additional criterion to distinguish BMP from PG peaks. For all BMP, but not PG species, the *CLN5*-KO versus WT peak ratios were well below 1 (**c**), confirming peak identity (*n* = 3 independent well replicates). The confirmation of gene deletion in *CLN5*-KO cells is provided in Bulfon, D. et al.^18^. **d**, Representative chromatograms of the two most abundant BMP and PG species confirm [^13^C]GPG^+3^ or [U-^13^C]glycerol tracer incorporation into BMP and PG species. Source data are available. Source data

To track the distribution of GPG within phospholipids, we incubated Hek293 cells with [^13^C]GPG^+3^. Mass spectrometry revealed that GPG is preferentially incorporated into LPG, hemi-BMP and BMP. Hemi-BMP, also known as acyl-PG, is considered as intermediate in the synthesis of BMP^18^. Of the analysed BMP, LPG and hemi-BMP species, 21%, 17% and 17%, showed a shift in the mass-to-charge ratio (*m*/*z*) of +3, respectively. (Fig. 1d). In comparison, polyglycerophospholipids such as PG and cardiolipin (CL) and other GPL classes, were poorly labelled. (Fig. 1d). BMP and PG isomers were baseline separated by the applied liquid chromatography–mass spectrometry (LC–MS) method. (Extended Data Fig. 1a–c).

GPG degradation by phosphodiesterases generates glycerol-3-phosphate and glycerol, which can be reused for de novo synthesis of PA, the precursor of all GPLs, as well as for the head group synthesis of polyglycerophospholipids. Accordingly, we also monitored LPG, PG, BMP and hemi-BMP with a *m*/*z* shift of +6. However, we detected no species containing more than one labelled glycerol moiety (Fig. 1d). In comparison, [U-^13^C]glycerol^+3^ or [U-^13^C]glucose^+6^, both delivering glycerol-3-phosphate for de novo lipid synthesis, were incorporated into all detected GPL classes (Fig. 1e,f). As expected, polyglycerophospholipids also contained more than one labelled glycerol (*m*/*z* shift of +6 and +9), suggesting that the ^13^C-labelled glycerol was used for PA and head group synthesis. Chromatograms of BMP and PG species confirmed the selective incorporation of [^13^C]GPG^+3^ into BMP and the preferential use of [U-^13^C]glycerol for PG synthesis (Extended Data Fig. 1d). The differences in the GPL labelling patterns between cells incubated with ^13^C-labelled GPG or glycerol/glucose indicate that (1) GPG enters a specific metabolic pathway leading to the formation of LPG, BMP and hemi-BMP (Fig. 1g) and that (2) GPG is incorporated into GPLs without prior degradation, probably via the GPGAT reaction.

To confirm acylation of GPG, we synthesized GPG containing ^13^C-labelled carbon atoms on both glycerol moieties (*sn*-[U-^13^C]glycero-1(3)-phospho-*sn*-1(3)-[1,3-^13^C]-glycerol (*rac*,*rac*); *m*/*z* +5; [^13^C]GPG^+5^) and again monitored its incorporation into cellular lipids. Direct acylation of this tracer results in lipids with a *m*/*z* value of +5, whereas degradation of GPG and reuse of labelled glycerol for lipid synthesis leads to lipid species with *m*/*z* values of +2 or +3. We detected exclusively formation of labelled BMP, hemi-BMP and LPG with *m*/*z* +5 (Fig. 1h), confirming direct acylation of GPG. Minor labelling of other GPL species with *m*/*z* of +2 or +3, suggest that [^13^C]GPG^+5^ is partially degraded and reutilized for PA or headgroup synthesis (Fig. 1h).

To characterize pathways that mediate the incorporation of GPG into phospholipids, we treated Hek293 cells with triacsin C, an inhibitor of acyl-CoA synthetases 1 and 4 (ref. ^19^) and bafilomycin A1 (BafA1), an inhibitor of lysosomal V-ATPase, which leads to alkalization of lysosomes^20^. Triacsin C reduced the incorporation of [^13^C]GPG^+3^ into BMP and LPG by 60 and 40%, respectively (Fig. 1i,j). This indicates that the availability of acyl-CoAs affects both GPG-dependent LPG and BMP synthesis. BafA1 abolished [^13^C]BMP^+3^ synthesis (Fig. 1k), whereas [^13^C]LPG^+3^ levels increased threefold (Fig. 1l), suggesting that LPG synthesis occurs outside of acidic organelles, whereas BMP synthesis requires functional lysosomes. The alterations observed in tracer studies were partially reflected by changes in total cellular lipid content. Triacsin C reduced BMP levels by 40%, while LPG remained unchanged (Fig. 1m,n). In the presence of BafA1, BMP content decreased by 55% and LPG levels increased fivefold (Fig. 1o,p).

### CLN8 exhibits GPGAT activity

**Fig. 2:**
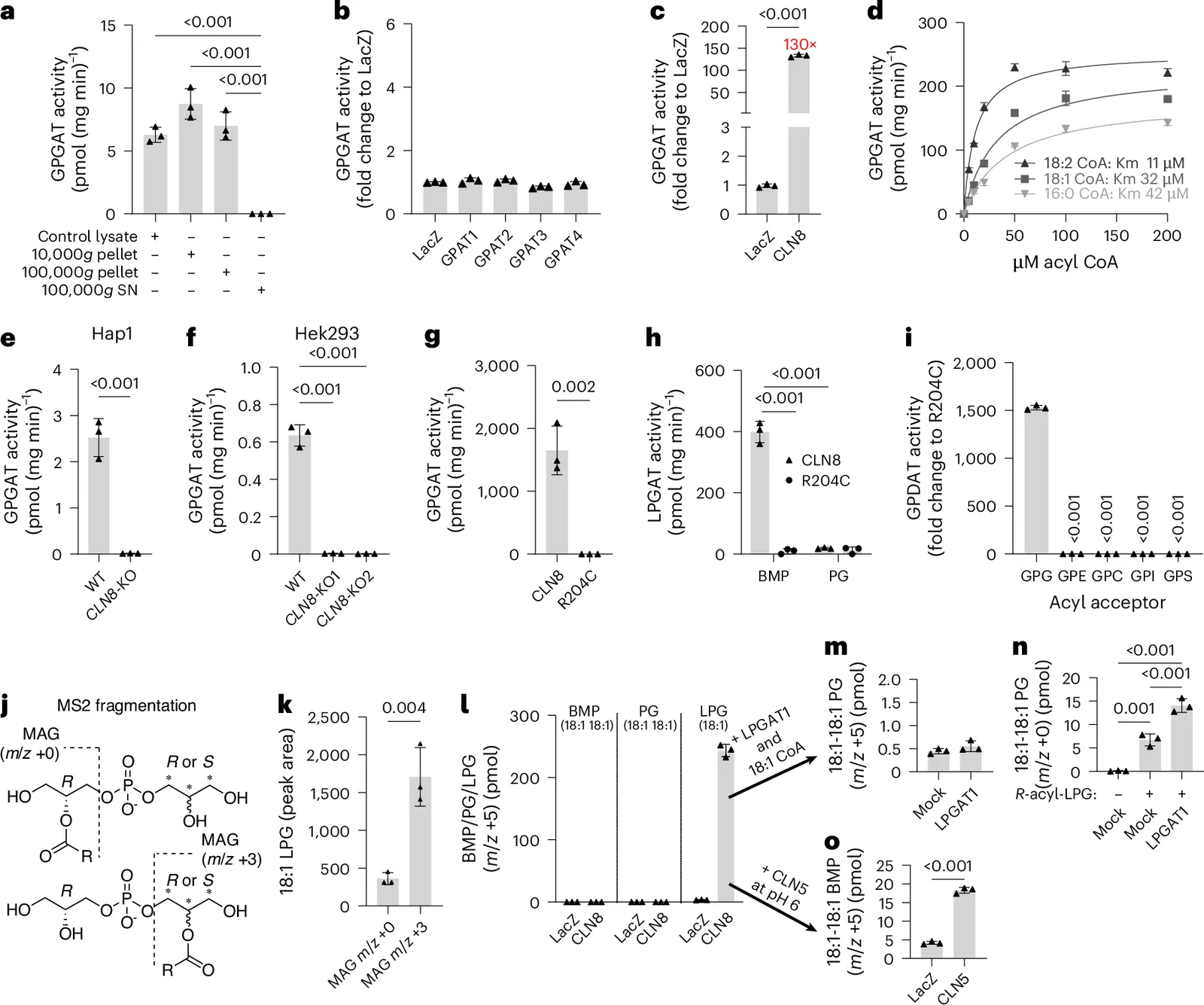
CLN8 exhibits GPGAT activity. **a**, GPGAT activity in subcellular fractions of Expi293 cells (1,000*g* supernatant (SN), 10,000*g* pellet, 100,000*g* pellet, 100,000*g* SN; *n* = 3). **b**, GPGAT activity in Expi293 cell lysates (1,000*g* SN) overexpressing human GPAT1-4 relative to LacZ control (*n* = 3). **c**, GPGAT activity in lysates of Expi293 cells overexpressing human CLN8 relative to LacZ control (*n* = 3, representative of three independent experiments). **d**, GPGAT activity in CLN8-overexpressing Expi293 lysates in the presence of [^13^C]GPG^+3^ as acyl acceptor and increasing concentrations of 16:0-, 18:1- and 18:2-CoAs as acyl donor (*n* = 3). **e**,**f**, GPGAT activity in lysates of WT and *CLN8*-KO Hap1 (**e**) or Hek293 (**f**) cells. Two *CLN8*-KO Hek293 cell clones were analysed (*n* = 3). **g**, GPGAT activity of purified His-tagged CLN8 and its R204C mutant (1 µg each; *n* = 3, representative of two independent experiments). **h**, CLN8-catalysed formation of BMP and PG by purified CLN8 and R204C (1 µg each) using *sn-*1-oleoyl-*R*,*rac* LPG as acyl acceptor (*n* = 3). **i**, Head group specificity of purified CLN8 was determined by the formation of lysophospholipids in the presence of oleoyl-CoA and GPDs (100 µM each); the R204C served as negative control (*n* = 3). **j**, A schematic showing MS2 fragmentations of LPG in *R*,*rac*-[^13^C]-GPG^+3^ backbone configuration. Depending on the acyl-chain position, the monoacylglycerol (MAG) fragments show *m*/*z* values of +0 or +3. **k**, Analysis of MAG fragments (see **j**) of oleoyl-[^13^C]LPG^+3^ generated by purified CLN8. [^13^C]GPG^+3/PG^, derived from headgroup labelled PG, and oleoyl-CoA were used as substrate (*n* = 3). **l**, Formation of [^13^C]BMP/PG/LPG^+5^ by sonicated membranes (100,000*g* pellet) of LacZ and CLN8 expressing Expi293 cells using [^13^C]GPG^+5^ and of oleoyl-CoA as substrate (both 100 µM; *n* = 3). **m**, Conversion of CLN8-derived [^13^C]LPG^+5^ into [^13^C]PG^+5^. After heat inactivation, CLN8-derived [^13^C]LPG^+5^ was further incubated with membranes of cells overexpressing recombinant LPGAT1 and oleoyl-CoA (6 µM). Membranes of mock-transfected cells served as control (*n* = 3). **n**, Conversion of *R*-acyl LPG into PG. The experiment was performed as described in **m** using *R*-acyl-LPG (0.6 µM) and oleoyl-CoA (6 µM) as acyl acceptor and donor, respectively (*n* = 3). **o**, Conversion of CLN8-derived [^13^C]LPG^+5^ into [^13^C]BMP^+5^. After heat inactivation, CLN8-derived [^13^C]LPG^+5^ was further incubated with lysates of cells overexpressing recombinant CLN5 at pH 6.0. LacZ-transfected cell lysates served as control (*n* = 3). Formation of lipids was analysed by LC–MS. In **a**–**i**,**k**–**o**, *n* represents independent assay replicates derived from a cell lysate or purified protein. Data are presented as mean ± s.d. Statistical comparisons in **a**,**f**,**i**,**n** were performed with one-way analysis of variance (ANOVA) followed by Bonferroni posthoc analysis and in **c**,**e**,**g**,**h**,**k**,**o** with unpaired two-tailed Student’s *t*-test. *P* values are indicated. Source data

**Extended Data Fig. 2:**
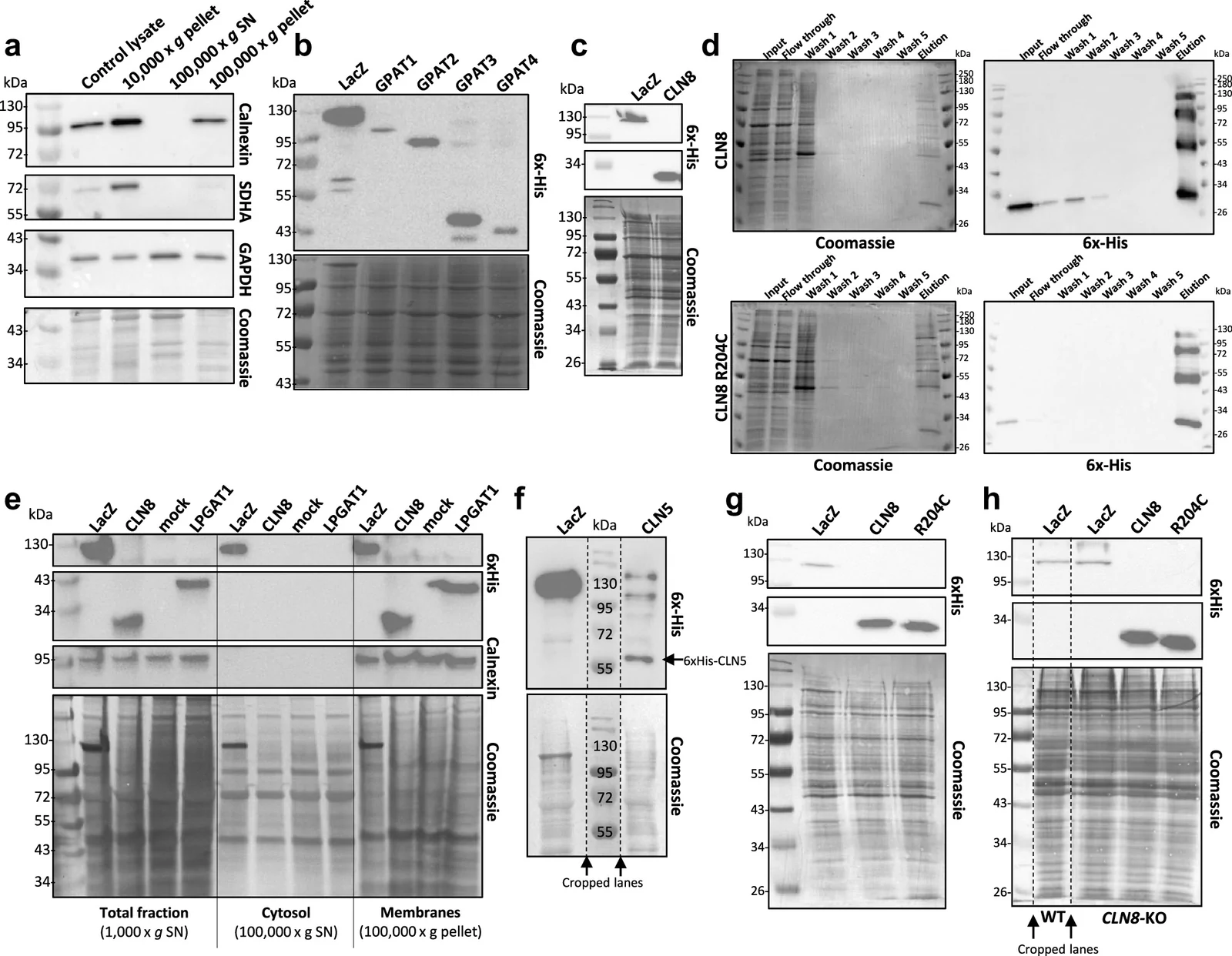
Supporting Western blots. **a**, Western blot of Expi293 control lysate (1,000g supernatant), mitochondrial-enriched fraction (10,000g pellet), cytosolic fraction (100,000g supernatant), and microsomal fraction (100,000g pellet), showing the expression of respective marker proteins succinate dehydrogenase (SDHA), glycerinaldehyde-3-phosphate dehydrogenase (GPADH), and calnexin. **b,c**, Western blot confirming the overexpression of 6xHis-tagged LacZ, GPAT1-4 (**b**), and CLN8 (**c**) in Expi293 cells. **d**, Coomassie stained gel and Western blot of fractions obtained during the 6x-His affinity purification of CLN8 and the CLN8 R204C mutant. In line with published data, multiple bands in the purified fraction (elution) represent multimeric aggregates of CLN8^22^. **e**, Western blot of total fraction (1,000g), cytosolic fraction (100,000g SN), and membrane fraction (100,000g pellet) of Expi293 cells overexpressing 6xHis-tagged LacZ, CLN8, LPGAT1, or mock control (transfection of empty vector). Calnexin was used as a marker for membranes (ER). **f**, Western blot confirming the overexpression of 6xHis-tagged LacZ, or CLN5 in Expi293 cells. **g**, Western blot confirming the overexpression of 6xHis-tagged LacZ, CLN8, and CLN8 R204C in Hek293 cells. **h**, Western blot confirming the overexpression of 6xHis-tagged LacZ, CLN8, and R204C in WT and *CLN8*-KO Hek293 cells. In all blots, Coomassie stain was used as loading control. Dashed lines indicate cropped lanes. Unprocessed gels and blots are available in source data.

To gain insights into the molecular mechanisms mediating GPG acylation, we first separated cell lysates into mitochondria-enriched, microsomal and cytosolic fractions (Extended Data Fig. 2a). GPGAT activity was restricted to membrane-containing fractions, suggesting that this reaction is catalysed by a membrane-bound enzyme (Fig. 2a).

Mammalian cells express four acyl-CoA:glycerol-3-phosphate acyltransferases localized to the outer mitochondrial membrane (GPAT1&2) or the ER (GPAT3&4)^21^. To test whether human GPATs possess GPGAT activity, we expressed these acyltransferases in Expi293 cells (Extended Data Fig. 2b). GPGAT activity in lysates of these cells was unchanged in comparison to the β-galactosidase (LacZ) control (Fig. 2b), implicating that GPG enters a GPAT-independent lipid synthesis pathway.

**Extended Data Fig. 3:**
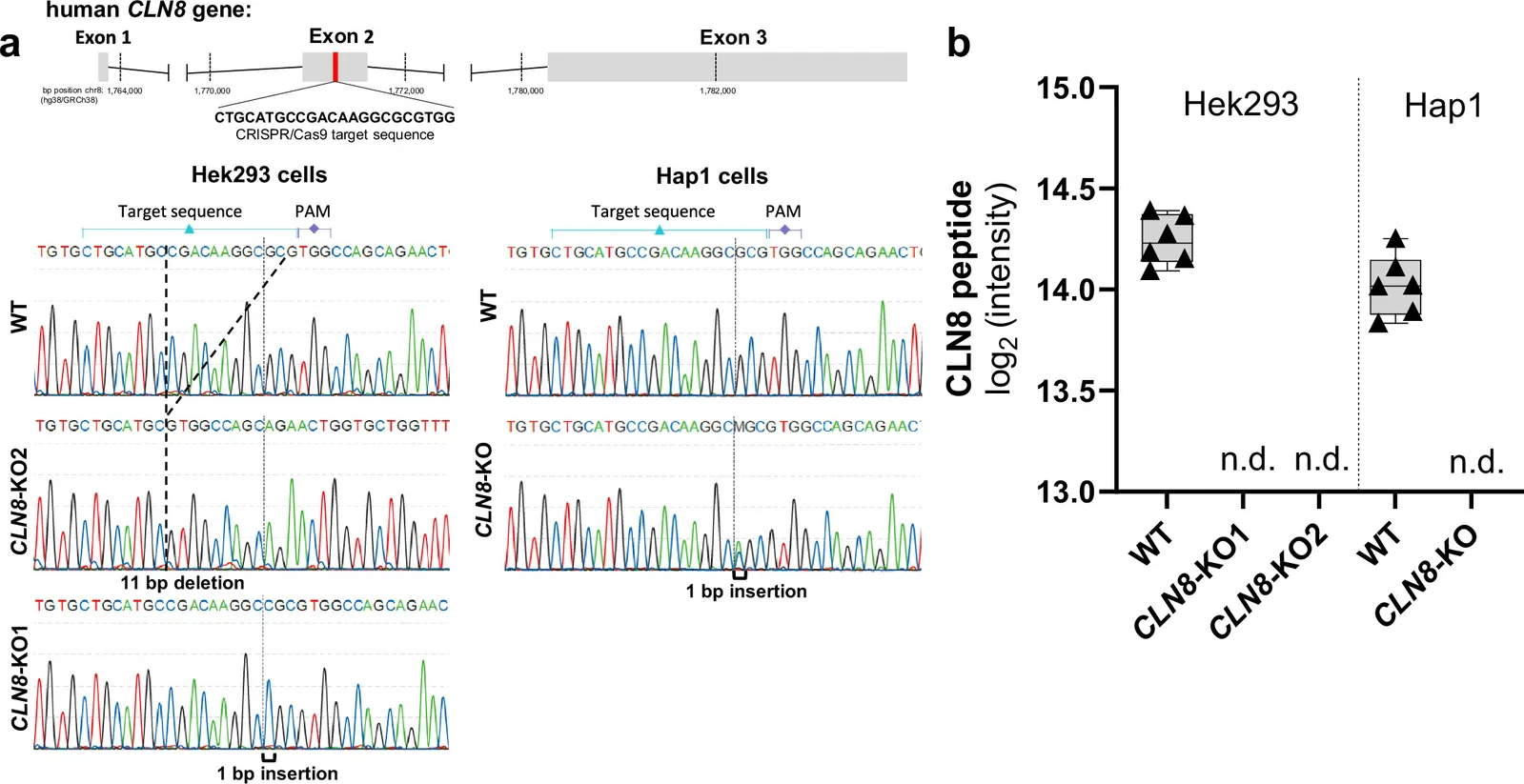
Confirmation of *CLN8*-KO in Hek293 and Hap1 cells. **a**, Sanger sequencing of genomic DNA at the CRISPR-Cas9 targeting locus in the *CLN8* gene (Exon2) of Hek293 and Hap1 WT and *CLN8*-KO clones. CRISPR-Cas9 mediated deletions or insertions are highlighted in the sequencing chromatogram. **b**, Mass Spectrometry based proteome analysis of total Hek293 and Hap1 cell extracts. Relative CLN8 protein levels (log_2_ intensity) of WT and *CLN8*-KO lines are shown (*n* = 6 independent well replicates). Box plots show the median, the 25^th^ and 75^th^ percentiles and the minimum and maximum values (whiskers). No CLN8 peptide was detected in KO clones (n.d.); therefore, no statistical analysis was performed. Source data are available. Source data

Recent observations showed that CLN8, a member of the TRAM-LAG1-CLN8 domain-containing protein family, exhibits acyl-CoA:LPG acyltransferase (LPGAT) activity and affects BMP biosynthesis^22^. Strikingly, overexpression of CLN8 in Expi293 cells (Extended Data Fig. 2c) increased GPGAT activity 130-fold (Fig. 2c). Saturation kinetics with different acyl-CoAs revealed a dose-dependent increase in GPGAT activity and lower *K*_m_ values for linoleoyl- and oleoyl-CoA than for palmitoyl-CoA (Fig. 2d), suggesting that CLN8 preferentially uses polyunsaturated acyl-CoAs as previously described for the LPGAT reaction^22^. CRISPR–Cas9-mediated deletion of *CLN8* in Hap1 and Hek293 cells completely abolished GPGAT activity (Fig. 2e,f). *CLN8* mutations and depletion of CLN8 protein were confirmed by sequencing and proteomics (Extended Data Fig. 3a,b).

GPGAT activity was confirmed with purified CLN8 (Fig. 2g and Extended Data Fig. 2d). The Batten disease-associated R204C variant^15,16^ was inactive (Fig. 2g). Wild-type (WT) CLN8, but not R204C, also catalysed the LPGAT reaction, but at an approximate fourfold lower rate than GPG acylation (Fig. 2g,h). The LPGAT reaction generated exclusively BMP, suggesting that CLN8 mediates head group acylation of LPG (Fig. 2h). Purified CLN8 did not acylate other GPDs than GPG, indicating pronounced head group specificity (Fig. 2i).

**Extended Data Fig. 4:**
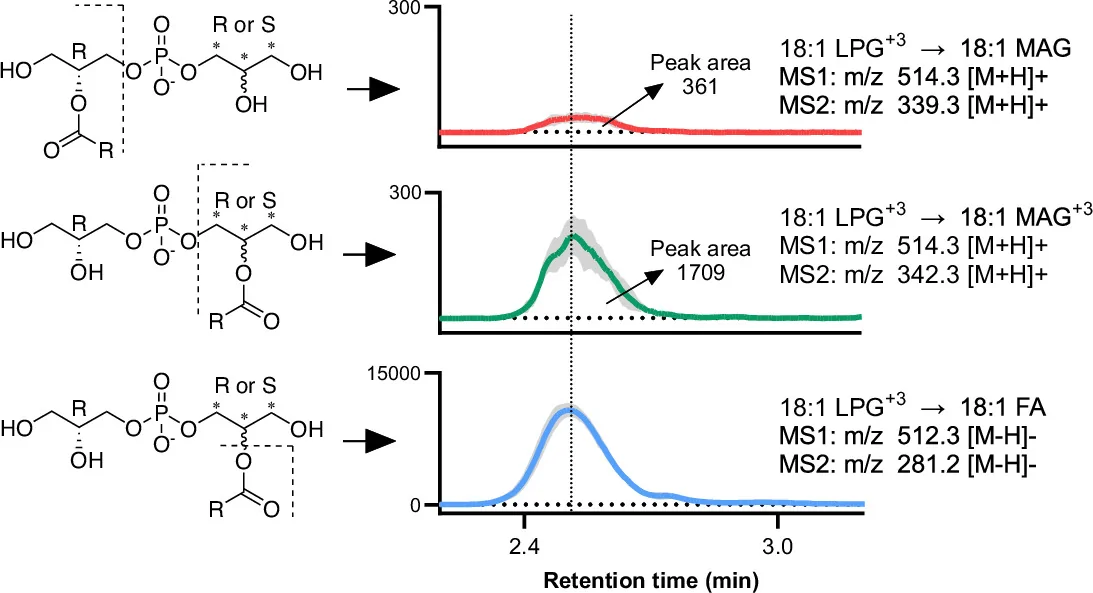
Chromatograms of MS2 fragmentation products of LPG generated by purified CLN8. In the assay, oleoyl-CoA was used as acyl-donor and PG-derived *R,rac*-[^13^C]GPG^+3/PG^ was used as acyl-acceptor. MS2 analysis of LPG in the positive-ion mode enables the detection of a monoacylglycerol (MAG) fragment. Depending on the position of the acyl-chain on the *R,rac*-[^13^C]GPG^+3^ backbone, the MAG fragment exhibits either no mass to charge (m/z) shift (+0; red chromatogram) or a +3 m/z shift (green chromatogram). Simultaneous detection of the 18:1 acyl-chain fragment in the negative-ion mode at the identical retention time further confirms LPG peak identity (blue chromatogram). In our analytical setup, LPG is detected with higher sensitivity in negative-ion mode. All corresponding MRM transitions are shown as inserts. Chromatogram data are shown as mean ± s.d. (*n* = 3 independent assay replicates of purified CLN8). Source data are available. Source data

To investigate the stereospecificity of purified CLN8, we isolated GPG from head group labelled PG and used this probe ([^13^C]GPG^+3/PG^) as acyl acceptor for the acyltransferase reaction. The generated LPG was analysed by MS2 fragmentation analysis. Depending on the acylation of the unlabelled *R-*linked glycerol or the ^13^C-labelled head group, fragmentation of LPG can lead to the formation of either unlabelled or ^13^C-labelled monoacylglycerol species, respectively (Fig. 2j). We observed mostly labelled monoacylglycerol fragments, suggesting that CLN8 preferentially acylates the *S*-linked glycerol moiety (Fig. 2j,k). Representative chromatograms are shown in Extended Data Fig. 4.

Our tracer studies suggested that GPG is not directly incorporated into PG and CL (Fig. 1d,h), which may either be due to spatial separation of LPG and PG synthesis or due to the stereospecific acylation of LPG at the *S*-position. To exclude spatial separation, we monitored lipid synthesis in sonicated membrane preparations of Expi293 cells. CLN8-containing membranes efficiently produced [^13^C]LPG^+5^ in the presence of [^13^C]GPG^+5^ and oleoyl-CoA but no substantial amounts of [^13^C]BMP^+5^ or [^13^C]PG^+5^ (Fig. 2l). Further incubation of CLN8-derived LPG with membranes containing the recombinantly expressed ER-resident LPG acyltransferase 1 (ref. ^23^) (LPGAT1, Extended Data Fig. 2e) did not increase [^13^C]PG^+5^ formation (Fig. 2m). By contrast, supplementation of *R*-acylated LPG (*sn*-1-oleoyl-*R*,*rac* LPG) enabled the formation of PG by membranes of mock-transfected cells, which was elevated twofold in the presence of membranes containing recombinant LPGAT1 (Fig. 2n). Thus, LPGAT1 exhibits stereoselectivity for *R*-acyl-LPG and cannot convert CLN8-derived *S*-acyl-LPG into PG.

To investigate whether the CLN8 product can be used for BMP synthesis, we monitored the conversion of CLN8-derived LPG into BMP at pH 6.0, the pH optimum of the lysosomal BMP synthase CLN5^9^. This enzyme mediates a transacylation reaction in which LPG acts as both FA donor and acceptor, resulting in the formation of BMP and GPG^9^. Under the applied conditions, the formation of [^13^C]BMP^+5^ by cell lysates containing recombinant CLN5 (Extended Data Fig. 2f) was increased fourfold compared with LacZ controls (Fig. 2o). Together, these observations suggest that the stereospecific acylation of GPG by CLN8 at the *S*-linked glycerol moiety prevents PG formation and directs CLN8-derived LPG into BMP synthesis.

### *CLN8*-deficient cells lack BMP

**Fig. 3:**
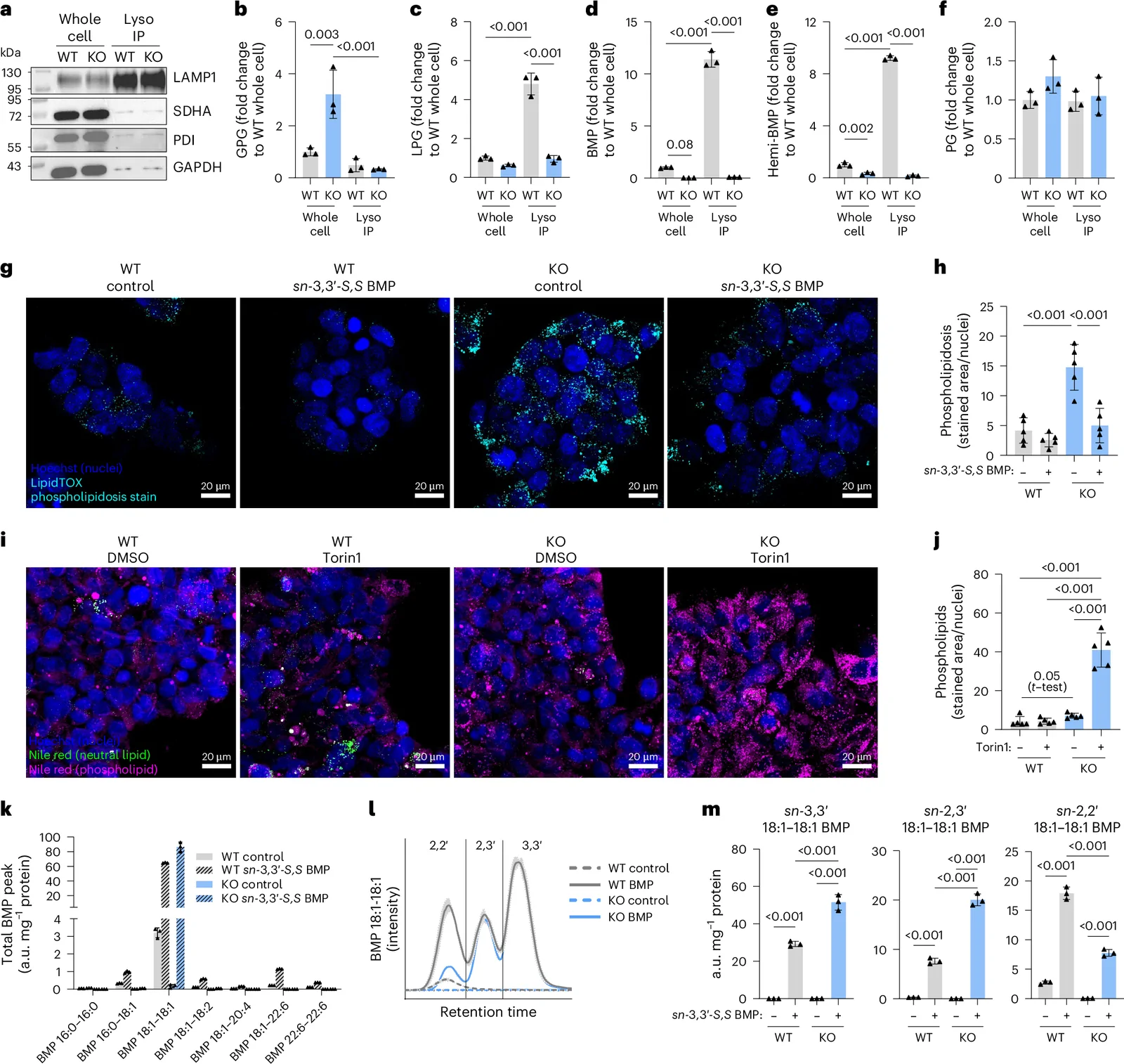
*CLN8*-deletion leads to BMP deficiency and phospholipidosis. **a**, Expression of the lysosomal marker protein LAMP1 in whole-cell fractions and immunopurified^24^ lysosomes of WT and *CLN8*-KO Hek293 cells. Purity of lysosomes was confirmed by western blotting using succinate-dehydrogenase (SDHA), protein disulfide-isomerase (PDI) and glycerinaldehyde-3-phosphate-dehydrogenase (GAPDH) as markers for mitochondria, ER and cytosol, respectively. Equal volumes of each fraction were analysed. **b–f**, GPG (**b**), LPG (**c**), BMP (**e**), hemi-BMP (**f**) and PG (**g**) levels in whole-cell fractions and purified lysosomes of WT and *CLN8*-KO Hek293 cells. Lipids in **c**–**f** are shown as sum of analysed subspecies (*n* = 3; whole cell data are representative of two independent experiments). **g**, Fluorescent imaging of formaldehyde-fixed WT and *CLN8*-KO Hek293 cells stained with the HCS LipidTOX Green phospholipidosis detection reagent (cyan) and Hoechst (blue). Cells were cultured in the absence and presence of 5 µM *sn*-3,3′-18:1–18:1-*S*,*S* BMP (*sn*-3,3′*S*,*S* BMP) for 72 h. **h**, Quantification of the LipidTOX-positive area (*n* = 5 scans per condition; representative of three independent experiments). **i**, Fluorescent imaging of phospholipid deposits (magenta) and neutral lipids (green) in WT and *CLN8*-KO Hek293 cells cultured in the absence and presence of 250 nM Torin1 for 16 h. DMSO was used as vehicle control. Cells were fixed and stained with Nile red and Hoechst before imaging (*n* = 5 scans per condition; data are representative of two independent experiments). **j**, A quantification of the phospholipid area stained by Nile red. Scale bar, 20 µm. **k**, BMP subspecies of WT and *CLN8*-KO Hek293 cells cultured in the absence and presence of 5 µM *sn*-3,3′-*S*,*S* BMP for 72 h (*n* = 3). **l**, Representative chromatogram of 18:1–18:1 BMP from the experiment in **k**, showing the peaks corresponding to 2,2′, 2,3′ and 3,3′ acyl-chain positions (*n* = 3). **m**, A quantification of 2,2′, 2,3′ and 3,3′ 18:1–18:1 BMP peak from the experiment in **k** (*n* =3). In **b**–**f**,**k**–**m**, *n* represents independent well replicates; in **h**,**j**, *n* represents randomly selected microscopy fields within a well. Data are shown as mean ± s.d. Statistical comparisons in all graphs were performed with one-way ANOVA followed by Bonferroni posthoc analysis. *P* values are indicated. Source data

**Extended Data Fig. 5:**
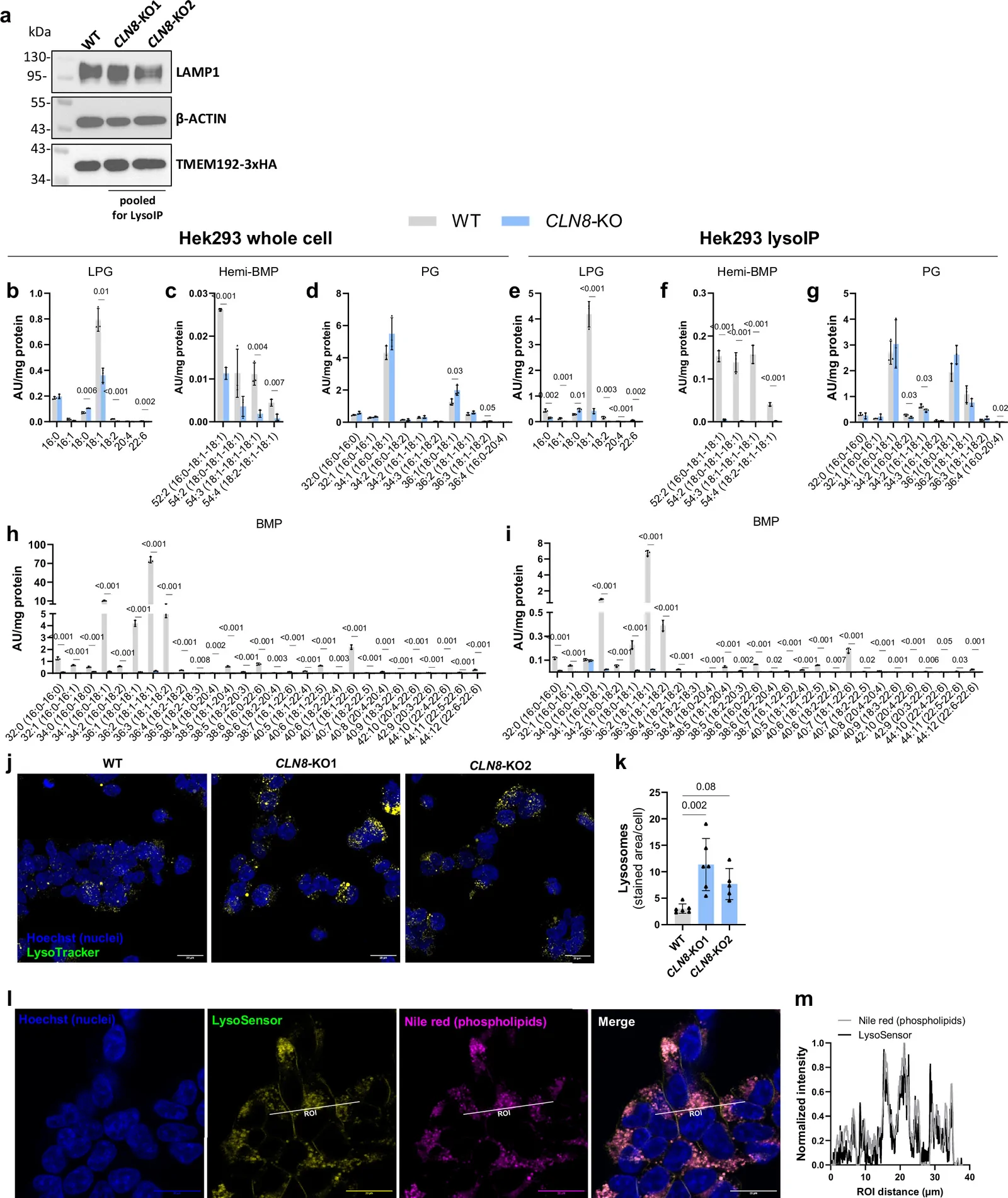
*CLN8*-deficiency disrupts BMP synthesis in Hek293 cells. **a**, Western blot confirming the lentiviral expression of TMEM192-3xHA in Hek293 WT and *CLN8*-KO clones. *CLN8*-KO clones 1 and 2 were pooled for lysosome immunopurification. **b-i**, LPG (**b** and **e**), BMP (**h** and **i**), hemi-BMP (**c** and **f**) and PG (**d** and **g**) subspecies in whole cell fractions (**b**,**c**,**d**,**h**) and purified lysosomes (**e**,**f**,**g**,**i**) of WT and *CLN8*-KO Hek293 cells (*n* = 3). **i**, Live cell imaging of WT and *CLN8*-KO Hek293 cells. Lysosomes were stained with LysoTracker^TM^ and nuclei were stained with Hoechst. **k**, Quantification of LysoTracker^TM^ signal (*n* = 6 scans per condition). **l**, Representative live cell imaging of *CLN8*-KO Hek293 cells treated with 250 nM Torin1 for 16 h. Nuclei, lysosomes, and phospholipids were stained with Hoechst, LysoSensor^TM^, and Nile red, respectively (data is representative for three independent scans in random well locations). **m**, Normalized phospholipid (Nile red) and LysoSensor^TM^ signal intensity in the linear region of interest (ROI), shown as a white line in l. Scale bars in all images 20 µm. In **b**-**i**, *n* represents independent well replicates; in **k**, *n* represents randomly selected microscopy fields within a well. Data are shown as mean ± s.d. Statistical comparisons in **b**-**i** were performed with unpaired two-tailed Student’s *t* test and in **k** with one-way ANOVA followed by Bonferroni posthoc analysis. *P* values are indicated in the figure panels. Source data and unprocessed blots are available. Source data

To gain further insights into the metabolic function of CLN8, we determined the levels of GPG and various lipids in whole cells and purified lysosomes. Lysosomes were purified from WT and *CLN8*-knockout (KO) Hek293 cells stably expressing the 3xHA-tagged lysosomal membrane protein TMEM192^24^ (Extended Data Fig. 5a), and the enrichment of the lysosomal marker lysosomal associated membrane protein 1 (LAMP1) was confirmed by western blotting (Fig. 3a).

Whole-cell fractions of *CLN8*-KO cells showed a threefold increase in GPG content compared with WT controls. Independent of the genotype, GPG concentrations were lower in lysosomes than in the whole-cell fractions, suggesting that GPG is primarily located outside of acidic organelles (Fig. 3b). LPG was enriched fivefold in lysosomes of WT cells compared to whole cells and decreased by 80% in lysosomal fractions of *CLN8*-KO cells (Fig. 3c). This was mostly due to a reduction in the major LPG subspecies 18:1, while specifically LPG 18:0 was increased (Extended Data Fig. 5b,e). As expected from previous observations, BMP was enriched in the lysosomal fraction of WT cells and absent in *CLN8*-KO cells^22^ (Fig. 3d). Similarly, hemi-BMP was elevated in lysosomes of WT cells and strongly reduced in *CLN8*-KO cells (Fig. 3e). Conversely, PG levels in cells remained unchanged in both fractions irrespective of the genotype (Fig. 3f). Changes in subspecies of different lipid classes are shown in Extended Data Fig. 5b–i.

**Extended Data Fig. 6:**
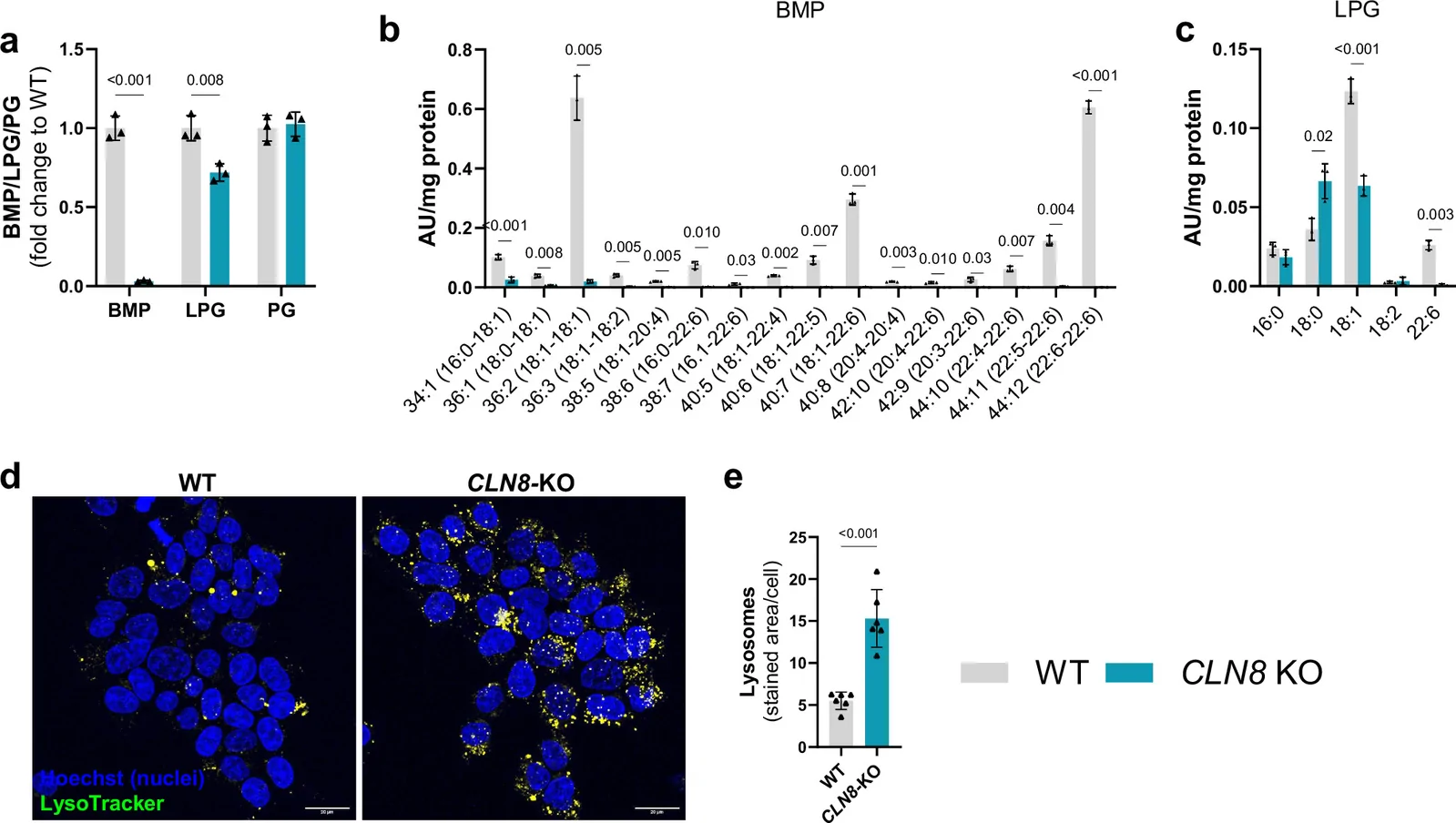
*CLN8*-deficiency disrupts BMP synthesis in Hap1 cells. **a**, BMP, LPG and PG levels in whole cell fractions of WT and *CLN8*-KO Hap1 cells (sum of the analyzed subspecies, n=3). **b,c**, BMP (**b**) and LPG (**c**) subspecies in whole cell fractions of WT and *CLN8*-KO Hap1 cells. (*n* = 3; data are representative of 2 independent experiments). **d**, Live cell imaging of WT and *CLN8*-KO Hap1 cells. Lysosomes were stained with LysoTracker^TM^ and nuclei were stained with Hoechst. Scale bars in all images 20 µm. **e**, Quantification of LysoTracker^TM^ signal (*n* = 6 scans per condition). In **a**-**c**, *n* represents independent well replicates; in **e**, *n* represents randomly selected microscopy fields within a well. Data are shown as mean ± s.d. Statistical comparisons were performed with unpaired two-tailed Student’s *t* test. *P* values are indicated in the figure panels. Source data are available. Source data

Depleted BMP stores, reduced LPG levels and unchanged PG levels were also detected in Hap1 cells lacking CLN8 (hemi-BMP was below the detection limit, Extended Data Fig. 6a). In contrast to the Hek293 cell line, Hap1 cells contained substantial amounts of BMP esterified with docosahexaenoic acid (DHA, 22:6), the major species of testis and brain^25^ (Extended Data Fig. 6b). Notably, these cells contain the presumed precursor LPG 22:6, which was completely absent in *CLN8*-KO cells, while LPG 18:0 was again elevated (Extended Data Fig. 6c).

### *CLN8*-deletion leads to phospholipidosis

BMP promotes lysosomal membrane degradation and lipid export^4^. Therefore, BMP deficiency is expected to compromise membrane catabolism, leading to phospholipidosis. Using the LipidTOX phospholipidosis detection reagent, we observed increased staining of *CLN8*-KO cells, indicating impaired degradation and excessive accumulation of membranes (Fig. 3g,h). In line, LysoTracker imaging in live cells revealed increased staining in *CLN8*-KO Hek293 and Hap1 cells (Extended Data Figs. 5j,k and 6d,e). Importantly, supplementation with BMP, containing 18:1 FA residues in *sn*-3/*sn*-3′ position (*sn*-3,3′*-S*,*S*-BMP), strongly reduced phospholipidosis in *CLN8*-KO cells, suggesting that BMP deficiency is causal for the observed lipid storage disorder (Fig. 3g,h).

Nile red staining, which can discriminate between polar and neutral lipids^26,27^, further confirmed that *CLN8*-KO cells accumulate phospholipids in lysosomes (Fig. 3i,j). This phenotype was exacerbated in the presence of the mTOR complex inhibitor Torin1, which is known to increase the lysosomal turnover of phospholipids^28^ (Fig. 3i,j). Co-staining with LysoSensor revealed that Nile red stained phospholipid deposits colocalize with acidic compartments (Extended Data Fig. 5l,m).

In rescue experiments, the addition of *sn*-3,3′-*S*,*S*-BMP prevented phospholipidosis in *CLN8*-KO cells (Fig. 3g,h), although *sn*-2,2′-*S*,*S* BMP is the native form of BMP^29–31^. Recent studies demonstrated that the 2,2′ acyl-configuration of *S*,*S*-BMP determines its resistance to lysosomal hydrolysis^2^. To compare *sn*-3,3′-*S*,*S*-BMP uptake and remodelling in WT and *CLN8*-KO cells, we analysed the cellular BMP content, FA composition and positional isomers. The addition of BMP strongly increased cellular 18:1–18:1 BMP levels in both genotypes (Fig. 3k). BMP subspecies containing other FAs than 18:1 (18:0, 18:2 and 22:6) increased only in WT cells (Fig. 3k). Analysis of the positional isomers, which separate under the applied chromatography (Extended Data Fig. 1a), revealed that the 3,3′ and 2,3′ BMP were increased 1.7- and 2.6-fold in *CLN8*-KO cells, respectively, compared with WT cells, whereas 2,2′-BMP levels were reduced by 60% (Fig. 3l,m). These observations suggest that CLN8 affects FA composition and positional remodelling of BMP. Nevertheless, *sn*-2,2′*-*BMP isomer levels in supplemented *CLN8-*KO cells increased 2.8-fold above basal WT levels, which was sufficient to rescue the phospholipidosis phenotype.

**Extended Data Fig. 7:**
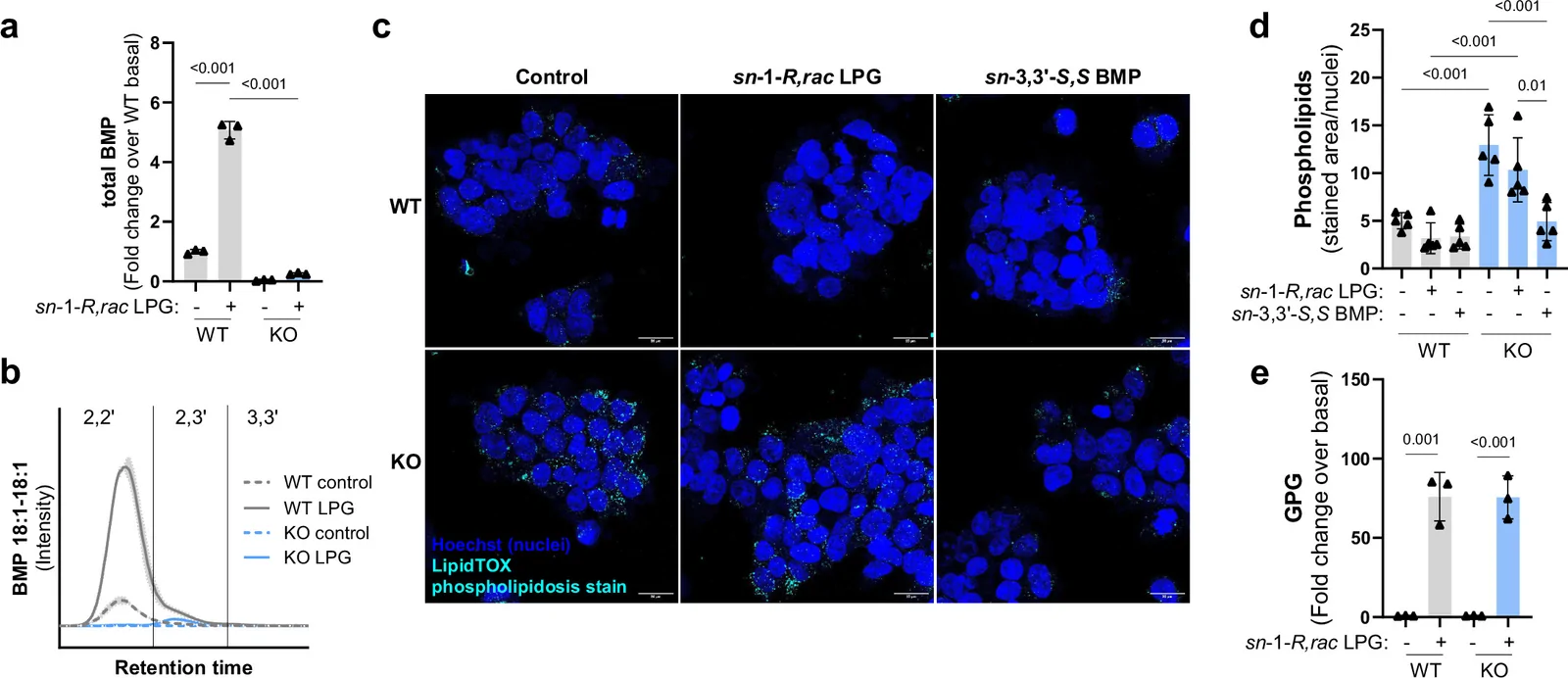
*R*-acyl-LPG supplementation in *CLN8*-deficient cells. **a**, Total BMP levels of WT and *CLN8-*KO Hek293 cultured in the absence and presence of 5 µM *sn-*1-oleoyl-*R,rac* LPG for 72 h (n=3). **b**, Chromatogram of 18:1-18:1 BMP, showing the peaks corresponding to 2,2′, 2,3′ and 3,3′ acyl-configurations, in control and LPG loaded WT and *CLN8*-KO Hek293 cells shown in a (*n* = 3). **c**, Fluorescent imaging of formaldehyde-fixed WT and *CLN8*-KO Hek293 cells stained with the HCS LipidTOX™ Green phospholipidosis detection reagent (cyan) and Hoechst (blue). Cells were cultured in the absence and presence of 5 µM *sn*-3,3′-18:1-18:1-*S,S* BMP (*sn*-3,3′-*S,S* BMP) or 5 µM *sn-*1-oleoyl-*R,rac* LPG (*sn*-1-*R,rac* LPG) for 72 h. **d**, Quantification of the area stained by HCS LipidTOX™ phospholipidosis detection reagent (*n* = 5 scans per condition). **e**, GPG levels of control and 18 h *sn*-1-*R,rac* LPG (50 µM) loaded WT and *CLN8*-KO Hek293 (*n* = 3). In **a**,**b** and **e**, *n* represents independent well replicates; in **d**, *n* represents randomly selected microscopy fields within a well. Data are shown as mean ± s.d. Statistical comparison was performed with one-way ANOVA followed by Bonferroni posthoc analysis. *P* values are indicated in the figure panels. Source data are available. Source data

Previous studies showed that LPG and PG supplementation promotes BMP formation^10,18,32^. To test whether LPG counteracts BMP deficiency in *CLN8*-KO cells, we incubated WT and *CLN8*-KO cells with the commercially available *R*-acylated lipid (*sn-*1-oleoyl-*R*,*rac* LPG). As expected, BMP levels increased fivefold in WT cells upon LPG supplementation (Extended Data Fig. 7a). Analysis of the positional isomers revealed that 90% and 10% of the generated BMP exhibited 2,2′- and 2,3′ acyl-configuration, respectively (Extended Data Fig. 7b). *CLN8*-KO cells showed a 95% reduction in LPG-induced BMP synthesis, reaching 25% of basal WT levels (Extended Data Fig. 7a). The formed BMP exclusively exhibited 2,3′ acyl-configuration (Extended Data Fig. 7b). In contrast to *sn-*3,3′-*S*,*S* BMP, *sn-*1-*R*,*rac* LPG supplementation was not sufficient to rescue phospholipidosis (Extended Data Fig. 7c,d). Together these observations suggests that *sn-*1-*R*,*rac* LPG cannot be converted into mature *sn-*2,2′-*S*,*S* BMP in *CLN8*-KO cells. Notably, exogenous LPG raised the GPG content of WT and of *CLN8*-KO cells 80-fold (Extended Data Fig. 7e), suggesting that the synthesis of 2,2′-BMP in WT cells is driven by the CLN8-dependent utilization of excess GPG.

### CLN8 acylates GPG in vitro and in vivo

**Fig. 4:**
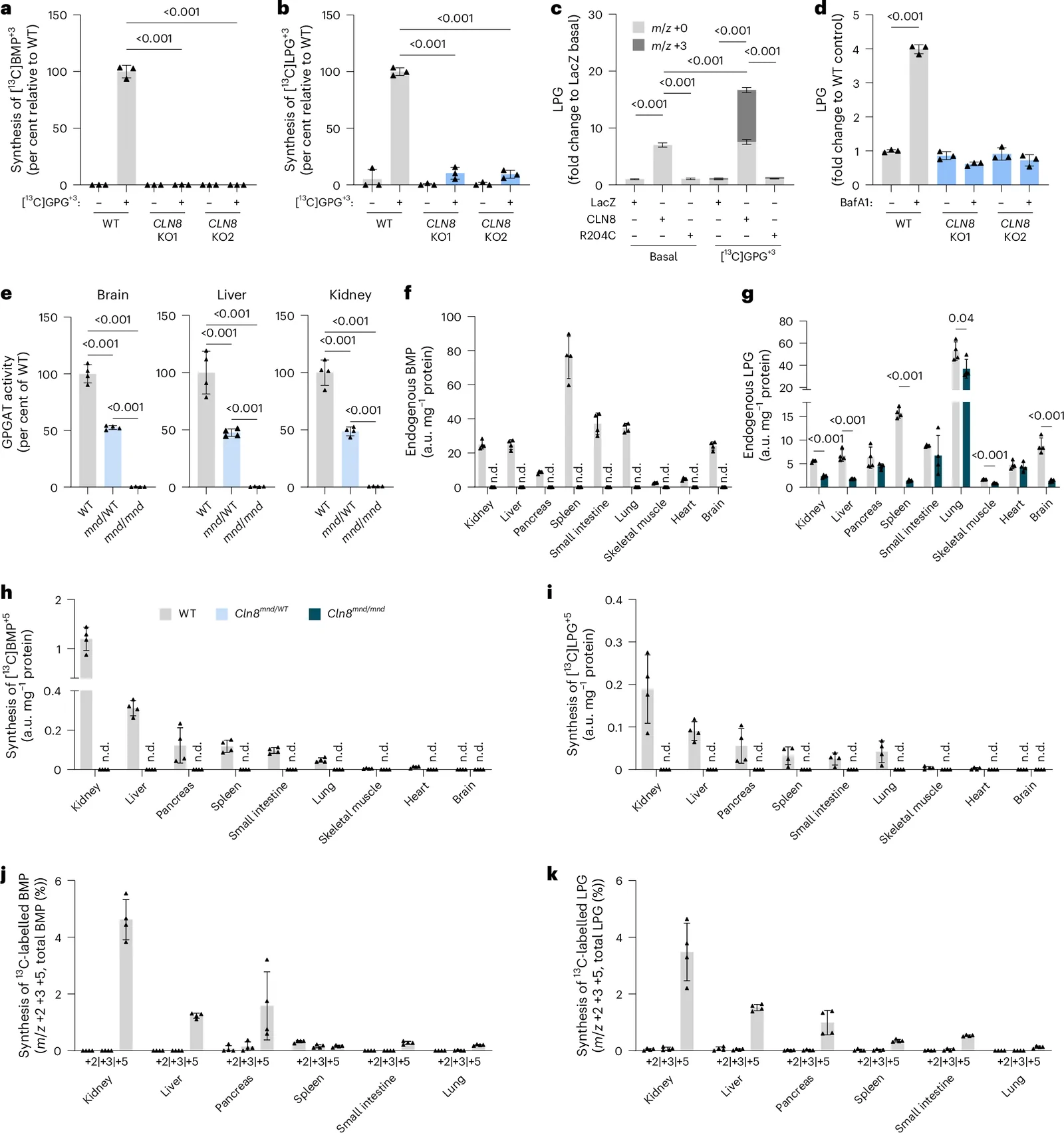
CLN8 enables the utilization of GPG for LPG and BMP synthesis in vitro and in vivo. **a**,**b**, Synthesis of [^13^C]BMP^+3^ (**a**) and [^13^C]LPG^+3^ (**b**) in WT and *CLN8*-KO Hek293 cells in the presence of 250 µM [^13^C]GPG^+3^(18 h incubation; *n* = 3). **c**, LPG levels in LacZ-, CLN8- or R204C-overexpressing Hek293 cells under basal conditions and in the presence of exogenously added [^13^C]GPG^+3^ (250 µM; *n* = 3, labelled and unlabelled LPG species are shown). **d**, Effect of 18 h bafilomycin A1 (0.1 µM; DMSO vehicle control) on LPG levels of WT and *CLN8*-KO Hek293 cells (*n* = 3, representative of two independent experiments). In **a**–**d**, *n* represents independent well replicates. **e**, GPGAT activity in brain, liver and kidney lysates (1,000*g* supernatant) of WT, *Cln8*^*mnd/*WT^ and *Cln8*^*mnd/mnd*^ mice (*n* = 4 mice per genotype). **f**,**g**, BMP (**f**) and LPG (**g**) levels in tissues of WT and *Cln8*^*mnd/mnd*^ mice (*n* = 4 mice per genotype). **h**,**i**, Levels of [^13^C]BMP^+5^ (**h**) and [^13^C]LPG^+5^ (**i**) in tissues of WT and *Cln8*^*mnd/mnd*^ mice 2 h post intraperitoneal injection of 1.1 mg [^13^C]GPG^+5^ per animal (55 mg kg^−1^, *n* = 4 mice per genotype). Data classified as not detectable (n.d.) in **f**,**h**,**i** were below the limit of detection, defined as a S/N ratio <3. Therefore, no statistical analysis was performed. **j**,**k**, Levels of [^13^C]BMP (**j**) and [^13^C]LPG (**k**) with *m*/*z* shifts of +2, +3 or +5, in tissues of WT mice 2 h post intraperitoneal injection of 1.1 mg [^13^C]GPG^+5^ (**h** and **i**). The results are presented as a percentage of labelled lipids relative to total lipids examined (*n* = 4 mice). Lipids are shown as sum of the analysed subspecies. Data are shown as mean ± s.d. Statistical comparison in **a**–**e** was performed with one-way ANOVA followed by Bonferroni posthoc analysis and in **g** with unpaired two-tailed Student’s *t-*test. *P* values are indicated. Source data

**Extended Data Fig. 8:**
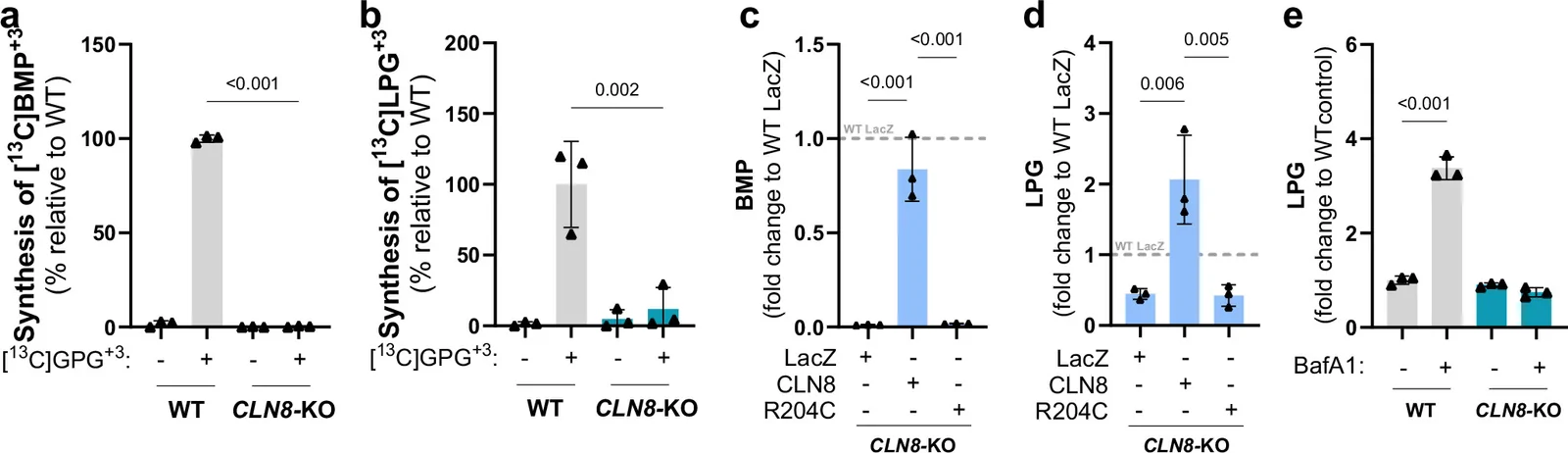
CLN8 enables the utilization of GPG for LPG and BMP synthesis. **a**,**b**, Synthesis of [^13^C]BMP^+3^ (**a**) and [^13^C]LPG^+3^ (**b**) in WT and *CLN8*-KO Hap1 cells in the presence of 250 µM [^13^C]GPG^+3^. Incubations were performed for 18 h under standard cell culture conditions (*n* = 3). **c,d**, BMP and LPG levels in LacZ-, CLN8-, or R204C-overexpressing *CLN8*-KO Hek293 cells. Data are shown as fold-change to WT Hek293 cells expressing LacZ (dashed line; *n* = 3; data are representative of two independent experiments). **e**, Effect of 18 h Bafilomycin A1 treatment (0.1 µM) on LPG levels of WT and *CLN8*-KO Hap1 cells. DMSO was used as vehicle control (*n* = 3). Lipids were analyzed with LC-MS. In **a**-**e**, *n* represents independent well replicates. Data are shown as mean ± s.d. Statistical comparison was performed with one-way ANOVA followed by Bonferroni posthoc analysis. *P* values are indicated in the figure panels. Source data are available. Source data

Incubation of WT cells with [^13^C]GPG resulted in the formation of isotope-labelled LPG, BMP and hemi-BMP (Fig. 1d,h). Accordingly, we next tracked the incorporation of [^13^C]GPG^+3^ into lipids of *CLN8*-KO cells. In contrast to WT controls, mutant Hek293 and Hap1 cells were unable to produce [^13^C]BMP^+3^ (Fig. 4a, Extended Data Fig. 8a) and showed a ~95% reduction in [^13^C]LPG^+3^ formation (Fig. 4b and Extended Data Fig. 8b). Thus, CLN8 is rate-limiting for the incorporation of GPG into phospholipids.

Overexpression of CLN8 in Hek293 cells increased cellular LPG levels ~6- and 16-fold in the absence and presence of exogenously added [^13^C]GPG^+3^, respectively, suggesting that the enzyme acylates GPG from endogenous and exogenous sources. Conversely, expression of the R204C variant had no effect on LPG levels (Fig. 4c and Extended Data Fig. 2g). Re-expression of CLN8 in *CLN8*-KO cells rescued BMP deficiency and led to a fourfold increase of LPG compared with the LacZ control. Again, no effect was observed in R204C-expressing cells (Extended Data Figs. 2h and 8c,d).

Blocking lysosomal acidification with BafA1 led to the extra-lysosomal accumulation of LPG and decreased BMP levels (Fig. 1o,p). *CLN8* deficiency completely prevented LPG accumulation in BafA1-treated Hek293 and Hap1 cells, confirming that CLN8 mediates extra-lysosomal acylation of GPG (Fig. 4d and Extended Data Fig. 8e).

**Extended Data Fig. 9:**
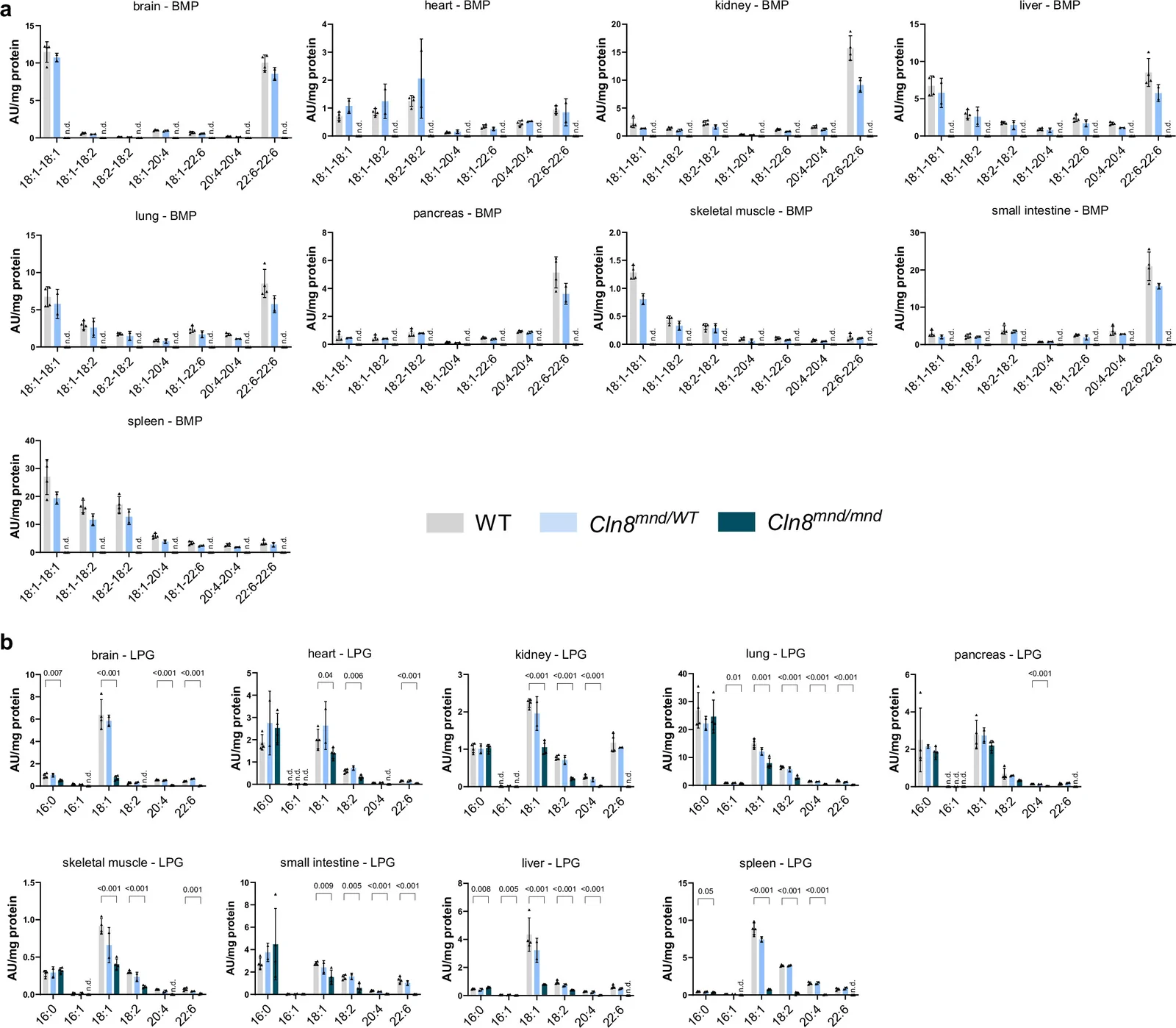
BMP and LPG subspecies in WT, *Cln8*^*mnd*/WT^, and *Cln8*^*mnd*/*mnd*^ mice. **a**, BMP subspecies (*n* = 4 for WT and *Cln8*^*mnd*/*mnd*^, *n* = 2 for *Cln8*^*mnd/*WT^). **b**, LPG subspecies (*n* = 4 for WT and *Cln8*^*mnd*/*mnd*^, *n* = 2 for *Cln8*^*mnd/*WT^). Data classified as n.d. (not detectable) were below the detection limit, defined as a signal-to-noise ratio of less than 3. Data are shown as mean ± s.d. Statistical comparisons between WT and *Cln8*^*mnd*/*mnd*^ were performed with unpaired two-tailed Student’s *t* test. *P* values are indicated in the figure panels. Source data are available. Source data

To investigate the in vivo function of CLN8, we measured GPGAT activity and BMP synthesis in the motoneuron degeneration mouse (*Cln8*^*mnd*^). This Batten disease model carries a loss-of-function frameshift mutation in the *Cln8* gene^14,33^. GPGAT activity in brain, liver and kidney of WT and mutant mouse models correlated with the *Cln8* gene dosage, showing an ~50% reduction and no detectable activity in tissue lysates of heterozygous and homozygous mice (*Cln8*^*mnd/*WT^ and *Cln8*^*mnd*/*mnd*^), respectively, compared with WT controls (Fig. 4e). Lipidomic analysis revealed that BMP was below the detection limit in all analysed tissues of *Cln8*^*mnd*/*mnd*^ mice (Fig. 4f), whereas WT controls and *Cln8*^*mnd/*WT^ mice exhibited similar BMP content (Extended Data Fig. 9a). In addition, we observed strongly decreased LPG levels in brain, kidney, liver, skeletal muscle and spleen of *Cln8*^*mnd*/*mnd*^ mice (Fig. 4g). All analysed BMP and LPG subspecies of WT, *Cln8*^*mnd*/WT^ and *Cln8*^*mnd*/*mnd*^ are listed in Extended Data Fig. 9a,b.

To investigate the utilization of GPG for BMP synthesis, we monitored the incorporation of the [^13^C]GPG^+5^ tracer into tissue BMP and LPG. A total of 2 h post intraperitoneal injection, the incorporation of [^13^C]GPG^+5^ into BMP was highest in kidney and liver of WT mice, followed by pancreas, spleen, small intestine and lung (Fig. 4h). Conversely, the formation of [^13^C]BMP^+5^ was not detectable in *Cln8*^*mnd*/*mnd*^ mice. Irrespective of the genotype, no substantial incorporation into BMP was observed in skeletal muscle, heart and brain of WT mice, suggesting that the tracer is not taken up by myocytes and does not cross the blood–brain barrier. Formation of [^13^C]LPG^+5^ was detected in kidney, liver, pancreas, spleen, small intestine and lung of WT but not *Cln8*^*mnd*/*mnd*^ mice (Fig. 4i). Notably, in all WT tissues except for the spleen, [^13^C]BMP were detected with *m*/*z* +5, but not with *m*/*z* +3 or +2 (Fig. 4j). Similarly, [^13^C]LPG appeared exclusively with *m*/*z* +5 (Fig. 4k), suggesting that GPG is directly acylated for the synthesis of these lipids.

## Discussion

*CLN8* is one of 13 genes associated with Batten disease, an inherited neurodegenerative disorder^34^. The genetically distinct forms are grouped together by similar clinical features and the accumulation of autofluorescent storage material in lysosomes of neuronal cells^35^. The age of onset, severity of symptoms and progression of disease vary between the different types but most commonly begin during childhood. Clinical symptoms include progressive impairment of vision, movement and cognition. Therapy development for Batten disease has been limited because the functions of CLN proteins are only partially understood. Enzyme replacement, gene therapy and stem cell transplantation are considered the most promising options^34^.

Recent studies provided new insights into the pathogenic mechanisms of Batten disease, revealing that cells lacking CLN3, CLN5, CLN8 and CLN11 develop BMP deficiency^9,22,36–38^. BMP acts as a cofactor of lysosomal lipid hydrolases and transport proteins that promote the breakdown and export of lipids^4^. It is therefore reasonable to assume that BMP deficiency impairs lysosomal lipid catabolism in several genetically distinct forms of Batten disease. Our observations suggest that BMP deficiency is causal for lysosomal lipid accumulation in *CLN8*-KO cells, as BMP supplementation rescued the phospholipidosis phenotype. Thus, the supplementation of BMP may represent a therapeutic option for *CLN8* deficiency and other lysosomal storage disorders characterized by BMP deficiency. Although the majority of mechanistic studies were conducted in Hek293 and Hap1 cells, studies in the *mnd* mouse model confirmed that *Cln8* deficiency is associated with the systemic lack of GPGAT activity and BMP in the brain and peripheral tissues. Further studies in Batten disease models are needed to evaluate the therapeutic potential of BMP supplementation.

**Fig. 5:**
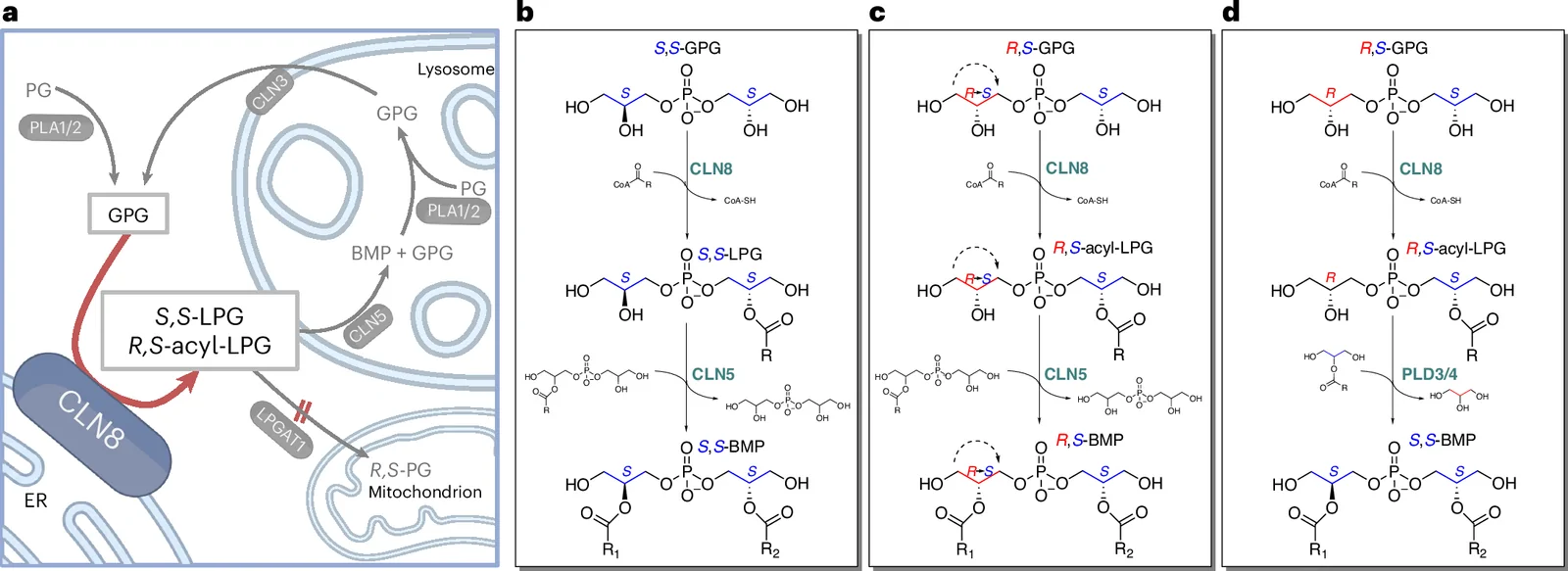
Proposed mechanisms for *S*,*S*-BMP synthesis. **a**, CLN8-mediated acylation of GPG at *S*-linked glycerol moieties generates *S*,*S-*(ref. ^41^) or *R*,*S*-acyl-LPG at the ER, which enables lysosomal BMP but not PG synthesis. CLN8 derived LPG is transported to lysosomes and converted into BMP by CLN5^9^. This reaction, as well as the extra- and intra lysosomal hydrolysis of PG and BMP by phospholipase A1/A2 (PLA1/2), generates GPG. Lysosomal GPG is exported from lysosomes in a CLN3 dependent manner^36^ and can be reacylated by CLN8. **b**, The acylation of *S*,*S*-GPG by CLN8 results in *S*,*S*-LPG, which is converted into *S*,*S*-BMP by CLN5 without stereoconversion^41^. **c**, CLN8-derived *R*,*S*-acyl-LPG can be converted into *R*,*S*-BMP by CLN5. Here, an intramolecular stereoconversion is necessary to generate mature *S*,*S*-BMP. The intermediate undergoing stereoconversion and the underlying molecular mechanism has not yet been identified. **d**, CLN8-derived *R*,*S*-acyl-LPG can be converted into *S*,*S*-BMP via a transphosphatidylation reaction, catalysed by lysosomal PLD3/4. This reaction uses *R*,*S*-acyl-LPG as phosphatidyl donor and monoacylglycerol as acceptor, leading to the formation of *S*,*S*-BMP and to the release of the *R*-linked glycerol unit^10^. Schematic in **a** created in BioRender; Zimmermann, R. https://biorender.com/l57i8gq (2026).

Our study suggests that CLN8 produces LPG at the ER, which is selectively delivered to late endosomes/lysosomes and incorporated into BMP (Fig. 5a). GPGAT activity is essential for BMP synthesis, as *CLN8*-KO cells and mice lack BMP. CLN8-derived LPG is acylated on *S*-linked glycerol moieties and can therefore not be incorporated into PG and CL, which contain FA residues exclusively at the *R*-position. The treatment of cells with BafA1 prevented the formation of BMP and led to extra-lysosomal accumulation of LPG in WT cells but not CLN8-KO cells. This observation suggests that BMP synthesis requires lysosomes, whereas the BMP precursor *S*-acyl-LPG is produced at the ER. BMP formation in acidic organelles can be catalysed by CLN5^9^. This transacylase utilizes LPG as both FA donor and acceptor, generating BMP and GPG. *CLN5*deficiency compromises BMP synthesis and is associated with elevated lysosomal LPG levels^9^. Conversely, we found that lysosomal LPG levels are decreased in *CLN8*-deficient cells. Together, these observations indicate that CLN5 uses CLN8-derived LPG as substrate for BMP synthesis.

Although this study did not address the transport of LPG between the ER and lysosomes, our findings suggest the existence of mechanisms involving either vesicular or non-vesicular transport. Previous work demonstrated that CLN8 is an ER-associated transmembrane protein that regulates lysosomal biogenesis by promoting the ER-to-Golgi transfer of lysosomal enzymes produced at the ER. This observations indicates an additional function of CLN8 that is independent of its enzymatic activity^39,40^. Currently, it is unknown whether the total loss of CLN8 protein and the expression of inactive variants produce different phenotypes. However, inactive CLN8 variants with a point mutation in the active centre cause Batten disease^15,16,22^, suggesting that the enzymatic activity is essential for lysosomal homeostasis. We show that overexpression of the inactive Batten disease-associated CLN8 variant R204C cannot rescue BMP deficiency.

CLN8 requires GPG as acyl acceptor, which may derive from PG or BMP. In accordance with an independent study^41^, we identified CLN8 as a stereospecific GPG acyltransferase, which preferentially mediates the acylation of *S-*linked glycerol moieties of GPG (Fig. 5a). Starting from BMP-derived *S*,*S-*GPG, the CLN8 reaction delivers *S*,*S-*LPG that can be utilized for the synthesis of mature *S*,*S-*BMP by CLN5 without the need for stereoconversion (Fig. 5b). Notably, previous studies suggested that lysosomes are a metabolic source of GPG. CLN3 deficiency, the most common Batten disease subtype, is associated with both the accumulation of GPG in lysosomes and BMP deficiency^36^. These observations support a model in which GPG, deriving either from CLN5- or phospholipase A1/2-catalysed reactions, is exported from lysosomes via CLN3 and re-esterified by CLN8 at the ER (Fig. 5a). The reduced availability of GPG could contribute to the development of BMP deficiency in cells lacking CLN3.

The acylation of PG-derived *R*,*S-*GPG produces *R*,*S*-LPG with the acyl chain linked to the *S-*glycerol (*R*,*S-*acyl-LPG, Fig. 5c,d). This intermediate can be converted by CLN5 into *R*,*S*-BMP, suggesting that additional steps are required to produce mature *S*,*S*-BMP. At present, published data support two different models of *S*,*S*-BMP synthesis. The first mechanism is based on tracer studies suggesting an intramolecular stereoconversion of the *R*-linked glycerol moiety and no exchange of backbone glycerols^18,42^. The underlying molecular mechanism and the intermediate in which the stereoconversion takes place and has yet to be identified (Fig. 5c). The second mechanism of *S*,*S*-BMP synthesis is based on a transphosphatidylation reaction using *R*,*S-*acyl*-*LPG as phosphatidyl donor and monoacylglycerol as acceptor (Fig. 5d). This reaction is mediated by lysosomal phospholipases D3/D4 (PLD3/4) and leads to the loss of the *R*-linked glycerol^10^ (Fig. 5d). The required *R*,*S-*acyl-LPG precursor can be delivered either by the hydrolysis of *R*,*S*-BMP by acid phospholipase A1/2 (PLA2G15^2^) or by CLN8. Our experiments revealed that the glycerol moieties of GPG are not exchanged during BMP synthesis in vitro and in vivo, arguing for an intramolecular isomerization. However, it must be considered that experiments were performed with a racemic GPG mixture containing *S*,*S-*GPG, which can be utilized for the synthesis of *S*,*S-*BMP without stereoconversion (Fig. 5b). Furthermore, the GPG tracer was unable to cross the blood–brain barrier, preventing insights into mechanisms promoting *R*,*S* to *S*,*S* conversion in neuronal cells. Thus, it is possible that both intramolecular stereoconversion and transphosphatidylation mechanisms contribute to *S*,*S*-BMP synthesis. In order to fully elucidate the underlying molecular mechanisms of *S*,*S*-BMP synthesis in neuronal and peripheral cells, tracer studies using stereochemically pure *R*,*S*- and *S*,*S*-GPG, as well as analytical methods distinguishing between the stereoisomers of BMP and its precursors, are required.

In summary, our data suggest that CLN8 catalyses the stereospecific acylation of the *S*-linked glycerol of GPG at the ER, which directs LPG into BMP synthesis and prevents its incorporation into other lipids. *CLN8* deficiency results in depleted BMP stores in vitro and in vivo, which compromises lysosomal membrane catabolism and leads to phospholipidosis. We conclude that GPG acylation and BMP synthesis are interrelated metabolic processes that are highly relevant for lysosomal lipid homeostasis and the development of neurodegenerative disease. To our knowledge, previous studies have not reported that GPDs can be directly acylated for lipid synthesis in mammalian cells, although GPD acylation provides a direct synthesis route that is less energy-intensive than de novo synthesis and allows FA remodelling of membrane lipids in *sn*-1 and *sn*-2 positions. Related metabolic lipid synthesis pathways have been described in yeast and plants that recycle GPC for PC synthesis^43,44^.

## Methods

Our research complies with all relevant ethical regulations: Cambridge Animal Welfare and Ethics Board and UK Home Office (PPL number PP1740969).

### Reagents, materials and antibodies

Detailed information on all reagents, materials, plasmids and antibodies used during this study are listed in Supplementary Tables 1–3. All reported oligonucleotides were synthetized by Eurofins Genomics.

### Cell culture

For regular maintenance, Hek293 (CRL-1573, ATCC) or Hap1 (C631, Horizon) cells were cultured in DMEM 4.5 g l^−1^ glucose or IMDM (both Gibco), respectively, both supplemented with 10% heat-inactivated fetal bovine serum as well as 100 IU ml^−1^ penicillin and 100 IU ml^−1^ streptomycin. For heat-inactivation, fetal bovine serum was prewarmed to 37 °C and subsequently incubated for 1 h at 56 °C in a water bath.

Expi293F cells (A14527, Gibco) were cultured in Expi293T Expression Medium (A1435101, Gibco) in 25-ml or 10-ml culture volumes using 125-ml vented Erlenmeyer flasks (431143, Corning) or 50-ml CELLSTAR cell reactor tubes (227245, Greiner Bio-One) on an orbital shaker platform at 125 or 220 rpm, respectively. Cell lines were regularly checked for mycoplasma contamination using the Mycoplasmacheck Service from Eurofins Genomics GmbH. All cells were grown under standard conditions (37 °C, 95% humidified atmosphere, 7% CO_2_).

### Animals

*Cln8*^*mnd*^ mice (B6.KB2-Cln8^*mnd*^/MsrJ; JAX strain number 001612), were maintained in a SOPF facility on a regular light–dark cycle (12 h light, 12 h dark) at a room temperature (RT, 23 ± 1 °C) and fed ad libitum on a standard laboratory chow diet (SAFE 105). Homozygous experimental animals were generated by crossing heterozygous parents. Animals were killed by cervical dislocation. All studies involving animals are reported in accordance with the ARRIVE guidelines. Experimental procedures on mice were approved by the University of Cambridge Animal Welfare and Ethics Board and UK Home Office (PPL number PP1740969) and were conducted in accordance with the EU Directive 2010/63. All animal procedures were guided by the 3R principles (replacement, reduction, refinement) to minimize pain, suffering and distress.

### Transfection of Expi293, Hek293 and Hap1 cells

To generate cell lysates containing recombinantly expressed GPAT1-4, LacZ, LPGAT1, CLN5, CLN8 or CLN8 R204C for in vitro assays or protein purification, Expi293F cells were transfected according to the manufacturer’s instructions and as previously described^45^.

For transient protein expression in Hek293 and Hap1 cells, 6 × 10^6^ cells were seeded into 100-mm culture dishes and transfected with the Lipofectamine 3000 transfection reagent (L3000001, Invitrogen) the following day. One 100-mm dish was transfected according to the following protocol: 600 μl DMEM without antibiotics and FCS (DMEM−/−) premixed with 30 μl Lipofectamine 3000 were pooled with DMEM−/− containing 15 μg plasmid DNA and 30 μl P3000 reagent. The solution was incubated for 15 min at RT and added dropwise to the medium. For cell culture experiments, the cells were trypsinized 24 h post transfection, resuspended in fresh medium and seeded into six-well plates at a density of 0.7 × 10^6^ cells per well. Cells were collected 48 h post transfection. All plasmids used for transfection are listed in Supplementary Table 2.

### Cell culture experiments

All cell culture experiments were conducted in 6- or 12-well plates with an initial seeding density of 1 × 10^6^ or 0.5 × 10^6^ cells per well, respectively.

For isotope tracer experiments, the cells were seeded into six-well plates and cultured overnight. On the next day, the cells were incubated for 18 h with 500 µM [^13^C]GPG^+3^, 250 µM [^13^C]GPG^+5^, 500 µM [U-^13^C]glycerin (489476, Sigma-Aldrich) or DMEM where glucose was replaced by 25 mM [U-^13^C]glucose (389374, Sigma-Aldrich). Cells cultured in standard cultivation medium were used as controls.

For isotope tracer experiments with inhibitors, both Hek293 and Hap1 cells were seeded in six-well plates. The next day, the cells were preincubated for 1 h with 10 µM triacsinC (T4540, Sigma-Aldrich), 100 nM bafilomycin A1 (HY-100558, MedChemExpress) or dimethylsulfoxide (DMSO) as vehicle control and further cultured for 18 h in the presence of 500 µM [^13^C]GPG^+3^ maintaining constant inhibitor concentrations.

Isotope tracer experiments with *CLN8*-KO Hek293 and Hap1 cells were performed by seeding cells in 12-well plates. The following day cells were cultured in the presence of 250 µM [^13^C]GPG^+3^ for 18 h.

For BMP or LPG supplementation experiments, WT and *CLN8*-KO Hek293 cells were seeded in 100-mm dishes. The next day, cells were cultured in medium supplemented with and without 5 µM *sn*-3,3′-*S*,*S*-18:1–18:1 BMP (857135, Avanti Polar Lipids) or *sn-*1-oleoyl-*R*,*rac* LPG (858125, Avanti Polar Lipids). BMP and LPG were emulsified in media via brief sonication in a water bath. After 24 h, cells were reseeded into 12-well plates and cultured for another 48 h in the presence of 5 µM BMP or LPG with medium change every other day.

Before lipidomic analysis, the cells were washed twice with PBS and collected by gentle flushing in PBS. Cellular lipids were extracted as described and endogenous as well as isotope-labelled lipids were analysed by LC–MS.

### Purification of 6xHis-tagged CLN8 and CLN8 R204C

CLN8 and CLN8 R204C were isolated using 6xHis affinity chromatography. Therefore, half of a cell pellet from a 25-ml culture of Expi293 cells containing recombinantly expressed proteins was resuspended in 6-ml lysis buffer composed of 50 mM HEPES (pH 7.25), 0.1 mM EDTA, 5 mM β-mercaptoethanol, 5% glycerol, 150 mM NaCl, a protease inhibitor cocktail (1 µg ml^−1^ pepstatin, 2 µg ml^−1^ antipain and 20 µg ml^−1^ leupeptin) and 1.5% lauryl maltose neopentyl glycol (LMNG; A50940, Thermo Scientific). The suspension was incubated on ice for 2 h, with occasional inversion of the tube to ensure thorough lysis. The lysate was centrifuged at 40,000*g* for 30 min at 4 °C to remove insoluble material. The resulting supernatant was diluted to 50 ml with lysis buffer lacking LMNG. This solution was mixed with 250 µl of prewashed (3× in PBS) PureCube 100 Ni-NTA agarose beads (74103, Cube Biotech) and incubated overnight at 4 °C on an overhead shaker. The following day, the agarose beads were transferred to a gravity column and washed in five steps. Each washing buffer contained 50 mM HEPES pH 7.25, 0.1 mM EDTA, 5 mM β-mercaptoethanol, 5% glycerol, 1 mM DTT, 0.1% LMNG, protease inhibitor cocktail, imidazole and NaCl. The concentration of imidazole and NaCl in the washing buffers were as follows: imidazole (mM)/NaCl (mM): buffer 1: 50/500; buffer 2: 20/500; buffer 3: 10/500; buffer 4: 0/250; buffer 5: 0/125. The beads were initially washed with washing buffer 1 in 500-µl steps until no more protein was detected in the washing fractions using the Bradford reagent. After that, the beads were washed consecutively with 3 ml of washing buffer 2–5. Elution of the purified proteins was performed in 20× 100-µl steps using an elution buffer containing 50 mM HEPES pH 7.25, 125 mM NaCl, 0.1 mM EDTA, 5 mM β-mercaptoethanol, 5% glycerol, 300 mM imidazole, 1 mM DTT, the protease inhibitor cocktail and 0.05% LMNG. All protein-containing fractions were pooled, analysed for protein concentration using the Bradford reagent, aliquoted, snap-frozen in liquid N_2_ and stored at −80 °C. The purification steps and the purity of the isolated proteins (10 µl of each fraction) were analysed by SDS–polyacrylamide gel electrophoresis followed by Coomassie staining and western blotting.

### CRISPR–Cas9-mediated deletion of *CLN8*

*CLN8*-deficient Hek293 and Hap1 cells were generated by CRISPR–Cas9 using the pSpCas9(BB)-2A-Puro (PX459) V2.0 vector (gift from Feng Zhang, Addgene, plasmid #62988) and 5′-CTGCATGCCGACAAGGCGCGTGG-3′ as sgRNA, which was designed using chopchop^46^. The sgRNA was cloned into the *BbsI* site of pSPCas9(BB)-2A-Puro V2.0. Correct insertion was verified by Sanger sequencing. Hek293 and Hap1 cells were transfected with the resulting construct using the Lipofectamine 3000 Reagent (L3000001, Invitrogen) as described above. Cells transfected with empty pSPCas9(BB)-2A-Puro V2.0 served as control. A total of 24 h after transfection, 2 µg ml^−1^ puromycin were added for the selection of successfully transfected cells. After clonal expansion for two-three weeks, the cell lines were screened for mutations in the *CLN8* gene by sequencing a PCR product containing the sgRNA target site (forward primer 5′-GTCGCTGGCTTTGTCTTCTACT-3′; reverse primer 5′-CTTTAAGAGCATCCAGGAAACG-3′). Control cells transfected with an empty vector were subjected to the same procedure, including passaging and validation of the genomic sequence by Sanger sequencing.

### Chemical synthesis of [^13^C]GPG^+3^, [^13^C]GPG^+5^ and generation of [^13^C]GPG^+3/PG^ from headgroup labelled PG

A detailed procedure and validation on the chemical synthesis of [^13^C]GPG^+3^ and [^13^C]GPG^+5^ is provided as Supplementary Note 1.

[^13^C]GPG^+3/PG^ was generated via saponification of headgroup-labelled di-oleoyl [^13^C]PG. Di-oleoyl [^13^C]PG was synthesized by the conversion of di-oleoyl PC into di-oleoyl PG by cabbage phospholipase D (PLD) (P8398, Sigma Aldrich) in the presence of [U-^13^C]glycerin as described previously^18^.

For saponification, 1 mg of di-oleoyl [^13^C]PG was dissolved in 60 µl of methyl *tert*-butyl ether/methanol (3/1, v/v) and mixed with 30 µl 2 M NaOH for 2 h at RT. The solution was neutralized by the addition of 30 µl 2 M HCl. After further adding 340 µl methyl *tert*-butyl ether/methanol (3/1, v/v) and 60 µl ddH_2_O, the solution was vortexed and centrifuged at 20,000 rpm for 5 min. The upper organic phase was quantitatively removed and the aqueous phase containing, GPG was dried at 45 °C under a stream of N_2_. The dried extract, including NaCl generated during saponification, was reconstituted in PBS, and the concentration of GPG was determined by comparing the LC–MS signal against the chemically synthesized [^13^C]GPG^+5^.

### Microscopy

All microscopy glass slides used for culturing cells, were precoated with poly-D-lysine (A-003-M, Sigma-Aldrich).

For LysoTracker Red DND-99 (L7528, Invitrogen) staining in live cells, 350,000 WT and *CLN8*-KO Hek293 and Hap1 cells were seeded into 35-mm glass bottom dishes (81218-200, Ibidi). The next day, cells were cultured for 1 h in medium containing 62 nM Lysotracker and 1 µg ml^−1^ Hoechst 3342 (ab228551, Abcam). The experiment was timed between conditions to ensure uniform exposure of cells to the fluorescent dyes.

For phospholipidosis staining, Hek293 WT and *CLN8*-KO cells were cultured with and without 5 µM *sn*-3,3′-*S*,*S*-18:1–18:1 BMP (857135, Avanti Polar Lipids) or 5 µM *sn*-1-oleoyl-*R*,*rac* LPG (858125, Avanti Polar Lipids) in 100 m culture dishes. Lipids were emulsified in DMEM medium via brief sonication. After 24 h, the cells were reseeded in eight-well chamber slides (94.6170.802, Sarstedt) at a density of 100,000 cells per well. The following day, the cells were incubated with medium containing HCS LipidTOX Green phospholipidosis detection reagent (H34350, Invitrogen) for another 24 h. Throughout all steps, lipid supplementation was maintained at 5 µM. For imaging, the cells were fixed with 4% PBS buffered formaldehyde and stained with 1 µg ml^−1^ Hoechst 3342.

For Nile red staining, Hek293 WT and *CLN8*-KO cells were seeded in eight-well chamber slides at a density of 100,000 cells per well. On the next day, the cells were cultured with DMEM medium containing 250 nM Torin1 (475991, Sigma-Aldrich) or DMSO vehicle for 16 h. For imaging, the cells were fixed with 4% PBS-buffered formaldehyde and subsequently stained with 1 µg ml^−1^ Nile red (72485, Sigma-Aldrich) and 1 µg ml^−1^ Hoechst 3342. Colocalization experiments were performed in live cells by culturing Torin1-treated *CLN8*-KO cells for 1 h in medium containing 1 µg ml^−1^ Nile red, 1 µg ml^−1^ Hoechst 3342 and 1 µM LysoSensor Green DND-189 (L7535, Invitrogen).

Imaging data were acquired with a Leica SP8 confocal microscope with spectral detection (Leica Microsystems), using an HC PL APO CS 63×/1.2 numerical aperture water immersion objective. Scans were performed in sequential mode to avoid fluorescence bleed-through into different channels. Excitation (Ex) of fluorescent dyes and recording of their fluorescent emission (Em) were conducted at the following wavelengths: Hoechst 3342: Ex 405 nm, Em 420-452 nm; HCS LipidTOX Green: Ex 488 nm, Em 507–549 nm; LysoTracker Red DND-99: Ex 561 nm, Em 574–666 nm; Nile red—phospholipids: Ex 561 nm, Em 620–660 nm; Nile red—neutral lipids: Ex 514 nm, Em 534–574 nm. The imaging data were acquired in *z*-stacks, with the exception of the colocalization studies. For image quantification, the open-source software Fiji was used. The fluorescent channels were split, and *z*-stacks were converted into a maximum intensity projection. Threshold values were set individually for each channel and kept constant for all images throughout all conditions. Based on the threshold, a mask was created before particles were analysed using the built-in ‘Analyze particle’ function, with a particle size ranging from 0 to infinity structures. Nuclei in each scan were counted manually. To determine the average fluorescent area/per cell, positive areas were summed and divided by the number of nuclei in each image. To visualize colocalization of the Nile red phospholipid and LysoSensor signals, the individual and merged fluorescent channels are shown. In addition, the fluorescent intensities of both signals along a linear region of interested were normalized to respective maximum values and plotted against the region of interested distance.

### In vivo tracer experiments with [^13^C]GPG^+5^

In vivo tracer experiments were performed with female WT and *Cln8*^*mnd*/*mnd*^ mice, aged 10 weeks. [^13^C]GPG^+5^ was dissolved in PBS and administered by intraperitoneal injection at a dosage of 1.1 mg [^13^C]GPG^+5^ per animal (55 mg kg^−1^ bodyweight). Tissues were collected 2 h post injection and snap-frozen in liquid N_2_. A total of 10–20 mg of pulverized tissues were used for lipid analysis. Incorporation of the tracer into phospholipids was determined as described in the mass spectrometry chapter.

### Western blotting

Protein samples were mixed with SDS-sample buffer (0.05 M Tris–HCl, 0.1 M DTT, SDS 2% w/v, 1.5 mM bromophenol blue, 1.075 M glycerol). Samples used for the detection of CLN8 were not subjected to heat denaturation at 95 °C, because this leads to aggregation of CLN8 and prevents the protein from entering the gel. SDS–gel electrophoresis was performed in Tris–glycine buffer (20 mM Tris, 160 mM glycine, 0.083% SDS w/v) using a 10% polyacrylamide gel. The Color Prestained Protein Standard Broad Range (P7719S, New England Biolabs) was used as molecular weight reference. Proteins were transferred onto a polyvinylidene fluoride membrane via the wet blotting method in CAPS buffer (10 mM CAPS, 10% v/v, methanol, pH 11). The membrane was blocked with 10% blotting grade milk powder diluted in Tris-buffered saline with Tween20 (20 mM Tris, 150 mM NaCl, 0.1% Tween 20, pH 7.4). Primary antibodies and antibody-host targeting secondary antibodies used for immunological detection and are listed in Supplementary Table 3.

### Site directed mutagenesis

Mutagenesis was performed using the Q5 site-directed mutagenesis kit (E0552S, New England Biolabs) according to the manufacturer’s instructions using the following primers: forward: 5′-GTTTCACTGCtgcATGGTTCTAA-3′; reverse: 5′-ATGTGAATCATCAGCCAC-3′. This mutation exchanges the conserved arginine 204 with a cysteine (R204C). Annealing temperatures were set according to the NEBaseChanger tool. Elongation time was set to 310 s (~20–30 s kb^−1^). Constructs were transformed into chemically competent *Escherichia coli*. The base-exchange was verified by Sanger sequencing.

### Lysosome immunopurification

To enable lysosome immunopurification (lyso IP), we first generated Hek293 WT and *CLN8*-KO cell lines stably expressing the lysosomal transmembrane protein TMEM192-HA using lentiviral transduction as previously described^45^. To generate lentiviral particles, Hek293T cells (CRL-3216, ATCC) were transfected with the lentiviral packaging vectors psPAX2 and pMD2.G (both gifts from Didier Trono, Addgene plasmids #12260 and #12259) as well as the pLJC5-Tmem192-3xHA (gift from David Sabatini, Addgene plasmid #102930). Hek293 WT and *CLN8*-KO cell lines expressing TMEM192-3xHA were maintained in culture medium additionally supplemented with 2 µg ml^−1^ puromycin to ensure stable protein expression.

Lyso IP was performed according to published protocols with minor adaptions^24^. First, Hek293 WT and *CLN8*-KO cells (pool of two *CLN8*-KO clones) were seeded in 145-mm dishes at a density of 1 × 10^7^ cells per dish. After reaching confluency, cells were washed twice with 5 ml ice-cold PBS and collected in 950 µl of ice-cold KPBS (136 mM KCl, 10 mM KH_2_PO_4_, pH 7.25) using a cell scraper. The cells were centrifuged at 1,000*g* for 2 min at 4 °C and resuspended in 950 µl fresh KPBS. Thereafter, 25 µl of this suspension were snap frozen in liquid N_2_ as the whole-cell fraction. With the remaining solution, cell lysis was performed by aspirating it 25× up and 25× down through a 26G needle. The 1,000*g* supernatant (centrifugation for 2 min at 4 °C) was transferred into a low protein binding tube (90410, Thermo Scientific) containing 50 µl of prewashed Pierce Anti-HA-magnetic beads (88837, Thermo Scientific). After a 3–5-min incubation on an orbital shaker, the tubes were placed on an EasyEights EasySep magnet (18103, STEMCELL Technologies), and the supernatant was removed. The beads were washed twice with 950 µl KPBS and transferred into a fresh low protein binding tube. Beads were washed one more time and subsequently transferred into a 2-ml safe-lock tube. The supernatant was discarded and purified lysosomes were snap frozen in liquid N_2_ for lipid extraction or western blot analysis. All procedures except the incubation on the orbital shaker were performed on ice. The lyso IP protocol was performed with four samples in parallel (2× WT, 2× *CLN8*-KO). For the western blot analysis, whole-cell fractions and purified lysosomes were resuspended in 100 µl RIPA buffer (150 mM NaCl, 10 mM Tris, 0.5 mM EDTA, 0.5% NP-40) and protease inhibitor cocktail (1 µg ml^−1^ pepstatin, 2 µg ml^−1^ antipain, 20 µg ml^−1^ leupeptin) and incubated on ice for 1 h. The samples were centrifuged at 15,000*g* for 10 min at 4 °C, and the supernatant was transferred into a fresh low protein binding tube. Finally, 15 µl of each fraction (whole cell and purified lysosomes) were used for western blot analysis.

### Preparation of cell lysates

Pulverized tissues samples (~20 mg) were homogenized using an T10 basic Ultra-Turrax (IKA) or with ceramic beads (1.4 mm) in a bead mill homogenizer for 30 s in 0.5 ml lysis solution (250 mM sucrose, 1 mM EDTA, 1 mM DTT) supplemented with protease inhibitor (1 µg ml^−1^ pepstatin, 2 µg ml^−1^ antipain, 20 µg ml^−1^ leupeptin). The cells were lysed in 0.2–0.5 ml lysis solution using the SONOPULS ultrasonic homogenizer (Bandelin) for 3× 10 s with an amplitude of 20%. The cell and tissue homogenates were centrifuged at 1,000*g* for 10 min (4 °C) to remove nuclei and debris. The supernatants (infranatant for adipose tissue lysates) were collected and adjusted for equal protein content by determining the protein concentration using the Bradford reagent.

### In vitro activity assays

GPGAT assays with Expi293 cell lysates containing recombinantly expressed GPAT1-4 were performed under conditions optimized for GPAT enzymes^47^. To this end, 20 µl of the cell lysates (2 mg ml^−1^ protein concentration) were incubated for 30 min at 37 °C with 180 µl substrate solution containing 75 mM Tris–HCl buffer (pH 7.5) supplemented with 800 µM [^13^C]GPG^+3^ as acyl acceptor, 80 µM 16:0- or 18:1-CoA as acyl donor, 1 mM DTT, 1 mg ml^−1^ FA free BSA and 4 mM MgCl_2_.

All other GPGAT activity assays were performed with a substrate solution containing 100 mM Bis–Tris–propane buffer (pH 7) supplemented with 1% FA-free BSA, 1 mM DTT, 100 µM oleoyl-CoA as acyl donor and 100 µM [^13^C]GPG^+3^ as acyl acceptor, unless otherwise stated.

As source of enzymatic activity, we used 20 µl of tissue/cell lysates (1 mg ml^−1^ protein concentration) or 6 µl of purified CLN8 or CLN8 R204G (1 µg each), which we incubated with 80 µl substrate solution for 1 h at 37 °C in a water bath under mild agitation. Assays with purified proteins and Expi293 cell lysates containing recombinantly expressed CLN8 were incubated for 20 min at 37 °C to ensure linearity of the reaction. For kinetic measurements, 16:0-, 18:1- or 18:2-CoA were used at increasing concentrations, while the concentration of [^13^C]GPG^+3^ remained at 100 µM. Comparison of GPGAT and LPGAT activity of purified CLN8 was performed using either 100 µM [^13^C]GPG^+3^ or 100 µM *sn*-1-oleoyl-*R*,*rac*-LPG (858125, Avanti Polar Lipids) as acyl acceptor. To measure the stereospecificity of CLN8, 4 µg of purified CLN8 protein (24 µl) were incubated for 1 h at 37 °C with a substrate containing 200 µM [^13^C]GPG^+3/PG^ as acyl acceptor, which was generated via saponification of head group labelled PG as described above. The determination of the head group specificity of purified CLN8 was performed by using 100 µM of commercially available GPE (Cay34460, Cayman Chemical), GPC (G5291, Sigma-Aldrich) or custom generated [^13^C]GPG^+3^, GPI and GPS as acyl acceptor. GPI and GPS were prepared via saponification of PI and PS as described above for [^13^C]GPG^+3/PG^.

To investigate whether LPGAT1 or CLN5 can utilize the CLN8 reaction product as a substrate for PG or BMP synthesis, we first prepared Expi293 lysates containing recombinantly expressed CLN5 and sonicated membrane preparations of Expi293 cells containing recombinantly expressed LPGAT1 or CLN8 and used these as a source of enzymatic activity. To isolate membranes, cell lysates (2 mg ml^−1^ protein concentration) were centrifuged at 100,000*g* for 1 h. The membrane pellet was subsequently emulsified in equal amount of lysis solution via sonication. For the assay, 20 µl of membranes containing recombinantly expressed CLN8 were first incubated with 80 µl substrate containing 100 µM oleoyl-CoA and [^13^C]GPG^+5^ as described above. After 20 min, the reaction was heat inactivated for 10 min at 85 °C and centrifuged at 10,000*g* for 3 min to pellet the protein precipitate. Next, the reaction, including the precipitate was further combined with either 20 µl of recombinant LPGAT1-containing membranes or 20 µl Expi293 cell lysates containing recombinant CLN5. The reactions with LPGAT1 were additionally supplemented with 10 µl substrate containing 18:1-CoA (80 µM) as acyl donor with and without *sn*-1-oleoyl-*R*,*rac*-LPG (8 µM) as acyl acceptor. For reactions with CLN5, the assay pH was adjusted to ~6 by adding 80 µl 100 µM MES buffer (pH 5.5). Both, LPGAT1 and CLN5 reactions were incubated for another 20 min at 37 °C.

All activity assays were stopped either by snap freezing in liquid N_2_ or by the addition of lipid extraction solution (‘Lipid extraction’ section). The reaction products were analysed by LC–MS.

### Lipid and GPD extraction

#### Lipid extraction

Samples of in vitro assays, cell pellets and pulverized tissues (10–20 mg) were extracted in 2-ml safe lock tubes using a modified protocol of Matyash et al.^48^, with 1 ml extraction solution containing methyl *tert*-butyl ether/methanol (3/1, v/v), 0.01% (w/v) butylated hydroxytoluene, 0.01% (v/v) acetic acid and the following internal standards: 150 pmol 17:1 LPG (858127), 150 pmol 14:0–14:0 BMP (857131), 150 pmol 14:0–14:0–14:0 hemi-BMP (857132), 150 pmol 14:0–14:0 PG (840445), 133 pmol 17:0–17:0 PE (830756), 30 pmol 17:0–17:0 PS (840028), 8 pmol 17:1 LPC (855677), 30 pmol 17:1 LPE (856707), 50 pmol 17:1 LPS (858141, all Avanti Polar Lipids), 50 pmol 17:0–17:0 PC (37-1700, Larodan). Total lipid extraction was performed under constant shaking for 30 min at RT. After the addition of 200 μl ddH_2_O (100 µl for in vitro assays) and further shaking for 10 min at RT, samples were centrifuged at 14,000 rpm for 5 min at RT to enable phase separation. For the isolation of lipids, 800 µl of the upper organic phase were collected and dried under a stream of N_2_. For LC–MS analysis, the lipids were resolved in 100 µl 2-propanol/methanol/ddH_2_O (7/2.5/1, v/v/v).

#### GPD extraction

To extract cellular GPDs, the extraction solution (see above) was supplemented with 50 pmol (per millilitre) custom synthesized *sn*-[U-^13^C]glycero-3-phosphocholine ([^13^C]GPC^+3^) as internal standard. The extraction was performed as described above. After phase separation, the upper organic phase was quantitatively removed, and 200 µl of MeOH were added to the aqueous phase followed by a brief vortex. The mixture was centrifuged at 20,000 rpm for 5 min, and 350 µl of the supernatant were transferred into a fresh tube. The solution was dried under a stream of N_2_ at 45 °C and reconstituted in 50 µl MeOH for LC–MS analysis.

The extracted cell proteins from lipid and GPD isolations were dried and solubilized in NaOH/SDS (0.3 M/0.1% w/v) at 65 °C, and the protein content was determined with the Pierce BCA reagent (23225, Thermo Fisher Scientific) and used for normalizing the of LC–MS measurements.

### Targeted LC–MS analysis

Liquid chromatography was performed using an Agilent 1290 Infinity II UHPLC equipped with an ACQUITY UPLC BEH C18 Column 2.1 mm × 150 mm, 1.7 µm (186002353, Waters Corporation) for separation of lipids or with a Kinetex HILIC column 100 mm × 2.1 mm, 1.7 µm, 100 Å (00D-4474-AN, Phenomenex) for GPD analysis. The column temperature was set to 50 °C, and 2 µl sample volume were injected.

For the analysis of polyglycerophospholipids—including LPG, BMP, PG, hemi-BMP and CL—the mobile phases consisted of MeOH/H_2_O (8:2, v/v) as phase A and 2-propanol/MeOH (8:2, v/v) as phase B, both containing 10 mM ammonium acetate, 0.1% formic acid and 8 µM phosphoric acid. Chromatographic separation was carried out at a flow rate of 0.2 ml min^−1^ over a 30-min gradient. The gradient started with a linear increase from 50% to 60% phase B over 13 min, followed by a linear ramp to 100% phase B over 7 min. This was maintained isocratically for 5 min before re-equilibration at 50% phase B for 5 min.

Shorter chromatography gradients, using the same mobile phases, were applied depending on the specific experimental setup and targeted lipid species. For samples targeting LPG, BMP and PG, an 18-min gradient was used. For in vitro assays where only LPG was analysed, a 10-min gradient was used. In both shortened protocols, the runs began at 50% phase B, followed by a linear increase to a higher B percentage, an isocratic hold and a final re-equilibration step to restore initial conditions.

For the analysis of glycerophospholipids—including LPC, LPE, LPS, PC, PE and PS—separation was performed using water (H_2_O, mobile phase A) and 2-propanol (mobile phase B), both supplemented with 10 mM ammonium acetate, 0.1% formic acid and 8 µM phosphoric acid. The flow rate was set to 0.15 ml min^−1^. The 30 min gradient began at 50% B for 0.5 min, increased to 80% B over 8.5 min, then ramped to 100% B over the following 13 min. The column was held at 100% B for 2.5 min before returning to 50% B for re-equilibration.

For GPD measurements mobile phase A consisted of 10 mM ammonium acetate and 0.05% formic acid in LC–MS grade water, while mobile phase B was 100% LC–MS grade acetonitrile. Elution was carried out isocratically at 50% B for 6 min at a flow rate of 0.22 ml min^−1^.

All analytes were detected using an Agilent 6470 triple quadrupole mass spectrometer equipped with an Agilent Jet Stream electrospray ionization (ESI) source. Data acquisition was performed using MassHunter Data Acquisition software (version B.10, Agilent Technologies).

Lipid species including LPG, BMP, PG, hemi-BMP and CL were analysed in negative ion mode, whereas LPC, LPE, LPS, PC, PE, PS and monoacylglycerol fragments of LPG were measured in positive ion mode.

GPDs were analysed using fast polarity switching, allowing simultaneous acquisition of positive and negative ions in a single run. Unit resolution was used in both MS1 and MS2 stages. All precursor-to-product ion transitions and collision energies are detailed in Supplementary Table 4. The Reporting Checklist of the Lipidomics Standards Initiative is available at https://doi.org/10.5281/zenodo.21218517.

Lipid and GPD identification were performed using MassHunter Qualitative Analysis software (version 10.0, Agilent Technologies) and quantified using Skyline (v.24.1.0.414). Data were normalized to analyte-to-internal standard ratios and reported as arbitrary units (a.u.) per mg of protein. Where applicable, absolute quantification was achieved using external calibration with 18:1–18:1 BMP or 18:1 LPG (Avanti Polar Lipids), and results were expressed as picomole per milligram protein.

For lipidomic measurements of tissues from WT, *Cln8*^WT/*mnd*^ and *Cln8*^*mnd*/*mnd*^ mice, the signal-to-noise (S/N) ratios were calculated for each lipid species using Agilent MassHunter Quantitative Analysis 10.1 (Agilent Technologies), with the Peak-to-Peak from Drift S/N algorithm. Noise was determined as the peak-to-peak difference in the baseline region near each species, corrected for slow baseline drift. S/N ratios were calculated as the ratio of peak apex height to the baseline noise. Manual adjustment of integration boundaries was performed where necessary, and S/N values were recalculated after such adjustments. S/N data are provided in the Source Data. Peaks with S/N <3 were considered below the limit of detection.

To calculate isotope-tracer incorporation, the expected abundance of naturally occurring isotopes was first calculated from the molecular formula of each analysed lipid species using the Agilent MassHunter Isotope Distribution Calculator (version 10.0.10305.0). As an example, for PG 16:0–18:1 the calculated contribution to the *m*/*z*+3 signal is 0.0228, indicating that 2.28% of the unlabelled lipid is inherently detected in the *m*/*z*+3 channel.

To correct for this contribution, the corresponding *m*/*z*+0 signal of, in this case, PG 16:0/18:1 was multiplied by this factor, and the resulting value was subtracted from the measured *m*/*z*+3 signal. After correction, the *m*/*z*+3 signal in control cells was typically reduced to values at or near zero, whereas tracer-treated cells retained a clearly detectable signal. In rare cases, the corrected *m*/*z*+3 signal in control cells remained slightly above zero. To account for these instances, this residual value was additionally subtracted from the isotope-corrected signal of the tracer-treated cells to ensure accurate quantification of tracer incorporation.

The relative amount of isotope labelled lipids was determined by calculating the ratio of labelled lipids to total lipids (sum of endogenous and labelled lipids).

### Mass spectrometry proteomics

Total cell lysates (5% SDS, 50 mM AMBIC) were processed using S-Trap micro columns (C02-micro-80, Protifi) following the high recovery protocol recommended by the manufacturer and using trypsin (90059, Pierce) as the carrier protein. Mass spectrometry samples were analysed on timsTOF ion mobility mass spec- trometer (Bruker) in-line a reversed-phase C 18 Aurora column (25 cm × 75 μm) on a UltiMate 3000 UHPLC system (Thermo) as described previously^49^. The spectra were recorded in DIA mode and processed with DIA-NN (DIA-NN 2.3.0 Academia) using a synthetic library created from UniProt protein database (human reviewed, 2024_07_29).

### Data visualization

Data visualization was performed using Prism version 10.5.1. ChemDraw version 23.1.1 was used to visualize chemical structures in Figs. 1a,d–h and 5b–d. BioRender was used to create the schematics in the illustrations for Fig. 5a (Zimmermann, R. https://BioRender.com/l57i8gq (2026)).

### Statistics and reproducibility

Statistical analysis was performed using Prism version 10.5.1. Data are presented as mean ± standard deviation (s.d.), unless stated otherwise. The number and identity of replicates, the number of independent experimental repeats and the statistical tests used are reported in Figs. 1–4 and Extended Data Figs. 1–9; significance levels are shown as *P* values and are reported in the Source Data. Data distribution was assumed to be normal but this was not formally tested. The individual datapoints are shown in Figs. 1–4 and Extended Data Figs. 1,3,5–9. No statistical methods were used to predetermine sample sizes, but our sample sizes are similar to those reported in previous publications. No data were excluded from the analyses. The experiments were not randomized. The Investigators were not blinded to allocation during experiments and outcome assessment.

Information on experimental repeats: Fig. 1: results in Fig. 1b,c,e,h,j,n are from a single experiment. Results in Fig. 1i,k–m,o,p are from a single experiment, similar results were obtained in HAP1 cells (data shown in the Source Data for Fig. 1). Results in Fig. 1d,f are representative of two independent experiments. Fig. 2: results in Fig. 2a,b,d–f,h–o are from a single experiment. Results in Fig. 2c,g are representative of three and two independent experiments, respectively. Fig. 3: LysoIP data in Fig. 3a–f are from a single experiment. Whole-cell data in Fig. 3b–f are representative of two independent experiments. Results in Fig. 3g–jare representative of three and two independent experiments, respectively. Results in Fig. 3k–m are from a single experiment, similar results were obtained following a 96 h incubation (data shown in the Source Data for Fig. 3). Fig. 4: results in Figs. 4a–k are from a single experiment. Results in Fig. 4d are representative of two independent experiments. Extended Data Fig. 1: results in Extended Data Fig. 1a–c are from a single experiment. Results in Extended Data Fig. 1d ([^13^C]GPG^+3^) are representative of two independent experiments. Extended Data Fig. 2: results in Extended Data Fig. 2a,b,d–g are from a single experiment. Results in Extended Data Fig. 2c,h are representative of two independent experiments. Extended Data Fig. 3: results in Extended Data Fig. 3a,b are from a single experiment. Extended Data Fig. 4: results are from a single experiment. Extended Data Fig. 5: Results in Extended Data Fig. 5a–m are from a single experiment. Extended Data Fig. 6: Results in Extended Data Fig. 6a–c are representative of two independent experiments. Results in Extended Data Fig. 6d,e are from a single experiment. Extended Data Fig. 7: results in Extended Data Fig. 7a,b are from a single experiment; similar results were obtained using 50 µM LPG and an 18 h incubation period (data shown in the Source Data for Extended Data Fig. 7). Results in Extended Data Fig. 7c–e are from a single experiment. Extended Data Fig. 8: results in Extended Data Fig. 8a,b,e are from a single experiment. Results in Extended Data Fig. 8c,d are representative of two independent experiments. Extended Data Fig. 9: results are from a single experiment.

### Reporting summary

Further information on research design is available in the Nature Portfolio Reporting Summary linked to this article.

## Online content

Any methods, additional references, Nature Portfolio reporting summaries, source data, extended data, supplementary information, acknowledgements, peer review information; details of author contributions and competing interests; and statements of data and code availability are available at https://doi.org/10.1038/s41556-026-02059-8.

## Supplementary information

Supplementary Information Supplementary Note 1, detailed procedure and validation on the chemical synthesis of [^13^C]GPG^+3^ and [^13^C]GPG^+5^.

Reporting Summary

Peer Review File

Supplementary Table 1–5 Tables for reagents, materials, plasmids, antibodies, targeted MS precursor-to-product ion transitions and retention times, and light microscopy reporting table.

Supplementary Data 1 Unprocessed targeted lipidomics datasets from all cell-based and animal experiments presented in the Figs. 1,3,4 and Extended Data Figs. 5–9.

## Source data

Source Data Fig. 1 Numerical source data for Fig. 1.

Source Data Fig. 2 Numerical source data for Fig. 2.

Source Data Fig. 3 Numerical source data for Fig. 3.

Source Data Fig. 4 Numerical source data for Fig. 4.

Source Data Extended Data Fig. 1 Numerical source data for Extended Data Fig. 1.

Source Data Extended Data Fig. 3 Numerical source data for Extended Data Fig. 3.

Source Data Extended Data Fig. 4 Numerical source data for Extended Data Fig. 4.

Source Data Extended Data Fig. 5 Numerical source data for Extended Data Fig. 5.

Source Data Extended Data Fig. 6 Numerical source data for Extended Data Fig. 6.

Source Data Extended Data Fig. 7 Numerical source data for Extended Data Fig. 7.

Source Data Extended Data Fig. 8 Numerical source data for Extended Data Fig. 8.

Source Data Extended Data Fig. 9 Numerical source data for Extended Data Fig. 9.

Unprocessed gels and blots Unprocessed gels and blots.

## Data Availability

Unprocessed lipidomics data are available in the Supplementary Information and the corresponding target list is provided in Supplementary Table 4. The mass spectrometry proteomics data have been deposited to the ProteomeXchange Consortium via the PRIDE partner repository at https://www.ebi.ac.uk/pride with the dataset identifier PXD081638. All data supporting the findings of this study are available from the corresponding author (robert.zimmermann@uni-graz.at) on reasonable request. Source data are provided with this paper.
