# Supplementary material for: Batten disease protein CLN8 enables a non-canonical phospholipid synthesis pathway
Source: Nat Cell Biol. 2026 Sep 15;28(10):2078–87. doi: 10.1038/s41556-026-02059-8 (PMC13661288; doi:10.1038/s41556-026-02059-8)

Extended data fig. 2a

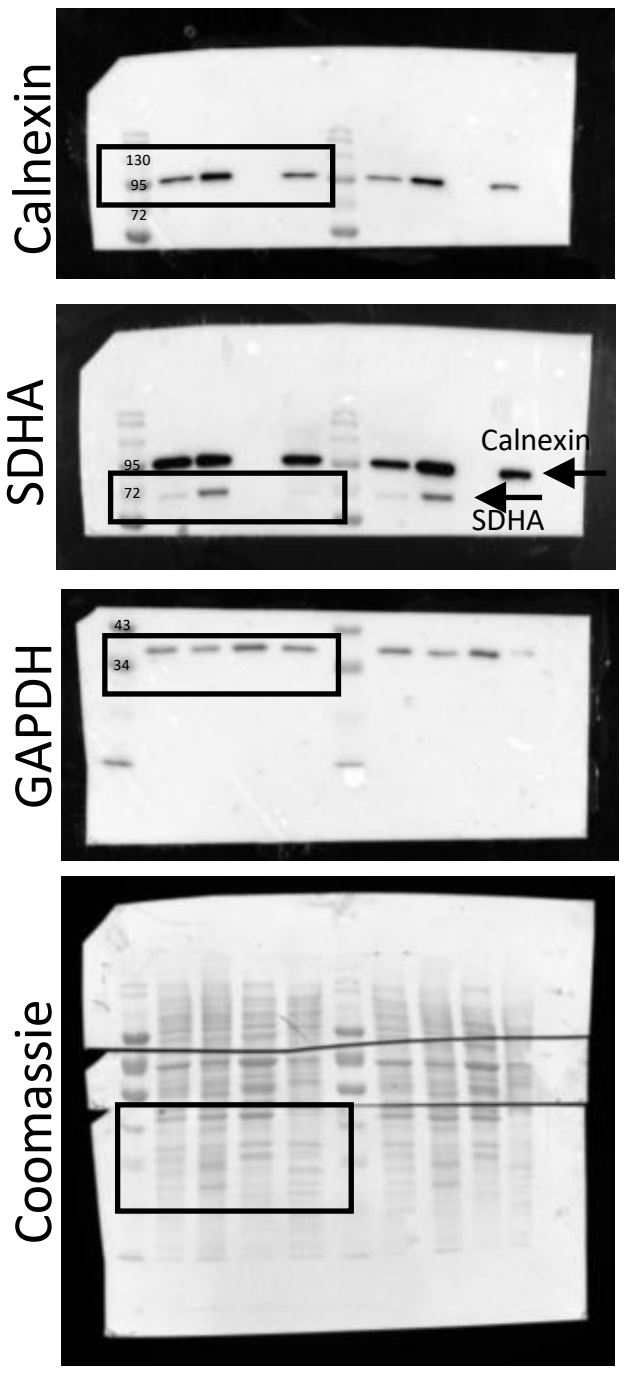

Extended data fig. 2b

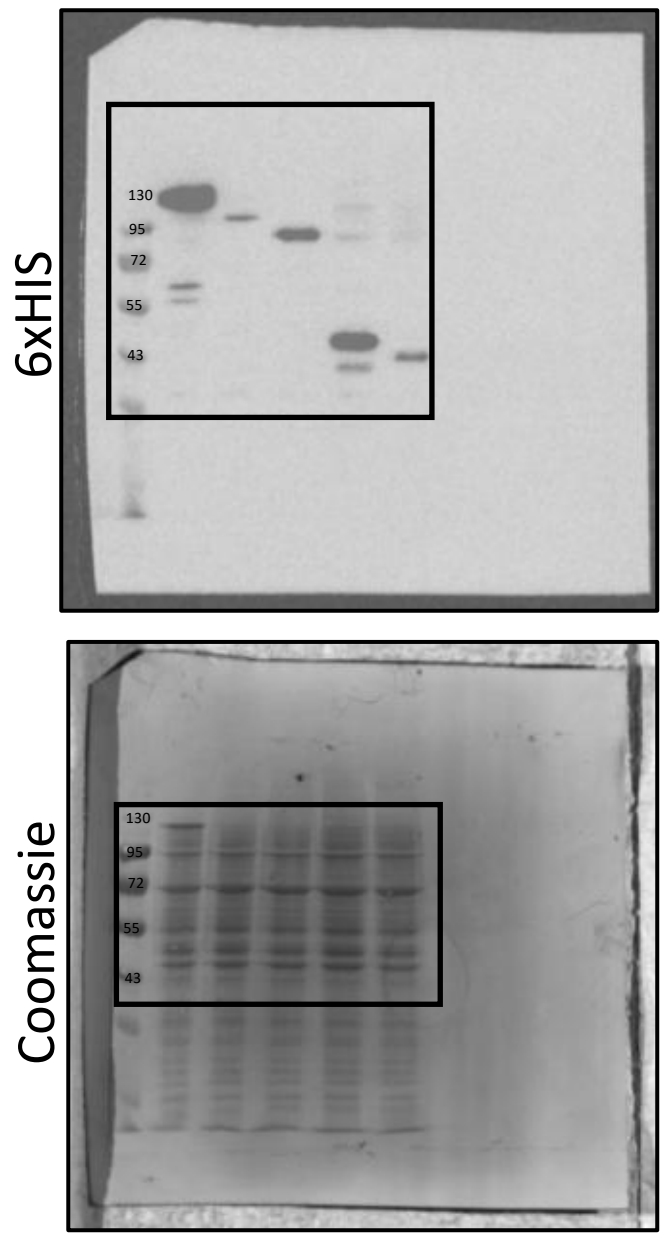

Extended data fig. 2c and 2g

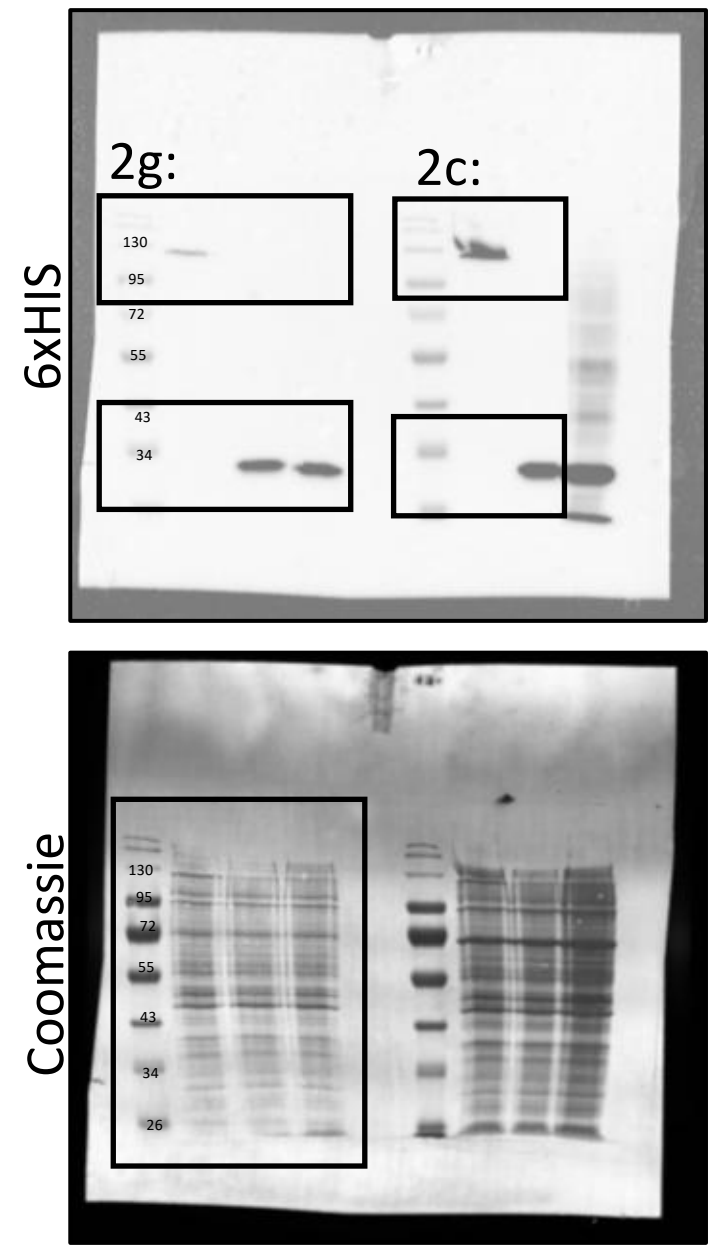

Extended data fig. 2d

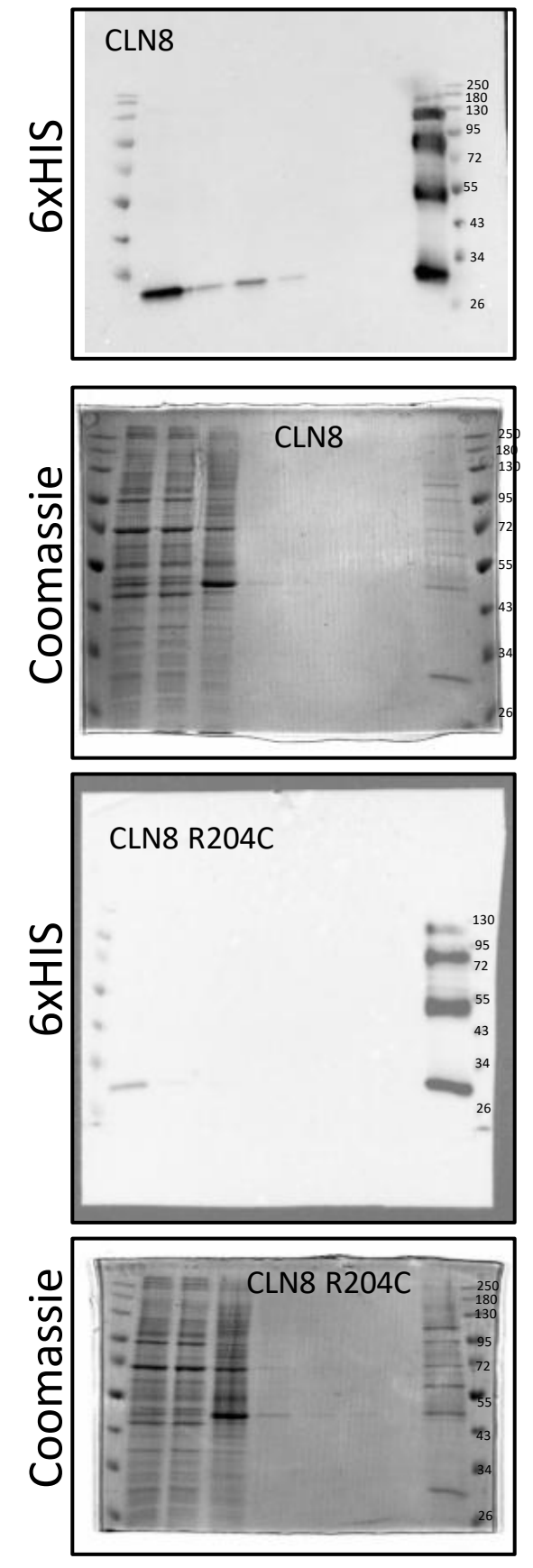

Extended data fig. 2e

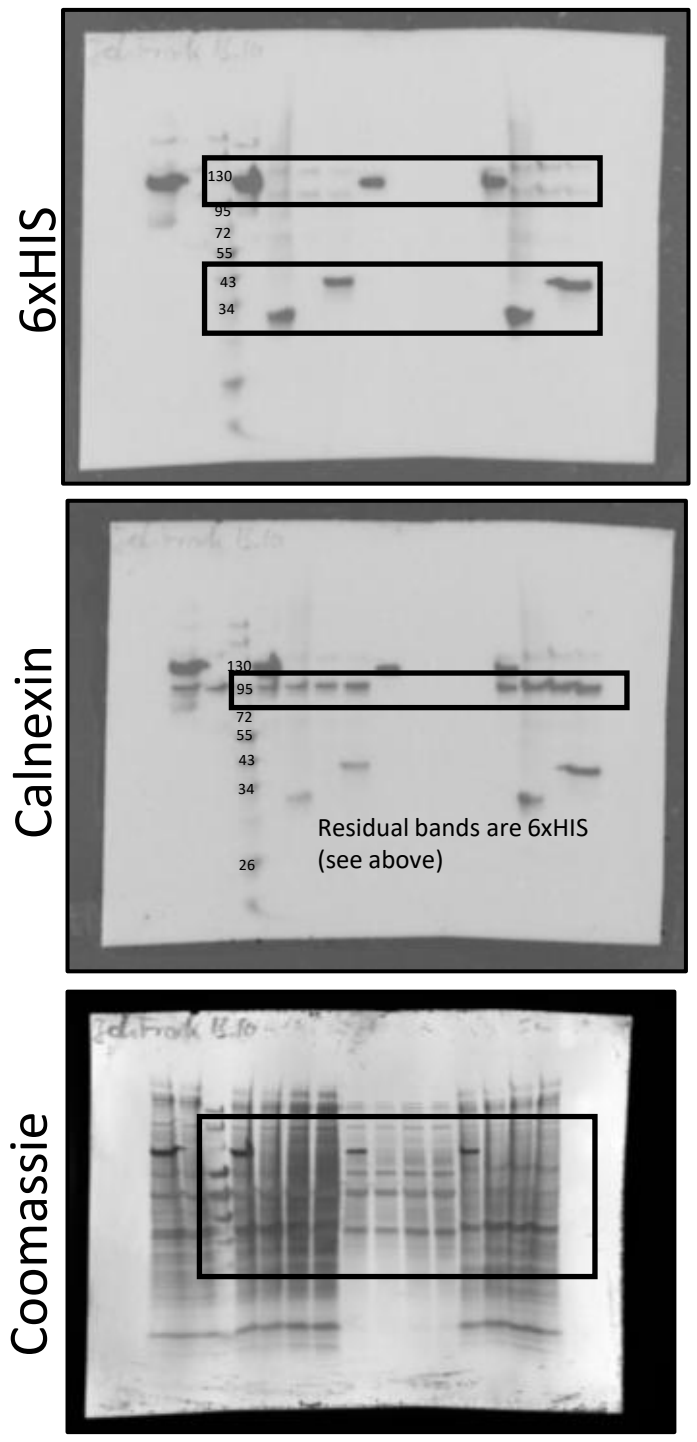

Extended data fig. 2f

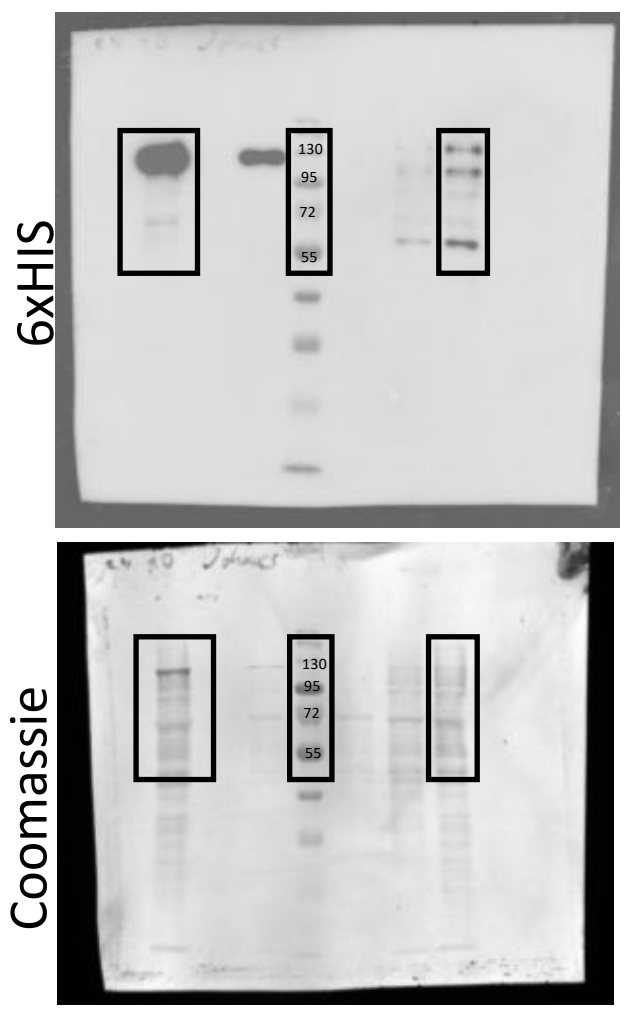

Extended data fig. 2h

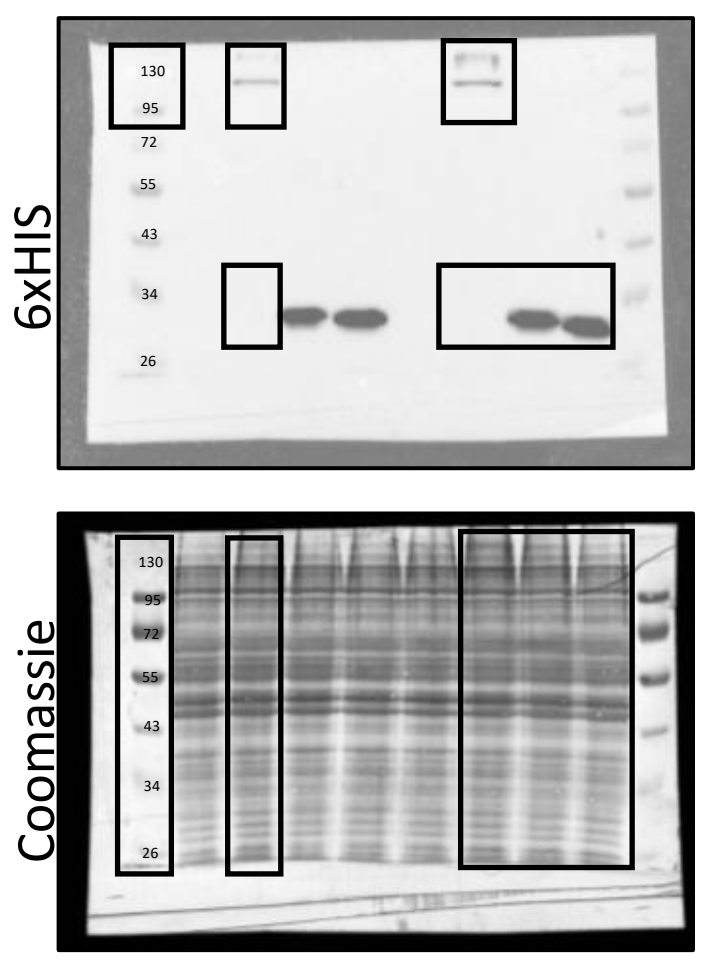

Extended data fig. 5a

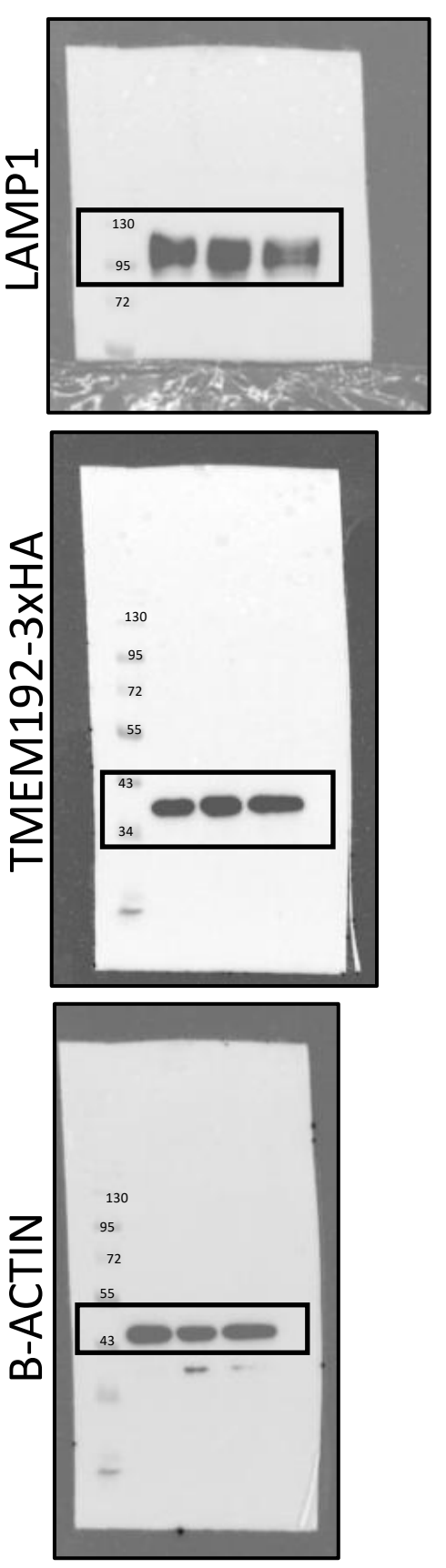

Fig. 3a

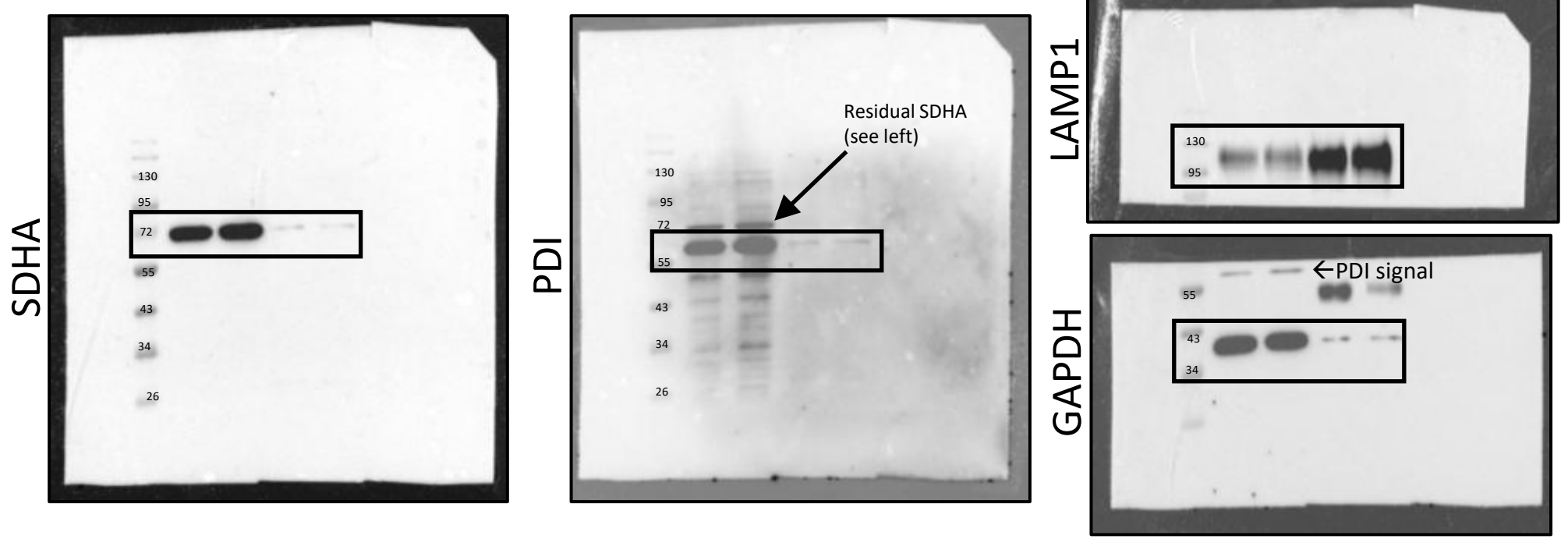

Supplement: Supplementary file 1 [file 41556_2026_2059_MOESM18_ESM.pdf]
