# Supplementary material for: Batten disease protein CLN8 enables a non-canonical phospholipid synthesis pathway
Source: Nat Cell Biol. 2026 Sep 15;28(10):2078–87. doi: 10.1038/s41556-026-02059-8 (PMC13661288; doi:10.1038/s41556-026-02059-8)
Supplement: Supplementary file 3 [file 41556_2026_2059_MOESM2_ESM.pdf]

nature portfolio

Corresponding author(s): Robert Zimmermann

Last updated by author(s): Jul 2, 2026

# Reporting Summary

Nature Portfolio wishes to improve the reproducibility of the work that we publish. This form provides structure for consistency and transparency in reporting. For further information on Nature Portfolio policies, see our Editorial Policies and the Editorial Policy Checklist.

## Statistics

For all statistical analyses, confirm that the following items are present in the figure legend, table legend, main text, or Methods section.

| n/a | Confirmed | |
|---|---|---|
| ☐ | ☒ | The exact sample size (*n*) for each experimental group/condition, given as a discrete number and unit of measurement |
| ☐ | ☒ | A statement on whether measurements were taken from distinct samples or whether the same sample was measured repeatedly |
| ☐ | ☒ | The statistical test(s) used AND whether they are one- or two-sided<br>*Only common tests should be described solely by name; describe more complex techniques in the Methods section.* |
| ☒ | ☐ | A description of all covariates tested |
| ☒ | ☐ | A description of any assumptions or corrections, such as tests of normality and adjustment for multiple comparisons |
| ☐ | ☒ | A full description of the statistical parameters including central tendency (e.g. means) or other basic estimates (e.g. regression coefficient) AND variation (e.g. standard deviation) or associated estimates of uncertainty (e.g. confidence intervals) |
| ☐ | ☒ | For null hypothesis testing, the test statistic (e.g. *F*, *t*, *r*) with confidence intervals, effect sizes, degrees of freedom and *P* value noted<br>*Give P values as exact values whenever suitable.* |
| ☒ | ☐ | For Bayesian analysis, information on the choice of priors and Markov chain Monte Carlo settings |
| ☒ | ☐ | For hierarchical and complex designs, identification of the appropriate level for tests and full reporting of outcomes |
| ☒ | ☐ | Estimates of effect sizes (e.g. Cohen's *d*, Pearson's *r*), indicating how they were calculated |

*Our web collection on statistics for biologists contains articles on many of the points above.*

## Software and code

Policy information about availability of computer code

| | |
|---|---|
| Data collection | Mass spectrometry data acquisition was performed using MassHunter Data Acquisition software (version B.10, Agilent Technologies).<br>Imaging data aquisition was perfomred using Laica Application Suite X software (version 3.5.7.23225, Leica Microsystems).<br>Proteomics spectra were recorded in DIA mode and processed with DIA-NN (DIA-NN 2.3.0 Academia) using a synthetic library created from UniProt protein database (human reviewed, 2024_07_29). |
| Data analysis | Data visualization and statistical analysis were performed using Prism version 10.5.1. Mass spectrometry data analysis were performed using MassHunter Qualitative Analysis software (version 10.0, Agilent Technologies), and Skyline (version 24.1.0.414). For image quantification, the open-source software Fiji was used. |

For manuscripts utilizing custom algorithms or software that are central to the research but not yet described in published literature, software must be made available to editors and reviewers. We strongly encourage code deposition in a community repository (e.g. GitHub). See the Nature Portfolio guidelines for submitting code & software for further information.

## Data

Policy information about availability of data

All manuscripts must include a data availability statement. This statement should provide the following information, where applicable:

- Accession codes, unique identifiers, or web links for publicly available datasets
- A description of any restrictions on data availability
- For clinical datasets or third party data, please ensure that the statement adheres to our policy

There are no restrictions on the data associated with this study. All necessary source data is available as supplementary information. The study does not include any large datasets stored in public repositories. Further requests to robert.zimmermann@uni-graz.at.

## Research involving human participants, their data, or biological material

Policy information about studies with human participants or human data. See also policy information about sex, gender (identity/presentation), and sexual orientation and race, ethnicity and racism.

| | |
|---|---|
| Reporting on sex and gender | This study does not involve human participants, their data, or their biological material. |
| Reporting on race, ethnicity, or other socially relevant groupings | This study does not involve human participants, their data, or their biological material. |
| Population characteristics | This study does not involve human participants, their data, or their biological material. |
| Recruitment | This study does not involve human participants, their data, or their biological material. |
| Ethics oversight | This study does not involve human participants, their data, or their biological material. |

Note that full information on the approval of the study protocol must also be provided in the manuscript.

# Field-specific reporting

Please select the one below that is the best fit for your research. If you are not sure, read the appropriate sections before making your selection.

☒ Life sciences ☐ Behavioural & social sciences ☐ Ecological, evolutionary & environmental sciences

For a reference copy of the document with all sections, see nature.com/documents/nr-reporting-summary-flat.pdf

# Life sciences study design

All studies must disclose on these points even when the disclosure is negative.

| | |
|---|---|
| Sample size | The sample size for each quantitative experiment was at least n = 3. n represents independent well replicates, randomly selected microscopy fields within a well, individual mice, or independent assay replicates derived from a cell lysate or purified protein. Replicates in in vitro assays were treated as independent samples. Sample sizes were determined based on previously published work, experimental and technical constraints, and sample availability. |
| Data exclusions | In general, data were not excluded, and individual data points are shown in most figures. For in vivo tracer studies, measurements below the detection limit were classified as not detectable (n.d.). |
| Replication | To confirm the results, most experiments were independently replicated 2-3 times. Number of replications are reported in figure legends. A replication refers to an independent repeat of the experiment, not a re-measurement/analysis of the same experimental sample. |
| Randomization | Randomization was not applicable for this study. Samples were allocated to experimental groups based on predefined genotype and/or treatment condition. Potential sources of variation were minimized by processing experimental groups in parallel under the same conditoins, using cells of matched passage number, and using age- and sex-matched mice where applicable. |
| Blinding | Most experiments did not involve blinding. Regardless of experimental group, all samples were processed and handled identically according to standardized protocols. Microscopy data were acquired from randomly allocated areas of the specimen. Imaging experiments were confirmed by a blinded investigator. |

# Reporting for specific materials, systems and methods

We require information from authors about some types of materials, experimental systems and methods used in many studies. Here, indicate whether each material, system or method listed is relevant to your study. If you are not sure if a list item applies to your research, read the appropriate section before selecting a response.

## Materials & experimental systems

| n/a | Involved in the study |
| --- | --- |
| ☐ | ☒ Antibodies |
| ☐ | ☒ Eukaryotic cell lines |
| ☒ | ☐ Palaeontology and archaeology |
| ☐ | ☒ Animals and other organisms |
| ☒ | ☐ Clinical data |
| ☒ | ☐ Dual use research of concern |
| ☒ | ☐ Plants |

## Methods

| n/a | Involved in the study |
| --- | --- |
| ☒ | ☐ ChIP-seq |
| ☒ | ☐ Flow cytometry |
| ☒ | ☐ MRI-based neuroimaging |

# Antibodies

Antibodies used

Anti-6X His tag® antibody; Abcam; Cat. Nr.: ab18184; Clone: HIS.H8
Anti-HA antibody; Sigma-Aldrich; Cat. Nr.:H3663; Clone: HA-7
Rabbit Anti-GAPDH Monoclonal Antibody; Cell Signaling Technology; Cat. Nr.: 2118; Clone: 14C10
PDI Rabbit mAb; Cell Signaling Technology; Cat. Nr.: 3501S; Clone: C81H6
LAMP1 Rabbit mAb; Cell Signaling Technology; Cat. Nr.: 3243; Clone: C54H11
ß-Actin Antibody; Cell Signaling Technology; Cat. Nr.: 4967
Anti-SDHA antibody; Abcam; Cat. Nr.: ab14715; Clone: 2E3GC12FB2AE2
Calnexin (C5C9) Rabbit mAB; Cell Signaling Technology; Cat.Nr.: 2679; Clone: C5C9
Goat Anti-Rabbit IgG Antibody (H+L), Peroxidase; Vector Laboratories; Cat. Nr.: PI-1000-1
Sheep Anti-Mouse IgG ECL Antibody, HRP Conjugated; Cytiva; Cat. Nr.: NA9310-1ml

Validation

All antibodies used for immunoblotting have been previously reported in the literature and are validated by the manufacturer. For detailed information, refer to the manufacturers' websites using the product names and catalog numbers listed above.

# Eukaryotic cell lines

Policy information about cell lines and Sex and Gender in Research

Cell line source(s)

Hek292 (CRL-1573): American Type Culture Collection (ATCC); Hap1 (C631): Horizon; Expi293F (A14527): Thermo Fischer Scientific

Authentication

The authenticity of the cell lines is guaranteed by the sources listed above. Cell lines are checked for contamination and are verified by with STR profiling.

Mycoplasma contamination

All cell lines were regularly tested negative for mycoplasma contamination.

Commonly misidentified lines
(See ICLAC register)

No misidentified cell lines were used in this study

# Animals and other research organisms

Policy information about studies involving animals; ARRIVE guidelines recommended for reporting animal research, and Sex and Gender in Research

Laboratory animals

Cln8 mnd mice (B6.KB2-Cln8mnd/MsrJ; JAX strain number 001612), were maintained in a SOPF facility on a regular light-dark cycle (12 h light, 12 h dark) at a room temperature (RT 23±1°C) and fed ad libitum on a standard laboratory chow diet (SAFE 105; Rosenburg, Germany). Experiments were performed with animals aged 10 weeks.

Wild animals

The study did not involve wild animals.

Reporting on sex

At the time of the study, female mice were available for the experiment. Due to a similar phenotype in both sexes, it is expected that experiments on male mice would yield comparable results. The sex is specified in the "Methods" section of the manuscript.

Field-collected samples

The study did not involve samples collected from the field.

Ethics oversight

Experimental procedures were approved by the University of Cambridge Animal Welfare and Ethics Board and UK Home Office (PPL number PP1740969) and were conducted in accordance with the EU Directive 2010/63. All animal procedures were guided by the 3R's principles to minimize pain, suffering, and distress. All studies involving animals are reported in accordance with the ARRIVE guidelines.

Note that full information on the approval of the study protocol must also be provided in the manuscript.

## Plants

| | |
|---|---|
| Seed stocks | This study does not involve plants. |
| Novel plant genotypes | This study does not involve plants. |
| Authentication | This study does not involve plants. |
