# Supplementary material for: Batten disease protein CLN8 enables a non-canonical phospholipid synthesis pathway
Source: Nat Cell Biol. 2026 Sep 15;28(10):2078–87. doi: 10.1038/s41556-026-02059-8 (PMC13661288; doi:10.1038/s41556-026-02059-8)
Supplement: Supplementary file 4 [file 41556_2026_2059_MOESM3_ESM.pdf]

nature portfolio

Peer Review File

Batten disease protein CLN8 enables a non-canonical phospholipid synthesis pathway

Corresponding Author: Professor Robert Zimmermann

Version 0:

Decision Letter:

*Please delete the link to your author homepage if you wish to forward this email to co-authors.

Dear Dr Zimmermann,

Thank you again for submitting your manuscript, "CLN8 enables a non-canonical phospholipid synthesis pathway", to Nature Cell Biology. We sincerely apologize for the delay in sharing our decision with you. Your manuscript has now been seen by 3 referees, who are experts in lipid metabolism (Referee #1); LSDs (Referee #2); lipidomics, lipid biology (Referee #3). As you will see from their comments (attached below), they found the work of potential interest but have raised substantial concerns, which in our view would need to be addressed with considerable revisions before we can consider publication in Nature Cell Biology.

Nature Cell Biology editors discuss the referee reports in detail within the editorial team, including the chief editor, to identify key referee points that should be addressed with priority, and requests that are overruled as being beyond the scope of the current study. To guide the scope of the revisions, I have listed these points below. Our standard revision period is six months, and we are committed to providing a fair and constructive peer-review process, so please feel free to contact me if you anticipate issues or delays addressing the reviews.

I should stress that the referees' concerns point to a premature dataset and their concerns would need to be addressed thoroughly experimentally; reconsideration of the study for this journal and re-engagement of referees will depend on the strength of the revisions. In particular, it would be essential to address the reviews as follows:

-- Rev#1 and Rev#3's concerns about the mass spectrometry and tracer experiments should be addressed thoroughly and in full.

-- Please address all reviewers' comments about the supplementation experiments as guided by the reviewers' remarks.

-- Please add discussion about the use of disease-relevant cellular models (Rev#3's last point), BMP synthesis pathways, and issues related to ER-to-lysosome lipid trafficking.

-- All other referee concerns pertaining to strengthening existing data, providing controls, methodological details, clarifications and textual changes, should also be addressed.

-- Finally, please pay close attention to our guidelines on statistical and methodological reporting (listed below) as failure to do so may delay the reconsideration of the revised manuscript. In particular, please provide:

- a Supplementary Figure including unprocessed images of all gels/blots in the form of a multi-page pdf file. Please ensure that blots/gels are labeled and the sections presented in the figures are clearly indicated.

- a Supplementary Table including all numerical source data in Excel format, with data for different figures provided as different sheets within a single Excel file. The file should include source data giving rise to graphical representations and statistical descriptions in the paper and for all instances where the figures present representative experiments of multiple independent repeats, the source data of all repeats should be provided.

We would be happy to consider a revised manuscript that would satisfactorily address these points, unless a similar paper is published elsewhere or is accepted for publication in Nature Cell Biology in the meantime.

When revising the manuscript please:

- ensure that it conforms to our format instructions and publication policies (see below and https://www.nature.com/nature/for-authors).

- provide a point-by-point rebuttal to the full referee reports verbatim, as provided at the end of this letter.

- for any revision that includes light microscopy data, we ask our authors to please include a completed light microscopy reporting table https://www.nature.com/documents/Light_microscopy_reporting_table.xlsx to ensure the methods are described thoroughly. The table will be available to reviewers and ultimately published should the manuscript be accepted at the journal.

- provide the completed Reporting Summary (found here https://www.nature.com/documents/nr-reporting-summary.pdf). This is essential for reconsideration of the manuscript will be available to editors and referees in the event of peer review. For more information see http://www.nature.com/authors/policies/availability.html or contact me.

When submitting the revised version of your manuscript, please pay close attention to our href="https://www.nature.com/nature-portfolio/editorial-policies/image-integrity">Digital Image Integrity Guidelines. and to the following points below:

-- that unprocessed scans are clearly labelled and match the gels and western blots presented in figures.
-- that control panels for gels and western blots are appropriately described as loading on sample processing controls
-- all images in the paper are checked for duplication of panels and for splicing of gel lanes.

Finally, please ensure that you retain unprocessed data and metadata files after publication, ideally archiving data in perpetuity, as these may be requested during the peer review and production process or after publication if any issues arise.

Nature Cell Biology is committed to improving transparency in authorship. As part of our efforts in this direction, we are now requesting that all authors identified as 'corresponding author' on published papers create and link their Open Researcher and Contributor Identifier (ORCID) with their account on the Manuscript Tracking System (MTS), prior to acceptance. ORCID helps the scientific community achieve unambiguous attribution of all scholarly contributions. You can create and link your ORCID from the home page of the MTS by clicking on 'Modify my Springer Nature account'. For more information please visit please visit www.springernature.com/orcid.

This journal strongly supports public availability of data. Please place the data used in your paper into a public data repository, or alternatively, present the data as Supplementary Information. If data can only be shared on request, please explain why in your Data Availability Statement, and also in the correspondence with your editor. Please note that for some data types, deposition in a public repository is mandatory - more information on our data deposition policies and available repositories appears below.

Please submit the revised manuscript files and the point-by-point rebuttal to the referee comments using this link:

Link Redacted

*This url links to your confidential home page and associated information about manuscripts you may have submitted or be reviewing for us. If you wish to forward this email to co-authors, please delete the link to your homepage.

We hope that you will find our referees' comments and editorial guidance helpful. Please do not hesitate to contact me if there is anything you would like to discuss. Thank you again for considering NCB for your work.

Best wishes,

Melina

Melina Casadio, PhD
Senior Editor, Nature Cell Biology
ORCID ID: https://orcid.org/0000-0003-2389-2243

Reviewers' Comments:

Reviewer #1 (Remarks to the Author):

In this manuscript, Johannes Breithofer et al. identify CLN8 as a glycerophosphoglycerol (GPG) acyltransferase (GPGAT)

that mediates the stereospecific synthesis of lyso-phosphatidylglycerol (LPG)—a critical precursor for the CLN5-based transacylase synthesis of bis(monoacylglycero)phosphate (BMP) in mammalian cells. The authors present striking evidence for a profound reduction in BMP levels in CLN8-deficient cells, which they link to the development of phospholipidosis. This suggests that BMP deficiency may be a primary driver of lipid storage disorders, providing a compelling mechanistic explanation for CLN8-associated Batten disease.

The GPGAT activity was clearly validated across multiple mouse tissues, including the brain, liver, and kidney, using both in vitro and in vivo models. The methodological integration of isotope tracing, CRISPR-mediated gene knockout, and comprehensive lipidomic analyses addresses a long-standing gap in our understanding of BMP metabolism. Specifically, it has been argued that BMP is synthesized from PG (a precursor primarily synthesized within mitochondria), and this study elucidates a GPG acylation pathway mediated by the endoplasmic reticulum (ER) enzyme CLN8. This discovery addresses the questions regarding the major biosynthetic precursor and in part, the spatial constraints of BMP biosynthesis. Elucidation of this pathway is highly significant and highlights potential therapeutic targets for Batten disease.

However, I have major concerns regarding the mass spectrometry-based methods used for the detection and quantification of BMP and PG in this study, as detailed below. Given that BMP and PG are structural isomers, the lack of rigorous differentiation significantly undermines the reliability of the key conclusions. I hope the authors can provide further evidence to validate their findings and improve the credibility of the data. This is critical not only for the integrity of the major conclusions in this study but also for the broader research community interested in BMP biology. Accurate detection and quantitative analysis of BMP—specifically distinguishing it from its isomers—is a non-trivial task that requires standardized procedures and rigorous validation to ensure data accuracy.

Major concerns:
1. Lack of Specificity in BMP and PG MS Detection
BMP and PG are structural isomers with identical mass-to-charge (m/z) ratios; therefore, their unambiguous distinction depends entirely on both chromatographic resolution and the selection of unique MS transitions. The manuscript fails to demonstrate that the chosen multiple reaction monitoring (MRM) channels for BMP and PG are non-overlapping. On the contrary, Supplementary Table 4 appears to show identical MRMs channels for BMP and PG. Therefore, there is no evidence ensuring that the product ions selected for BMP do not cross-react with PG (or vice versa) if they elute at similar retention times (RTs).

Furthermore, the described LC methods appear insufficient. The method uses a BEH C18 column starting at 50% B (high organic content). Under these conditions, polar isomers like PG and BMP would likely exhibit minimal retention, making their baseline separation highly improbable. If peak annotation relies on RT, the authors must provide explicit RT windows for both BMP and PG standards and explain the logic used to identify endogenous species. Without this rigorous validation, it is impossible to rule out the misassignment of PG signals to BMP, which directly undermines the core conclusions regarding the selective synthesis of BMP.

The authors must provide (i) MRM transition specificity data (e.g., spiking experiments with pure standards to demonstrate no cross-reactivity); (ii) representative chromatograms of BMP and PG standards with clearly annotated RTs; and (iii) overlay chromatograms of biological samples spiked with standards to confirm that endogenous species match the RTs of the standards.

2. Inadequate Validation of Isotope-Labeled BMP/PG Distinction
Isotope tracer experiments (e.g., [13C]GPG incorporation) rely heavily on distinguishing labeled BMP ([13C]BMP) from labeled PG ([13C]PG). Currently, there is no data to confirm that the m/z shifts used to identify [13C]BMP (+3, +5) do not overlap with [13C]PG isotopologues. For example, a [13C]PG species with a single labeled glycerol moiety could potentially share an m/z with a [13C]BMP species, leading to misquantification. Additionally, there is no evidence that labeled BMP and PG elute at distinct RTs to avoid co-detection.

The authors must supplement the manuscript with (i) isotope distribution calculations demonstrating that [13C]BMP and [13C]PG isotopologues have non-overlapping m/z ranges under the experimental conditions; (ii) RT profiles of labeled BMP/PG standards (e.g., [13C]GPG-derived BMP vs. [13C]glycerol-derived PG).

Minor Points:
1. Please elaborate on the metabolic source of GPG. Additionally, provide some discussion on the transport mechanism of ER-synthesized LPG to the lysosome for BMP synthesis.
2. Fig. 2j,k: Please clarify the experimental setup (e.g., were purified enzymes utilized?). The LPG signal appears low relative to background noise; representative ion chromatograms (EICs) should be provided, accompanied by a detailed description of the peak annotation logic.
3. In the relevant figures, please indicate the stereochemical configuration (R or S) for each chiral carbon.
4. Fig. 1m,n: Triacsin C treatment reduces the incorporation of newly synthesized [13C]-LPG and [13C]-BMP, yet while total BMP levels decrease, total LPG levels remain largely unchanged. Please explain.
5. Cardiolipin is not usually quantitatively measured using tri-quad mass spectrometers. Please provide evidence supporting the method validity.
6. Please correct typographical errors in the supplementary materials; for example, Supplementary Table 4 is currently listed as "Neagtive."

Reviewer #1 (Remarks on Protocol(s)):

Please refer to my related comments in the general comments section.

Reviewer #2 (Remarks to the Author):

Breithofer et al. have conducted a thorough study of the biosynthesis of BMP and the involvement of CLN8 therein (and, to a lesser extent, the involvement of CLN5). The authors examined these processes in a range of preparations, including "lysates" (1000 x g supernatants of homogenized (cultured) cells and mouse tissues), sonicated membranes of cultured cells, live cells (unmodified, transfected, KO), live mice, as well as purified CLN8. This study also included a significant organic synthesis effort. Altogether, the authors provide convincing data for CLN8 catalyzing an essential step in the synthesis of BMP, acylating GPG in the ER, producing LPG that is transported to lysosomes, where it is converted to BMP by CLN5. CLN8 selectively acylates the S-linked glycerol moiety of GPG, and is essential for the synthesis of endogenous 2,2'-BMP. In addition, the authors show that BMP supplementation rescues the phospholipidosis of CLN8-deficient cells; the exogenous sn-3,3'-S,S-BMP is in part converted to sn-2,2'-S,S-BMP, albeit less efficiently in CLN8-KO cells than in WT cells.

Comments

[1] In the Main section (p.2), the first paragraph mentions "luminal membranes" twice. The term "intralumenal vesicles" better reflects the structure of these entities.

[2] In the first paragraph of the Results section (p.3), the second sentence talks about GPD degradation, which is described as something that is "currently thought". However, the review that the authors refer to here is from 2014. An actual recent review on this topic is that of Hejazian et al. (https://doi.org/10.1007/s10930-024-10190-4).

[3] In multiple figures (e.g. Fig 1, Fig 2 j, Extended data Fig. 3, Extended data Fig. 7) some of the lettering is so small that it will be challenging for readers to decipher without considerably zooming in. Even if the journal allows this, the very small fonts do the readers a disservice. The challenge for the authors is to present their data in an accessible graphic fashion – without having to resort to microscopic lettering.

[4A] On p. 10, the third paragraph poorly connects with the preceding paragraphs. It is preferable to change the order of this sentence, for example "In the rescue experiments we added sn-3,3'-S,S-BMP to CLN8-KO cells, which prevented phospholipidosis (Fig. 3g-j). It is remarkable that 3,3'-BMP rescued the pathology of CLN8-KO cells, considering that 2,2'-BMP is the native form of BMP. Recent studies have shown …".
[4B] This paragraph continues with comparing the levels of the different forms of BMP in BMP-treated WT and CLN8-KO cells, stating that the treated KO cells had 60% less 2,2'-BMP than treated WT cells. The paragraph concludes by stating that supplementing the KO cells with 3,3'-BMP – "nevertheless" – rescued them from phospholipidosis, as if this is something not fully understood. Curiously, the authors do not point out that the 3,3'-BMP-treated KO cells had a lot more 2,2'-BMP relative to untreated KO cells, as they had no 2,2'-BMP at all (Fig. 3 l and m). In fact, the level of 2,2'-BMP in sn-3,3'-S,S-BMP-supplemented KO cells was higher than that of untreated WT cells (Fig. 3 l and m). Is it not plausible that this physiological level of 2,2'-BMP in the KO cells was sufficient to stave off phospholipidosis?

[5] In the last paragraph of p. 10, results are presented of supplementing WT and CLN8-KO cells with commercially available R, rac LPG. In the treated KO cells, neither 2,2'-BMP nor 3,3'-BMP were detected. By contrast, the R, rac LPG supplementation restored 2,3'-BMP in KO cells to a similar level as that of untreated WT cells (Extended data Fig. 5). Different from the BMP supplementation experiments discussed ahead of this paragraph, no information is provided on the phenotype of the R, rac LPG-supplemented CLN8-KO cells. Did this treatment reduce the extent of phospholipidosis? If not, it is more plausible that the restoration of 2,2'-BMP by supplementation with 3,3'-BMP (Fig. 3 l and m) rescued the KO cells from phospholipidosis.

[6] Further to these supplementation experiments (p. 10), it is curious that the WT and CLN8-KO cells were cultured in the presence of 3,3'-BMP for 72-96 hours (Fig. 3 h and k), while the cells were cultured in the presence of R, rac LPG for only 18 hours (Extended data Fig. 5). This of course makes one wonder what the outcome of a 96-hour supplementation with R, rac LPG could be. What made was the reason for limiting the R, rac LPG supplementation to 18 hours?

[7] The last sentence on p. 10 does not require "respectively". How about rewriting part of this sentence as follows: "Notably, exogenous LPG raised the GPG content of WT and of CLN8-KO cells 80-fold, suggesting that …".

[8] Page 12, the first paragraph of the "CLN8 acylates GPG in vitro and in vivo" section. This paragraph deals with loading live cells with [13C]GPG. For continuity of the paper, it may be good to remind the reader here of the findings of Figs. 1 d and h, where similar experiments were done, albeit with only WT HEK293 cells.

[9] Page 12, the second paragraph of the "CLN8 acylates GPG in vitro and in vivo" section. Here an earlier experiment with BafA1 (Fig. 1 p) is revisited, in one brief sentence, which is somewhat jarring. It would be good to describe the earlier experiments more extensively, to bring the reader up to speed without having to interrupt their reading of this section by going back to p. 4. Only after a more extensive reminder, the new experiments with BafA1 can be described (Fig. 4 d).

[10] In the final paragraph of the Discussion (p. 15), the authors briefly discuss the study of Singh et al. (https://doi.org/10.1016/j.cell.2024.09.036, reference 10). This study was reviewed by experts in BMP metabolism, in the Annual Review of Biochemistry (https://doi.org/10.1146/annurev-biochem-092823-113814). Singh et al. proposed a distinct metabolic pathway for BMP synthesis, requiring lysosomal phospholipases D3/D4 (PLD3/4). The authors of the current manuscript need to significantly expand their discussion of the paper by Singh et al., comparing and contrasting it with their own work. It is important that the authors point out in some detail how the approaches of Singh et al. led to insights that are fundamentally different from those of the current manuscript. Alternatively, the authors could provide a synthesis of both studies, showing how the roles of PLD3/4 and CLN8 may be complementary, following the wider scope of the Discussion of Bulfon et al. (https://doi.org/10.1038/s41467-024-54213-1). With the current knowledge, would it be is possible to arrive at a unified vision of BMP synthesis, or are there outstanding issues to be resolved experimentally?

Reviewer #2 (Remarks on Protocol(s)):

Methods have been adequately described.

Reviewer #3 (Remarks to the Author):

In this manuscript, the authors identify CLN8 as a glycerophosphoglycerol (GPG) acyltransferase that initiates a previously unrecognized, non-canonical pathway for bis(monoacylglycero)phosphate (BMP) synthesis. Through a combination of isotope tracing, biochemical reconstitution, genetic loss-of-function models, and in vivo mouse studies, the authors demonstrate that CLN8 catalyzes the stereospecific acylation of GPG to generate lysophosphatidylglycerol, which is selectively routed toward BMP synthesis in lysosomes. Loss of CLN8 results in BMP deficiency, lysosomal phospholipid accumulation, and phospholipidosis that can be partially rescued by BMP supplementation.

Overall, this study provides a compelling biochemical explanation linking CLN8 function to lysosomal lipid homeostasis and offers a mechanistic framework for understanding lysosomal storage defects in CLN8-associated Batten disease. Conceptually, the work adds to canonical models of phospholipid biosynthesis by demonstrating that a glycerophosphodiester, traditionally viewed as a degradation intermediate, can be directly reutilized for lipid synthesis in mammalian cells.

Comments/Suggestions:

1. While the study convincingly demonstrates that mammalian cells can reutilize GPG for BMP synthesis, the manuscript provides limited insight into how frequently or under what physiological conditions this pathway contributes meaningfully to BMP pools. GPG is generally considered a transient degradation intermediate rapidly cleared by glycerophosphodiesterases. The elevated GPG levels observed in CLN8-deficient cells may reflect pathological accumulation rather than normal metabolic flux. Thus, it remains unclear whether the CLN8-dependent pathway is a dominant contributor to BMP synthesis under basal conditions or becomes particularly important during periods of elevated membrane turnover, lysosomal stress, or disease.

2. A significant portion of the study relies on isotope-tracing experiments using relatively high concentrations of exogenous GPG (typically 250–500 µM in cells and ~55 mg/kg in vivo). While these concentrations facilitate robust detection of labeled lipids, they likely exceed physiological intracellular GPG levels. At such concentrations, tracer-driven effects may bias pathway usage, potentially exaggerating the apparent importance or selectivity of CLN8-mediated GPG acylation.

3. The GPG tracers used are racemic mixtures. While the authors address stereochemical specificity mechanistically, the endogenous stereochemical composition of cellular GPG pools is not established. As a result, extrapolation from tracer behavior to native metabolism remains somewhat uncertain, particularly when drawing conclusions about stereoselective routing under physiological conditions. This limitation should be discussed more explicitly.

4. Most tracer experiments are conducted over 16/18 hour incubations, a duration that allows for secondary lipid remodeling, recycling, and compensatory responses. These long incubations reduce temporal resolution and make it difficult to distinguish primary, direct metabolic events from downstream consequences of prolonged tracer exposure.

5. The proposed model requires selective delivery of CLN8-generated LPG from the ER to lysosomes, yet no direct trafficking mechanism is demonstrated. The absence of identifiable lipid transfer proteins, vesicular intermediates, or ER–lysosome contact site involvement leaves an important gap in the mechanistic narrative. While this may be beyond the scope of the current study, more explicit acknowledgment of this unresolved step would strengthen the overall model.

6. The lipid species proposed in Figure 1 and 4 appear to be assigned primarily based on m/z shifts following isotope labeling. While this approach is informative for tracking incorporation, it is insufficient on its own for unambiguous lipid identification. MS/MS-based structural validation is necessary to confirm lipid class and acyl-chain positioning. It is possible that such analyses were performed, but this is not clearly described or presented in the manuscript and should be clarified.

7. The analysis in Figure 2 relies on differential cell lysate fractions that are presumed to be enriched for distinct subcellular compartments. However, comprehensive characterization of these fractions is not shown. Inclusion of marker-based validation would be important to confirm that the fractions correspond to the intended subcellular structures and to support

the conclusions drawn from these experiments.

8. Clarification needed regarding BMP supplementation experiments: The rescue experiments utilize a non-endogenous BMP species. Although this distinction is noted, it remains unclear how much of the observed positional isomer distribution reflects physiological BMP remodeling versus cellular handling of an exogenously supplied lipid.

9. The in vivo tracer experiments fail to demonstrate BMP synthesis in brain tissue despite the neurological focus of the disease. Without further discussion, it remains unclear whether this reflects technical limitations of tracer delivery or fundamental differences in BMP metabolism in the brain.

10. Most mechanistic conclusions are derived from HEK293 and Hap1 cells, which may not fully capture the lipid metabolic context relevant to neurodegeneration. Given the otherwise high level of rigor and depth in the study, the limited use of more disease-relevant cellular models stands out and warrants discussion.

GUIDELINES FOR MANUSCRIPT SUBMISSION TO NATURE CELL BIOLOGY

READABILITY OF MANUSCRIPTS – Nature Cell Biology is read by cell biologists from diverse backgrounds, many of whom are not native English speakers. Authors should aim to communicate their findings clearly, explaining technical jargon that might be unfamiliar to non-specialists, and avoiding non-standard abbreviations. Titles and abstracts should concisely communicate the main findings of the study, and the background, rationale, results and conclusions should be clearly explained in the manuscript in a manner accessible to a broad cell biology audience. Nature Cell Biology uses British spelling.

MANUSCRIPT FORMAT – please follow the guidelines listed in our Guide to Authors regarding manuscript formats at Nature Cell Biology.

TITLE – should be no more than 100 characters including spaces, without punctuation and avoiding technical terms, abbreviations, and active verbs..

AUTHOR NAMES – should be given in full.

AUTHOR AFFILIATIONS – should be denoted with numerical superscripts (not symbols) preceding the names. Full addresses should be included, with US states in full and providing zip/post codes. The corresponding author is denoted by: "Correspondence should be addressed to [initials]."

ABSTRACT AND MAIN TEXT – please follow the guidelines that are specific to the format of your manuscript, as listed in our Guide to Authors (http://www.nature.com/ncb/pdf/ncb_gta.pdf) Briefly, Nature Cell Biology Articles, Resources and Technical Reports have 3500 words, including a 150 word abstract, and the main text is subdivided in Introduction, Results, and Discussion sections. Nature Cell Biology Letters have up to 2500 words, including a 180 word introductory paragraph (abstract), and the text is not subdivided in sections.

ACKNOWLEDGEMENTS – should be kept brief. Professional titles and affiliations are unnecessary. Grant numbers can be listed.

AUTHOR CONTRIBUTIONS – must be included after the Acknowledgements, detailing the contributions of each author to the paper (e.g. experimental work, project planning, data analysis etc.). Each author should be listed by his/her initials.

FINANCIAL AND NON-FINANCIAL COMPETING INTERESTS – the authors must include one of three declarations: (1) that they have no financial and non-financial competing interests; (2) that they have financial and non-financial competing interests; or (3) that they decline to respond, after the Author Contributions section. This statement will be published with the article, and in cases where financial and non-financial competing interests are declared, these will be itemized in a web supplement to the article. For further details please see https://www.nature.com/licenceforms/nrg/competing-interests.pdf.

REFERENCES – are limited to a total of 70 for Articles, Resources, Technical Reports; and 40 for Letters. This includes references in the main text and Methods combined. References must be numbered sequentially as they appear in the main text, tables and figure legends and Methods and must follow the precise style of Nature Cell Biology references. References only cited in the Methods should be numbered consecutively following the last reference cited in the main text. References only associated with Supplementary Information (e.g. in supplementary legends) do not count toward the total reference limit and do not need to be cited in numerical continuity with references in the main text. Only published papers can be cited, and each publication cited should be included in the numbered reference list, which should include the manuscript titles. Footnotes are not permitted.

METHODS – Nature Cell Biology publishes methods online. The methods section should be provided as a separate Word document, which will be copyedited and appended to the manuscript PDF, and incorporated within the HTML format of the paper.

Methods should be written concisely, but should contain all elements necessary to allow interpretation and replication of the results. As a guideline, Methods sections typically do not exceed 3,000 words. The Methods should be divided into subsections listing reagents and techniques. When citing previous methods, accurate references should be provided and any alterations should be noted. Information must be provided about: antibody dilutions, company names, catalogue numbers and clone numbers for monoclonal antibodies; sequences of RNAi and cDNA probes/primers or company names and catalogue numbers if reagents are commercial; cell line names, sources and information on cell line identity and authentication. Animal studies and experiments involving human subjects must be reported in detail, identifying the committees approving the protocols. For studies involving human subjects/samples, a statement must be included confirming that informed consent was obtained. Statistical analyses and information on the reproducibility of experimental results should be provided in a section titled "Statistics and Reproducibility".

All Nature Cell Biology manuscripts submitted on or after March 21 2016 must include a Data availability statement as a separate section after Methods but before references, under the heading "Data Availability". . For Springer Nature policies on data availability see http://www.nature.com/authors/policies/availability.html; for more information on this particular policy see http://www.nature.com/authors/policies/data/data-availability-statements-data-citations.pdf. The Data availability statement should include:

• Accession codes for primary datasets (generated during the study under consideration and designated as "primary accessions") and secondary datasets (published datasets reanalysed during the study under consideration, designated as "referenced accessions"). For primary accessions data should be made public to coincide with publication of the manuscript. A list of data types for which submission to community-endorsed public repositories is mandated (including sequence, structure, microarray, deep sequencing data) can be found here http://www.nature.com/authors/policies/availability.html#data.

• Unique identifiers (accession codes, DOIs or other unique persistent identifier) and hyperlinks for datasets deposited in an approved repository, but for which data deposition is not mandated (see here for details http://www.nature.com/sdata/data-policies/repositories).

• At a minimum, please include a statement confirming that all relevant data are available from the authors, and/or are included with the manuscript (e.g. as source data or supplementary information), listing which data are included (e.g. by figure panels and data types) and mentioning any restrictions on availability.

• If a dataset has a Digital Object Identifier (DOI) as its unique identifier, we strongly encourage including this in the Reference list and citing the dataset in the Methods.

We recommend that you upload the step-by-step protocols used in this manuscript to protocols.io. More details can found at https://www.protocols.io/help/publish-articles.

DISPLAY ITEMS – main display items are limited to 6-8 main figures and/or main tables for Articles, Resources, Technical Reports; and 5 main figures and/or main tables for Letters. For Supplementary Information see below.

FIGURES – Colour figure publication costs $600 for the first, and $300 for each subsequent colour figure. All panels of a multi-panel figure must be logically connected and arranged as they would appear in the final version. Unnecessary figures and figure panels should be avoided (e.g. data presented in small tables could be stated briefly in the text instead).

All imaging data should be accompanied by scale bars, which should be defined in the legend.
Cropped images of gels/blots are acceptable, but need to be accompanied by size markers, and to retain visible background signal within the linear range (i.e. should not be saturated). The boundaries of panels with low background have to be demarked with black lines. Splicing of panels should only be considered if unavoidable, and must be clearly marked on the figure, and noted in the legend with a statement on whether the samples were obtained and processed simultaneously. Quantitative comparisons between samples on different gels/blots are discouraged; if this is unavoidable, it should only be performed for samples derived from the same experiment with gels/blots were processed in parallel, which needs to be stated in the legend.

Figures should be provided at approximately the size that they are to be printed at (single column is 86 mm, double column is 170 mm) and should not exceed an A4 page (8.5 x 11"). Reduction to the scale that will be used on the page is not necessary, but multi-panel figures should be sized so that the whole figure can be reduced by the same amount at the smallest size at which essential details in each panel are visible. In the interest of our colour-blind readers we ask that you avoid using red and green for contrast in figures. Replacing red with magenta and green with turquoise are two possible colour-safe alternatives. Lines with widths of less than 1 point should be avoided. Sans serif typefaces, such as Helvetica (preferred) or Arial should be used. All text that forms part of a figure should be rewritable and removable.

We accept files from the following graphics packages in either PC or Macintosh format:

- For line art, graphs, charts and schematics we prefer Adobe Illustrator (.AI), Encapsulated PostScript (.EPS) or Portable Document Format (.PDF). Files should be saved or exported as such directly from the application in which they were made, to allow us to restyle them according to our journal house style.

- We accept PowerPoint (.PPT) files if they are fully editable. However, please refrain from adding PowerPoint graphical effects to objects, as this results in them outputting poor quality raster art. Text used for PowerPoint figures should be Helvetica (preferred) or Arial.

- We do not recommend using Adobe Photoshop for designing figures, but we can accept Photoshop generated (.PSD or .TIFF) files only if each element included in the figure (text, labels, pictures, graphs, arrows and scale bars) are on separate layers. All text should be editable in 'type layers' and line-art such as graphs and other simple schematics should be preserved and embedded within 'vector smart objects' - not flattened raster/bitmap graphics.

- Some programs can generate Postscript by 'printing to file' (found in the Print dialogue). If using an application not listed above, save the file in PostScript format or email our Art Editor, Allen Beattie for advice (a.beattie@nature.com).

Regardless of format, all figures must be vector graphic compatible files, not supplied in a flattened raster/bitmap graphics format, but should be fully editable, allowing us to highlight/copy/paste all text and move individual parts of the figures (i.e. arrows, lines, x and y axes, graphs, tick marks, scale bars etc.). The only parts of the figure that should be in pixel raster/bitmap format are photographic images or 3D rendered graphics/complex technical illustrations.

All placed images (i.e. a photo incorporated into a figure) should be on a separate layer and independent from any superimposed scale bars or text. Individual photographic images must be a minimum of 300+ DPI (at actual size) or kept constant from the original picture acquisition and not decreased in resolution post image acquisition. All colour artwork should be RGB format.

EXTENDED DATA FIGURES - When re-submitting your manuscript, please ensure that any supplementary figures and tables that are crucial to the manuscript's conclusions are converted into Extended Data figures and tables to increase visibility of these data. Extended Data figures and tables are online-only (present in the online PDF and full-text HTML versions of the paper), peer-reviewed display items that provide essential background to the article but are not included in the main article due to space constraints. A maximum of ten Extended Data display items (figures and tables) is permitted.

FIGURE LEGENDS – must not exceed 350 words for each figure to allow fit on a single printed NCB page together with the figure. They must include a brief title for the whole figure, and short descriptions of each panel with definitions of the symbols used, but without detailing methodology.

TABLES – main tables should be provided as individual Word files, together with a brief title and legend. For supplementary tables see below.

SUPPLEMENTARY INFORMATION – Supplementary information is material directly relevant to the conclusion of a paper, but which cannot be included in the printed version in order to keep the manuscript concise and accessible to the general reader. Supplementary information is an integral part of a Nature Cell Biology publication and should be prepared and presented with as much care as the main display item, but it must not include non-essential data or text, which may be removed at the editor's discretion. All supplementary material is fully peer-reviewed and published online as part of the HTML version of the manuscript. Supplementary Figures and Supplementary Notes are appended at the end of the main PDF of the published manuscript.

Supplementary items should relate to a main text figure, wherever possible, and should be mentioned sequentially in the main manuscript, designated as Supplementary Figure, Table, Video, or Note, and numbered continuously (e.g. Supplementary Figure 1, Supplementary Figure 2, Supplementary Table 1, Supplementary Table 2 etc.).

Unprocessed scans of all key data generated through electrophoretic separation techniques need to be presented in a supplementary figure that should be labelled and numbered as the final supplementary figure, and should be mentioned in every relevant figure legend. This figure does not count towards the total number of figures and is the only figure that can be displayed over multiple pages, but should be provided as a single file, in PDF or TIFF format. Data in this figure can be displayed in a relatively informal style, but size markers and the figures panels corresponding to the presented data must be indicated.

The total number of Supplementary Figures (not including the "unprocessed scans" Supplementary Figure) should not exceed the number of main display items (figures and/or tables (see our Guide to Authors and March 2012 editorial http://www.nature.com/ncb/authors/submit/index.html#suppinfo; http://www.nature.com/ncb/journal/v14/n3/index.html#ed). No restrictions apply to Supplementary Tables or Videos, but we advise authors to be selective in including supplemental data.

Each Supplementary Figure should be provided as a single page and as an individual file in one of our accepted figure formats and should be presented according to our figure guidelines (see above). Supplementary Tables should be provided as individual Excel files. Supplementary Videos should be provided as .avi or .mov files up to 50 MB in size. Supplementary Figures, Tables and Videos much be accompanied by a separate Word document including titles and legends.

GUIDELINES FOR EXPERIMENTAL AND STATISTICAL REPORTING

REPORTING REQUIREMENTS – We are trying to improve the quality of methods and statistics reporting in our papers. To that end, we are now asking authors to complete a reporting summary that collects information on experimental design and reagents. The Reporting Summary can be found here https://www.nature.com/documents/nr-reporting-summary.pdf)If you would like to reference the guidance text as you complete the template, please access these flattened versions at http://www.nature.com/authors/policies/availability.html.

STATISTICS – Wherever statistics have been derived the legend needs to provide the n number (i.e. the sample size used to derive statistics) as a precise value (not a range), and define what this value represents. Error bars need to be defined in the legends (e.g. SD, SEM) together with a measure of centre (e.g. mean, median). Box plots need to be defined in terms of minima, maxima, centre, and percentiles. Ranges are more appropriate than standard errors for small data sets. Wherever statistical significance has been derived, precise p values need to be provided and the statistical test used needs to be stated in the legend. Statistics such as error bars must not be derived from $n<3$. For sample sizes of $n<5$ please plot the individual data points rather than providing bar graphs. Deriving statistics from technical replicate samples, rather than biological replicates is strongly discouraged. Wherever statistical significance has been derived, precise p values need to be provided and the statistical test stated in the legend.

Information on how many times each experiment was repeated independently with similar results needs to be provided in the legends and/or Methods for all experiments, and in particular wherever representative experiments are shown.

We strongly recommend the presentation of source data for graphical and statistical analyses as a separate Supplementary Table, and request that source data for all independent repeats are provided when representative experiments of multiple independent repeats, or averages of two independent experiments are presented. This supplementary table should be in Excel format, with data for different figures provided as different sheets within a single Excel file. It should be labelled and numbered as one of the supplementary tables, titled “Statistics Source Data”, and mentioned in all relevant figure legends.

--------- Please don't hesitate to contact NCB@nature.com should you have queries about any of the above requirements ---------

Version 1:

Decision Letter:

Our ref: NCB-A60925A

16th June 2026

Dear Dr. Zimmermann,

Thank you for submitting your revised manuscript "CLN8 enables a non-canonical phospholipid synthesis pathway" (NCB-A60925A). It has now been seen by the original referees and their comments are below. The reviewers find that the paper has improved in revision, and therefore we'll be happy in principle to publish it in Nature Cell Biology, pending minor revisions to comply with our editorial and formatting guidelines.

We are now performing detailed checks on your paper and will send you a checklist detailing our editorial and formatting requirements in a couple of weeks. Please do not upload the final materials and make any revisions until you receive this additional information from us.

Thank you again for your interest in Nature Cell Biology. Please do not hesitate to contact me if you have any questions.

Sincerely,

Melina Casadio, PhD
Senior Editor, Nature Cell Biology
ORCID ID: https://orcid.org/0000-0003-2389-2243

Reviewer #1 (Remarks to the Author):

The authors have fully addressed all of my previous concerns. I am satisfied with the revisions and gladly recommend the work for publication.

Reviewer #2 (Remarks to the Author):

I thank Zimmermann et al. for addressing all my concerns. I am particularly happy with the revision of the Discussion (my query [10]), where the authors compare their study with that of Singh et al.

Reviewer #3 (Remarks to the Author):

The authors have sufficiently addressed my comments and concerns; thank you.

INTERNAL USE ONLY AIP modules required: ["Life Science"]

Version 2:

Decision Letter:

Dear Dr Zimmermann,

I am pleased to inform you that your manuscript, "Batten disease protein CLN8 enables a non-canonical phospholipid synthesis pathway", has now been accepted for publication in Nature Cell Biology.

Thank you for sending us the final manuscript files to be processed for print and online production, and for returning the manuscript checklists and other forms. Your manuscript will now be passed to our production team who will be in contact with you if there are any questions with the production quality of supplied figures and text.

Over the next few weeks, your paper will be copyedited to ensure that it conforms to Nature Cell Biology style. Once your paper is typeset, you will receive an email with a link to choose the appropriate publishing options for your paper and our Author Services team will be in touch regarding any additional information that may be required.

After the grant of rights is completed, you will receive a link to your electronic proof via email with a request to make any corrections within 48 hours. If, when you receive your proof, you cannot meet this deadline, please inform us at rjsproduction@springernature.com immediately.

You will not receive your proofs until the publishing agreement has been received through our system.

Due to the importance of these deadlines, we ask that you please let us know now whether you will be difficult to contact over the next month. If this is the case, we ask you provide us with the contact information (email, phone and fax) of someone who will be able to check the proofs on your behalf, and who will be available to address any last-minute problems.

If you have any questions about our publishing options, costs, Open Access requirements, or our legal forms, please contact ASJournals@springernature.com

Once your paper has been scheduled for online publication, the Nature press office will be in touch to confirm the details. An online order form for reprints of your paper is available at https://www.nature.com/reprints/author-reprints.html. All co-authors, authors' institutions and authors' funding agencies can order reprints using the form appropriate to their geographical region.

Publication is conditional on the manuscript not being published elsewhere and on there being no announcement of this work to any media outlet until the online publication date in Nature Cell Biology.

You may wish to make your media relations office aware of your accepted publication, in case they consider it appropriate to organize some internal or external publicity. Once your paper has been scheduled you will receive an email confirming the publication details. This is normally 3-4 working days in advance of publication. If you need additional notice of the date and time of publication, please let the production team know when you receive the proof of your article to ensure there is sufficient time to coordinate. Further information on our embargo policies can be found here: https://www.nature.com/authors/policies/embargo.html

Please note that *Nature Cell Biology* is a Transformative Journal (TJ). Authors may publish their research with us through the traditional subscription access route or make their paper immediately open access through payment of an article-processing charge (APC). Authors will not be required to make a final decision about access to their article until it has been accepted. Find out more about Transformative Journals

Authors may need to take specific actions to achieve compliance with funder and institutional open access mandates. If your research is supported by a funder that requires immediate open access (e.g. according to Plan S principles or the <a

href=“https://www.springernature.com/gp/open-science/us-federal-agency-compliance”> NIH public access policy) then you should select the gold OA route, and we will direct you to the compliant route where possible. Because authors warrant under our subscription licensing terms that they haven’t committed to licensing any version of their article under a licence inconsistent with the terms of our agreement – including the applicable embargo period – publication under the subscription model isn’t suitable for authors whose funders require no embargo.

To assist our authors in disseminating their research to the broader community, our SharedIt initiative provides you with a unique shareable link that will allow anyone (with or without a subscription) to read the published article. Recipients of the link with a subscription will also be able to download and print the PDF.

If your paper includes color figures, please be aware that in order to help cover some of the additional cost of four-color reproduction, Nature Portfolio charges our authors a fee for the printing of their color figures. Please contact our offices for exact pricing and details.

As soon as your article is published, you will receive an automated email with your shareable link.

If you have not already done so, we strongly recommend that you upload the step-by-step protocols used in this manuscript to protocols.io (https://protocols.io), an open online resource that allows researchers to share their detailed experimental know-how. All uploaded protocols are made freely available and are assigned DOIs for ease of citation. Protocols and Nature Portfolio journal papers in which they are used can be linked to one another, and this link is clearly and prominently visible in the online versions of both. Authors who performed the specific experiments can act as primary authors for the Protocol as they will be best placed to share the methodology details, but the Corresponding Author of the present research paper should be included as one of the authors. By uploading your Protocols onto protocols.io, you are enabling researchers to more readily reproduce or adapt the methodology you use, as well as increasing the visibility of your protocols and papers. You can also establish a dedicated workspace to collect your lab Protocols. Further information can be found at https://www.protocols.io/help/publish-articles.

You can use a single sign-on for all your accounts, view the status of all your manuscript submissions and reviews, access usage statistics for your published articles and download a record of your refereeing activity for the Nature Portfolio.

Nature Cell Biology encourages authors presenting evidence for cell, biological, molecular, and genetic interactions to consider communicating these findings using Biofactoid (https://biofactoid.org/). This tool helps users share a searchable representation of interactions (e.g. binding, gene expression, post-translational modification) between genes, gene products, or chemicals. Information added to Biofactoid, with author attribution, is shared on social media and public databases, such as Pathway Commons, where it can be discovered and analyzed in the context of a large and growing corpus of knowledge.

Please feel free to contact us if you have any questions.

With kind regards,

Melina Casadio, PhD
Senior Editor, Nature Cell Biology
ORCID ID: https://orcid.org/0000-0003-2389-2243

Click here if you would like to recommend Nature Cell Biology to your librarian
http://www.nature.com/subscriptions/recommend.html#forms

** Visit the Springer Nature Editorial and Publishing website at www.springernature.com/editorial-and-publishing-jobs for more information about our career opportunities. If you have any questions please click here.**

Open Access This Peer Review File is licensed under a Creative Commons Attribution 4.0 International License, which permits use, sharing, adaptation, distribution and reproduction in any medium or format, as long as you give appropriate credit to the original author(s) and the source, provide a link to the Creative Commons license, and indicate if changes were made.
In cases where reviewers are anonymous, credit should be given to 'Anonymous Referee' and the source.
The images or other third party material in this Peer Review File are included in the article's Creative Commons license, unless indicated otherwise in a credit line to the material. If material is not included in the article's Creative Commons license and your intended use is not permitted by statutory regulation or exceeds the permitted use, you will need to obtain permission directly from the copyright holder.
To view a copy of this license, visit https://creativecommons.org/licenses/by/4.0/

Dear Reviewers,

We want to thank you for the careful evaluation and constructive feedback. We have carefully considered all comments and revised the manuscript accordingly. Below, we provide a detailed, point-by-point response to each comment (in bold) and indicate the corresponding revisions in the manuscript.

Response to reviewers

Reviewers' Comments:

<u>Reviewer #1 (Remarks to the Author):</u>

In this manuscript, Johannes Breithofer et al. identify CLN8 as a glycerophosphoglycerol (GPG) acyltransferase (GPGAT) that mediates the stereospecific synthesis of lyso-phosphatidylglycerol (LPG)—a critical precursor for the CLN5-based transacylase synthesis of bis(monoacylglycero)phosphate (BMP) in mammalian cells. The authors present striking evidence for a profound reduction in BMP levels in CLN8-deficient cells, which they link to the development of phospholipidosis. This suggests that BMP deficiency may be a primary driver of lipid storage disorders, providing a compelling mechanistic explanation for CLN8-associated Batten disease.

The GPGAT activity was clearly validated across multiple mouse tissues, including the brain, liver, and kidney, using both in vitro and in vivo models. The methodological integration of isotope tracing, CRISPR-mediated gene knockout, and comprehensive lipidomic analyses addresses a long-standing gap in our understanding of BMP metabolism. Specifically, it has been argued that BMP is synthesized from PG (a precursor primarily synthesized within mitochondria), and this study elucidates a GPG acylation pathway mediated by the endoplasmic reticulum (ER) enzyme CLN8. This discovery addresses the questions regarding the major biosynthetic precursor and in part, the spatial constraints of BMP biosynthesis. Elucidation of this pathway is highly significant and highlights potential therapeutic targets for Batten disease.

However, I have major concerns regarding the mass spectrometry-based methods used for the detection and quantification of BMP and PG in this study, as detailed below. Given that BMP and PG are structural isomers, the lack of rigorous differentiation significantly undermines the reliability of the key conclusions. I hope the authors can provide further evidence to validate their findings and improve the credibility of the data. This is critical not only for the integrity of the major conclusions in this study but also for the broader research community interested in BMP biology. Accurate detection and quantitative analysis of BMP—specifically distinguishing it from its isomers—is a non-trivial task that requires standardized procedures and rigorous validation to ensure data accuracy.

Major concerns:

1. Lack of Specificity in BMP and PG MS Detection

BMP and PG are structural isomers with identical mass-to-charge (m/z) ratios; therefore, their unambiguous distinction depends entirely on both chromatographic resolution and the selection of unique MS transitions. The manuscript fails to demonstrate that the chosen multiple reaction monitoring (MRM) channels for BMP and PG are non-overlapping. On the contrary, Supplementary Table 4 appears to show identical MRMs channels for BMP and PG. Therefore, there is no evidence ensuring that the product ions selected for BMP do not cross-react with PG (or vice versa) if they elute at similar retention times (RTs).

Furthermore, the described LC methods appear insufficient. The method uses a BEH C18 column starting at 50% B (high organic content). Under these conditions, polar isomers like PG and BMP would likely exhibit minimal retention, making their baseline separation highly improbable. If peak

annotation relies on RT, the authors must provide explicit RT windows for both BMP and PG standards and explain the logic used to identify endogenous species. Without this rigorous validation, it is impossible to rule out the misassignment of PG signals to BMP, which directly undermines the core conclusions regarding the selective synthesis of BMP.The authors must provide (i) MRM transition specificity data (e.g., spiking experiments with pure standards to demonstrate no cross-reactivity); (ii) representative chromatograms of BMP and PG standards with clearly annotated RTs; and (iii) overlay chromatograms of biological samples spiked with standards to confirm that endogenous species match the RTs of the standards.

Answer: The reviewer raised an important point about the specificity of our LC-MS based methods to quantify BMP and PG. We agree that, because BMP and PG are structural isomers with identical m/z ratios, unambiguous identification requires careful validation of the analytical procedures. Our MRM transitions were acquired in negative ion mode, selecting the deprotonated precursor ion ($[M–H]^-$) in the MS1 filter and fragmented acyl-chain ions in the MS2 filter. As the reviewer notes, these transitions by themselves are insufficient to distinguish BMP from PG isomers. Our chromatographic conditions were specifically optimized to provide sufficient retention and separation of BMP and PG despite the relatively high initial organic composition (50% B: 2-propanol/MeOH 8:2, v/v). Under the applied gradient, phospholipids elute in the middle section of the chromatogram with adequate retention. More polar lipid classes, such as lysophospholipids, are retained and elute in the early section of the chromatogram, whereas non-polar lipid classes (e.g., TAG and CE) elute later under increased organic conditions as shown in **Figure R1** (not part of the manuscript).

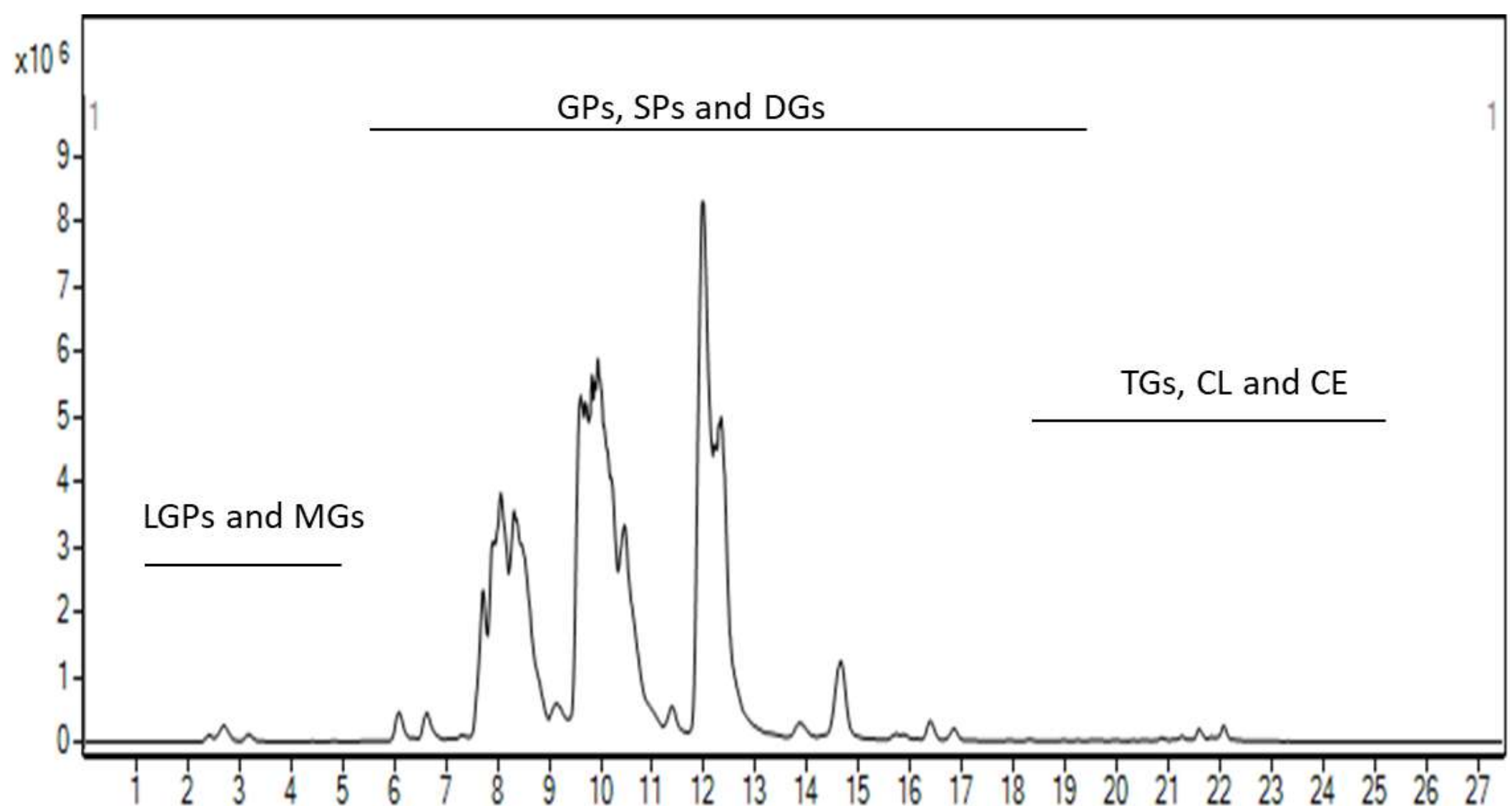

Figure R1. Representative total ion current (TIC) chromatogram of a brain lipid extract obtained using the described LC method. Distinct elution regions corresponding to lysoglycerophospholipids (LGPs), monoacylglycerols (MGs), glycerophospholipids (GPs), sphingolipids (SPLs), diacylglycerols (DGs), triacylglycerols (TGs), cardiolipin (CL) and cholesteryl esters (CEs) are indicated, demonstrating class dependent retention across a broad lipid polarity range.

As part of our method development, the chromatographic separation of BMP and PG was validated using lipid standards. Representative chromatograms for BMP (2,2 and 3,3 isomer) and PG standards with 18:1/18:1 acyl-chain compositions show clear separation under the applied gradient (**new Supplementary fig. 1a**). In addition, BMP peak identities were confirmed by MS-based structural validation: In the positive ion mode, a characteristic monoacylglycerol (MAG) fragment can be detected for BMP, while PG creates a characteristic diacylglycerol (DAG) fragment (**new Supplementary fig. 1a**). This fragmentation pattern enables assignment of the fatty acyl composition

of BMP and PG species. As these BMP- and PG-specific MAG and DAG fragments are detected with substantially lower sensitivity on our setup, these transitions were not used for quantification**.**

We further validated lipid assignments by spiking HEK293 lipid extracts with 18:1/18:1 BMP and 18:1/18:1 PG standards. These experiments confirmed the identities of endogenous BMP and PG peaks based on their distinct retention times and fragmentation behavior (**new Supplementary fig. 1a**).

Because BMP standards are commercially available only for 18:1/18:1 and 14:0/14:0 species, identification of retention times for other BMP and PG species was performed using complex mouse tissue lipid extracts to ensure coverage of a broad range of lipid species. These experiments were conducted in positive ion mode, where BMP generates characteristic MAG fragments, whereas PG produces DAG fragments.

Furthermore, BMP and PG species followed the expected chromatographic behavior in RP-HPLC according to the well-established equivalent carbon number (ECN) model, where ECN is defined as the total carbon number (CN) of fatty acyl chains minus twice the number of double bonds (DB) (ECN = CN − 2DB). Lipid retention increased proportionally with ECN, except for highly polyunsaturated species (≥4 double bonds), which exhibit slightly stronger retention. For example, BMP 22:6/22:6 (ECN = 20) and PC 20:4/20:4 (ECN = 24) elute within the ECN 28 retention group, consistent with established RP-HPLC lipid behavior.

Following the approach by Dong et al. in "Robust analytical methods for bis(monoacylglycero)phosphate profiling in health and disease" (bioRxiv, 2025), we used CLN5-KO cells as an additional control. This method leverages the markedly reduced BMP peak intensities observed in CLN5-KO cells as an additional criterion to distinguish BMP from PG peaks (**new Supplementary fig. 1b**). We calculated peak-intensity ratios of CLN5-KO cells (KO validated in Bulfon et al.; PMID: 39548099) relative to WT cells and confirmed that the ratios for the analyzed BMP species, but not for PG species, were consistently < 1, supporting correct BMP and PG assignment (**new Supplementary fig. 1b,c**).

We have now updated the MRM transition table with explicit retention times of all analyzed molecular lipid species (**new Supplementary table 4**).

2. Inadequate Validation of Isotope-Labeled BMP/PG Distinction
Isotope tracer experiments (e.g., [13C]GPG incorporation) rely heavily on distinguishing labeled BMP ([13C]BMP) from labeled PG ([13C]PG). Currently, there is no data to confirm that the m/z shifts used to identify [13C]BMP (+3, +5) do not overlap with [13C]PG isotopologues. For example, a [13C]PG species with a single labeled glycerol moiety could potentially share an m/z with a [13C]BMP species, leading to misquantification. Additionally, there is no evidence that labeled BMP and PG elute at distinct RTs to avoid co-detection.

The authors must supplement the manuscript with (i) isotope distribution calculations demonstrating that [13C]BMP and [13C]PG isotopologues have non-overlapping m/z ranges under the experimental conditions; (ii) RT profiles of labeled BMP/PG standards (e.g., [13C]GPG-derived BMP vs. [13C]glycerol-derived PG).

<u>Answer:</u> Reviewer 1 also raised concerns regarding the differentiation of isotope-labeled BMP and PG species. To address the reviewer`s concern, we conducted a systematic evaluation of our PG and BMP target list that excludes potential mis-quantification.

As demonstrated in **new Supplementary fig. 1a**, BMP and PG isomers are baseline-separated under our chromatographic conditions. Isotope-labeled BMP and PG species co-elute with their corresponding unlabeled lipids. Therefore, labeled BMP and PG species retain the same baseline chromatographic separation as their unlabeled parent lipids, excluding mis-quantification of BMP and PG isomers with identical acyl chain composition (e.g. BMP 18:1-18:1 and PG 18:1 18:1).

In theory, differences in acyl-chain composition could lead to situations where a BMP species with acyl chains X/Y elutes at a similar retention time as a PG species with a different composition (A/B). If such species share identical mass or isotope labeled mass shift, mis-quantification can occur. To systematically exclude this possibility, we first compiled all precursor masses of unlabeled m/z +0 as well as m/z +3 and m/z +5 labeled BMP and PG species included in our target list (**Table R1**). We then identified species with unique precursor masses (marked in green) and recurring precursor masses (not colored). Species analyzed for tracer incorporation are highlighted in bold. All potential overlaps were subsequently evaluated by comparing both retention times and MRM transitions. For all overlaps marked by grey arrows, mis-quantification can be excluded because the species differ in their MRM transitions and/or retention times.

Table R1: List of BMP and PG precursor masses species with m/z +0, m/z +3, and m/z +5 as well as the corresponding retention time (RT). Unique precursor masses are highlighted in green whereas precursor masses occurring more than once are not colored. Species analyzed for tracer incorporation are highlighted in **bold**.

Compound name	m/ +0	m/z+3	m/z +5	RT BMP	RT PG
**BMP/PG 32:0 (16:0-16:0)**	**721.5**	**724.5**	**726.5**	**6.2**	**7.8**
BMP/PG 32:1 (16:0-16:1)	719.5	722.5	724.5	5.2	8
BMP/PG 34:0 (16:0-18:0)	749.5	752.5	754.5	7.8	n.a.
**BMP/PG 34:1 (16:0-18:1)**	**747.5**	**750.5**	**752.5**	**6.4**	**8**
BMP/PG 34:2 (16:1-18:1)	745.5	748.5	750.5	n.a.	6.7
BMP/PG 34:2 (16:0-18:2)	745.5	748.5	750.5	5.5	7
**BMP/PG 36:1 (18:0-18:1)**	**775.5**	**778.5**	**780.5**	**8**	**10.1**
**BMP/PG 36:2 (18:1-18:1)**	**773.5**	**776.5**	**778.5**	**6.6**	**8.3**
**BMP/PG 36:3 (18:1-18:2)**	**771.5**	**774.5**	**776.5**	**5.7**	**7.2**
**BMP/PG 36:4 (18:2-18:2)**	**769.5**	**772.5**	**774.5**	**5**	**6.7**
BMP/PG 36:5 (18:2-18:3)	767.5	770.5	772.5	4.5	n.a.
BMP/PG 38:4 (18:0-20:4)	797.5	800.5	802.5	6.7	n.a.
**BMP/PG 38:5 (18:1-20:4)**	**795.5**	**798.5**	**800.5**	**5.6**	**7**
BMP/PG 38:5 (18:2-20:3)	795.5	798.5	800.5	5.5	n.a.
BMP/PG 38:6 (16:0-22:6)	793.5	796.5	798.5	5.3	n.a.
BMP/PG 38:6 (18:2-20:4)	793.5	796.5	798.5	4.9	n.a.
BMP/PG 38:7 (16.1-22:6)	791.5	794.5	796.5	4.5	n.a.
BMP/PG 40:5 (18:1-22:4)	823.5	826.5	828.5	6.7	n.a.
BMP/PG 40:6 (18:1-22:5)	821.5	824.5	826.5	5.9	n.a.
BMP/PG 40:6 (18:2-22:4)	821.5	824.5	826.5	5.8	n.a.
**BMP/PG 40:7 (18:1-22:6)**	**819.5**	**822.5**	**824.5**	**5.6**	**n.a.**
BMP/PG 40:7 (18:2-22.5)	819.5	822.5	824.5	5.4	n.a.
BMP/PG 40:8 (18:2-22:6)	817.5	820.5	822.5	4.9	n.a.
BMP/PG 40:9 (18:3-22:6)	815.5	818.5	820.5	4.4	n.a.
BMP/PG 42:10 (20:4-22:6)	841.5	844.5	846.5	4.6	n.a.
BMP/PG 42:9 (20:3-22:6)	843.5	846.5	848.5	4.8	n.a.
BMP/PG 44:10 (22:4-22:6)	869.5	872.5	874.5	5.6	n.a.
BMP/PG 44:11 (22:5-22:6)	867.5	870.5	872.5	4.9	n.a.
**BMP/PG 44:12 (22:6-22:6)**	**865.5**	**868.5**	**870.5**	4.6	n.a.

→ (grey) non overalpping MRM transitions and/or retention times
→ (red) overlap in MRM transition

Two theoretical overlaps were identified (red arrows) where mis-quantification cannot be formally excluded based solely on precursor mass**.** In the first case, m/z +5 labeled BMP/PG 38:5 (18:1/20:4) shares the same precursor mass as m/z +3 labeled BMP/PG 38:4 (18:0/20:4), and both transitions monitor the 20:4 acyl-chain fragment in MS2. However, PG 18:0/20:4 was not detected in our samples, making mis-quantification highly unlikely. We have clearly noted these exclusions in Table R1.

As suggested by the reviewer, **new Supplementary fig. 1d** now shows corresponding m/z +0 and m/z +3 chromatograms of the two most abundant BMP and PG species in control and tracer treated HEK293 cells. Following treatment of [^{13}C]GPG^{+3}, a specific increase in m/z +3 labeled BMP but not PG (red line) is detected. In contrast, treatment with [U-^{13}C] glycerol enables the detection of m/z +3 labeled PG species and to a lesser extent also m/z+3 labeled BMP.

Due to the natural isotope distribution, signals in the m/z +3 channel are detected to a small extent in control cells that were not treated with isotope tracers. Although a clear difference between untreated and tracer-treated samples was observed (**new Supplementary Fig. 1d**), all data were corrected for natural isotope contributions as follows:
The expected abundance of naturally occurring isotopes was calculated from the molecular formula of each analyzed lipid species using the Agilent MassHunter Isotope Distribution Calculator (Version 10.0.10305.0). For example, for PG 16:0-18:1, the calculated contribution to the m/z +3 signal is 0.0228, indicating that 2.28% of the unlabeled lipid is inherently detected in the m/z +3 channel. To correct for this contribution, the corresponding m/z +0 signal of, in this case, PG 16:0/18:1 was multiplied by this factor, and the resulting value was subtracted from the measured m/z +3 signal. After correction, the m/z +3 signal in control cells was typically reduced to values at or near zero, whereas tracer-treated cells retained clear signals.
In rare cases, the corrected m/z +3 signal in control cells remained slightly above zero. To account for these instances, this residual value was additionally subtracted from the isotope-corrected signal of the tracer-treated cells to ensure accurate quantification of tracer incorporation. We have now described this correction more clearly in the revised materials and methods section (**Methods: page 11**).

We hope that the reviewer now agrees that chromatographic separation, targeted MRM transitions, and isotope correction ensure the right identification and quantitation of labeled BMP & PG species and prevent mis-quantification due to isotopic overlap.

We have included **new Supplementary fig. 1** as validation of our LC-MS based methods and referenced it in the main text as follows:

Results, page 3, paragraph 2: ….. In comparison, no substantial labeling was detected for other polyglycerophospholipids, such as PG and cardiolipin (CL), as well as other GPL classes (Fig. 1d). The applied LC-MS method enabled baseline separation of the structural isomers BMP and PG. (Supplementary fig. 1a-c).
Results, page 3, paragraph 3:…. For polyglycerophospholipids, we also detected species containing more than one labeled glycerol *(m/z* shift of +6 and +9), suggesting that the ^{13}C-labeled glycerol was used for PA and head group synthesis. Representative chromatograms of the two most abundant BMP and PG species confirmed the selective incorporation of [^{13}C]GPG^{+3} into BMP and the preferential use of [U-^{13}C]glycerol for PG synthesis (**Supplementary fig. 1d**). ….

Minor Points:
1. Please elaborate on the metabolic source of GPG. Additionally, provide some discussion on the transport mechanism of ER-synthesized LPG to the lysosome for BMP synthesis.

Answer: We have now discussed that PG and BMP are metabolic sources of GPG with different stereoconfiguration (page 15, paragraph 3, and page 16, paragraph 1). The transport mechanism of LPG is currently unknown and discussed on page 15, paragraph 2.

2. Fig. 2j,k: Please clarify the experimental setup (e.g., were purified enzymes utilized?). The LPG signal appears low relative to background noise; representative ion chromatograms (EICs) should be provided, accompanied by a detailed description of the peak annotation logic.

Answer: We have now clarified in the main text that we used purified CLN8. The ion chromatograms of the analyzed LPG fragments are shown in **new Supplementary fig. 2**. A detailed description of the peak annotation is provided in the corresponding figure legend.

3. In the relevant figures, please indicate the stereochemical configuration (R or S) for each chiral carbon.

Answer: As suggested, the stereochemical configuration of GPG is now illustrated in **Fig 1** and **Fig 5.**

4. Fig. 1m,n: Triacsin C treatment reduces the incorporation of newly synthesized [13C]-LPG and [13C]-BMP, yet while total BMP levels decrease, total LPG levels remain largely unchanged. Please explain.

Answer: The incorporation of ^{13}C-GPG reflects de novo synthesis of LPG and BMP, which is reduced in the presence of Triacsin C (Fig. 1 i,j). Our observations suggest that cells contain two distinct pools of LPG. One pool consists of *S*-acylated LPG produced by CLN8, whereas the other comprises *R*-acylated LPG species that can be generated through PG hydrolysis. The applied LC–MS–based analysis does not distinguish between these two LPG pools. The levels of *R*- and *S*-acylated LPG may change independently and potential alterations in *S*-acylated LPG may be masked by opposing changes in unlabeled *R*-acylated LPG. Accordingly, the reduction in de novo synthesis may, but does not necessarily, lead to changes in lipid concentrations (labeled plus unlabeled, Fig. 1m,n). Furthermore, reduced conversion of LPG into PG and reduced GPG acylation may lead to unchanged LPG concentration.

5. Cardiolipin is not usually quantitatively measured using tri-quad mass spectrometers. Please provide evidence supporting the method validity.

Answer: Cardiolipin species were monitored on the triple quadrupole instrument using an alternative tandem mass spectrometry (MS/MS) acquisition mode referred to as "pseudo-MRM", in which the same precursor and product ion (Q1 = Q3) are monitored while applying low collision energy ≈10 V. This approach enables sensitive detection of intact cardiolipin ions. The cardiolipin calibration curve (using tetraoleoyl cardiolipin 72:4 as standard) showed a clear linear response across the tested concentration range, with excellent linearity ($R^2 = 0.996$). This confirms that cardiolipin can be accurately quantified using these acquisition conditions (see Figure R2 below).

This pseudo-MRM acquisition mode has already been described for other analyte classes. In particular, pseudo-MRM transitions have been successfully applied on triple quadrupole instruments for the quantification of small molecules (Galmiche M. et al., Rapid Commun Mass Spectrom. 2022 Jul 15;36(13):e9307).

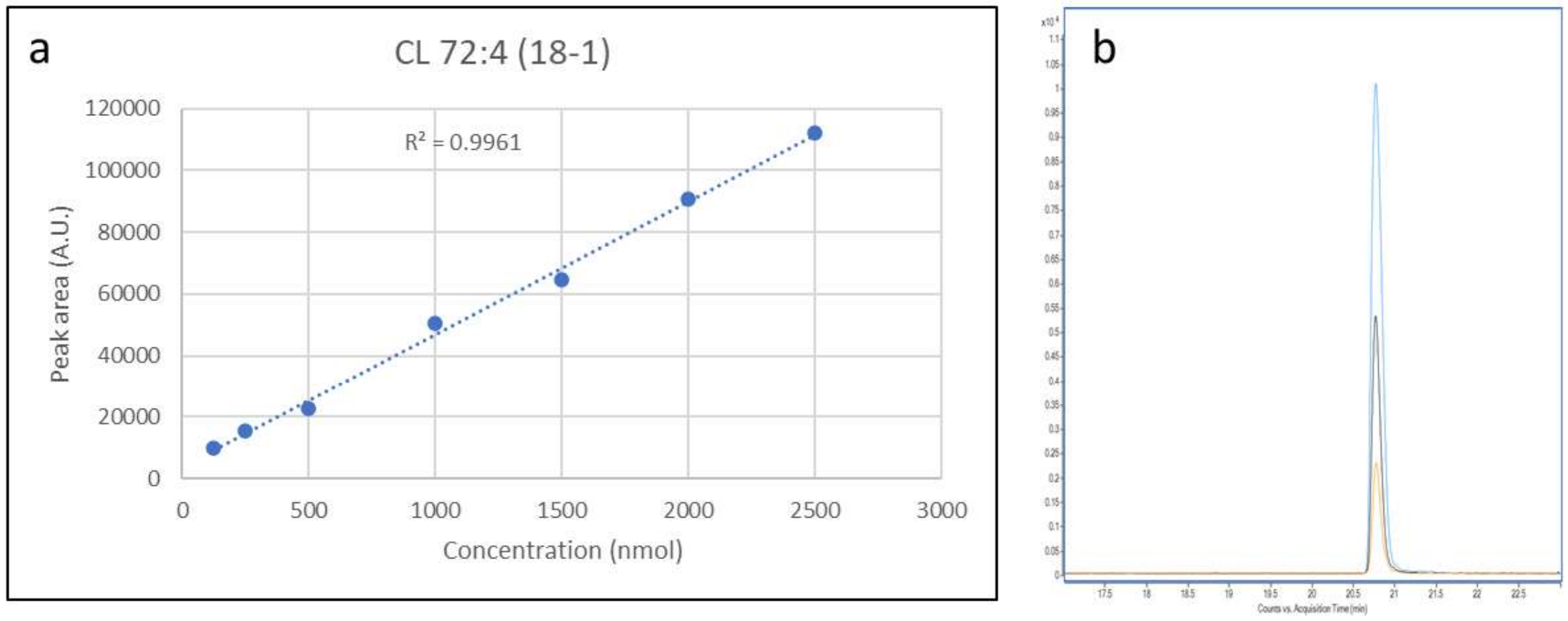

Figure R2: Validation for MS based cardiolipin analysis. **a**, Calibration curve of cardiolipin CL 72:4 measured using pseudo-MRM on a triple quadrupole MS. **b**) Representative chromatograms of cardiolipin CL 72:4 standard dilutions.

6. Please correct typographical errors in the supplementary materials; for example, Supplementary Table 4 is currently listed as "Neagtive."

<u>Answer:</u> Done.

Reviewer #1 (Remarks on Protocol(s)):

Please refer to my related comments in the general comments section.

<u>Reviewer #2 (Remarks to the Author):</u>

Breithofer et al. have conducted a thorough study of the biosynthesis of BMP and the involvement of CLN8 therein (and, to a lesser extent, the involvement of CLN5). The authors examined these processes in a range of preparations, including “lysates” (1000 x g supernatants of homogenized (cultured) cells and mouse tissues), sonicated membranes of cultured cells, live cells (unmodified, transfected, KO), live mice, as well as purified CLN8. This study also included a significant organic synthesis effort. Altogether, the authors provide convincing data for CLN8 catalyzing an essential step in the synthesis of BMP, acylating GPG in the ER, producing LPG that is transported to lysosomes, where it is converted to BMP by CLN5. CLN8 selectively acylates the S-linked glycerol moiety of GPG, and is essential for the synthesis of endogenous 2,2’-BMP. In addition, the authors show that BMP supplementation rescues the phospholipidosis of CLN8-deficient cells; the exogenous sn-3,3'-S,S-BMP is in part converted to sn-2,2'-S,S-BMP, albeit less efficiently in CLN8-KO cells than in WT cells.

Comments

[1] In the Main section (p.2), the first paragraph mentions “luminal membranes” twice. The term “intralumenal vesicles” better reflects the structure of these entities.

<u>Answer:</u> “Lumenal” was changed to “intralumenal”.

[2] In the first paragraph of the Results section (p.3), the second sentence talks about GPD

degradation, which is described as something that is "currently thought". However, the review that the authors refer to here is from 2014. An actual recent review on this topic is that of Hejazian et al. (https://doi.org/10.1007/s10930-024-10190-4).

Answer: We now cite the review by Hejazian et al. (2024).

[3] In multiple figures (e.g. Fig 1, Fig 2 j, Extended data Fig. 3, Extended data Fig. 7) some of the lettering is so small that it will be challenging for readers to decipher without considerably zooming in. Even if the journal allows this, the very small fonts do the readers a disservice. The challenge for the authors is to present their data in an accessible graphic fashion – without having to resort to microscopic lettering.

Answer: Letter size in figures was increased.

[4A] On p. 10, the third paragraph poorly connects with the preceding paragraphs. It is preferable to change the order of this sentence, for example "In the rescue experiments we added sn-3,3'-S,S-BMP to CLN8-KO cells, which prevented phospholipidosis (Fig. 3g-j). It is remarkable that 3,3'-BMP rescued the pathology of CLN8-KO cells, considering that 2,2'-BMP is the native form of BMP. Recent studies have shown …".

Answer: We have now changed paragraph 3 according to the reviewer's comment (see point 4B).

[4B] This paragraph continues with comparing the levels of the different forms of BMP in BMP-treated WT and CLN8-KO cells, stating that the treated KO cells had 60% less 2,2'-BMP than treated WT cells. The paragraph concludes by stating that supplementing the KO cells with 3,3'-BMP – "nevertheless" – rescued them from phospholipidosis, as if this is something not fully understood. Curiously, the authors do not point out that the 3,3'-BMP-treated KO cells had a lot more 2,2'-BMP relative to untreated KO cells, as they had no 2,2'-BMP at all (Fig. 3 l and m). In fact, the level of 2,2'-BMP in sn-3,3'-S,S-BMP-supplemented KO cells was higher than that of untreated WT cells (Fig. 3 l and m). Is it not plausible that this physiological level of 2,2'-BMP in the KO cells was sufficient to stave off phospholipidosis?

Answer: We have modified the main text and point out that sn-2,2 BMP is 2.8-fold above basal WT levels, which is sufficient to rescue the phospholipidosis phenotype.

Results, page 10, paragraph 3: In rescue experiments, the addition of *sn*-3,3'-*S,S*-BMP prevented phospholipidosis in CLN8-KO cells (**Fig. 3g,h**), although *sn*-2,2'-*S,S* BMP is the native form of BMP[29–31]. Recent studies demonstrated that the 2,2' acyl-configuration of *S,S*-BMP determines its resistance to lysosomal hydrolysis[2]. To compare *sn*-3,3'-*S,S*-BMP uptake and remodeling in WT and *CLN8*-KO cells, we analyzed the cellular BMP content, FA composition, and positional isomers. The addition of BMP strongly increased cellular 18:1-18:1 BMP levels in both genotypes (**Fig. 3k**). BMP subspecies containing other FAs than 18:1 (18:0, 18:2, and 22:6) increased only in WT cells (**Fig. 3k**). Analysis of the positional isomers, which separate under the applied chromatography (**Supplementary fig. 1a**), revealed that the 3,3' and 2,3' BMP were increased 1.7 and 2.6-fold in *CLN8*-KO cells, respectively, compared to WT cells, while 2,2'-BMP levels were reduced by 60% (**Fig. 3l,m**). These observations suggest that CLN8 affects FA composition and positional remodeling of BMP. Nevertheless, *sn*-2,2'-BMP isomer levels in supplemented *CLN8*-KO cells increased 2.8-fold above basal WT levels, which was sufficient to rescue the phospholipidosis phenotype.

[5] In the last paragraph of p. 10, results are presented of supplementing WT and CLN8-KO cells with commercially available R, rac LPG. In the treated KO cells, neither 2,2'-BMP nor 3,3'-BMP were detected. By contrast, the R, rac LPG supplementation restored 2,3'-BMP in KO cells to a

similar level as that of untreated WT cells (Extended data Fig. 5). Different from the BMP supplementation experiments discussed ahead of this paragraph, no information is provided on the phenotype of the R, rac LPG-supplemented CLN8-KO cells. Did this treatment reduce the extent of phospholipidosis? If not, it is more plausible that the restoration of 2,2'-BMP by supplementation with 3,3'-BMP (Fig. 3 l and m) rescued the KO cells from phospholipidosis.

Answer: To address this critical comments, we have repeated the experiments and compared the supplementation of *sn*-1-oleoyl-*R*,rac LPG and *sn*-2,2'-18:1-18:1-*S,S* BMP under identical conditions. In contrast to *sn*-3,3'-*S,S* BMP, *sn*-1-*R*,rac LPG supplementation did not rescue phospholipidosis in CLN8-KO cells (**Extended data fig. 5c,d**).

[6] Further to these supplementation experiments (p. 10), it is curious that the WT and CLN8-KO cells were cultured in the presence of 3,3'-BMP for 72-96 hours (Fig. 3 h and k), while the cells were cultured in the presence of R, rac LPG for only 18 hours (Extended data Fig. 5). This of course makes one wonder what the outcome of a 96-hour supplementation with R, rac LPG could be. What made was the reason for limiting the R, rac LPG supplementation to 18 hours?

Answer: To improve the comparability between BMP and LPG supplementation, we have now repeated experiments (lipidomics and microscopy) under identical conditions [Incubation time (72h) and same LPG and BMP concentrations (5 µM)] (**Fig. 3k-m**).

[7] The last sentence on p. 10 does not require "respectively". How about rewriting part of this sentence as follows: "Notably, exogenous LPG raised the GPG content of WT and of CLN8-KO cells 80-fold, suggesting that …".

Answer: The proposed changes have been adopted.

[8] Page 12, the first paragraph of the "CLN8 acylates GPG in vitro and in vivo" section. This paragraph deals with loading live cells with [13C]GPG. For continuity of the paper, it may be good to remind the reader here of the findings of Figs. 1 d and h, where similar experiments were done, albeit with only WT HEK293 cells.

Answer: We have adapted the paragraph accordingly:

Results, page 12, paragraph 1: Incubation of WT cells with [^{13}C]GPG^{+3} resulted in the formation of isotope-labeled LPG, BMP and hemi-BMP (**Fig 1d,h**). Accordingly, we next tracked the incorporation of [^{13}C]GPG^{+3} into lipids of *CLN8*-KO cells. In contrast to WT controls, mutant Hek293 and Hap1 cells were unable to produce [^{13}C]BMP^{+3} (**Fig. 4a, Extended data fig. 6a**) and showed a ~ 95% reduction in [^{13}C]LPG^{+3} formation (**Fig. 4b, Extended data fig. 6b**). Thus, CLN8 is rate-limiting for the incorporation of GPG into phospholipids.

[9] Page 12, the second paragraph of the "CLN8 acylates GPG in vitro and in vivo" section. Here, an earlier experiment with BafA1 (Fig. 1 p) is revisited, in one brief sentence, which is somewhat jarring. It would be good to describe the earlier experiments more extensively, to bring the reader up to speed without having to interrupt their reading of this section by going back to p. 4. Only after a more extensive reminder, the new experiments with BafA1 can be described (Fig. 4 d).

Answer: We have adjusted the paragraph accordingly:

Results, page 12, paragraph 2: Blocking lysosomal acidification with BafA1 led to the extra-lysosomal accumulation of LPG and decreased BMP levels (**Fig. 1o,p**). *CLN8*-deficiency completely prevented LPG

accumulation in BafA1-treated Hek293 and Hap1 cells, confirming that CLN8 mediates acylation of GPG at the ER (**Fig. 4d, Extended data fig. 6e**).

[10] In the final paragraph of the Discussion (p. 15), the authors briefly discuss the study of Singh et al. (https://doi.org/10.1016/j.cell.2024.09.036, reference 10). This study was reviewed by experts in BMP metabolism, in the Annual Review of Biochemistry (https://doi.org/10.1146/annurev-biochem-092823-113814). Singh et al. proposed a distinct metabolic pathway for BMP synthesis, requiring lysosomal phospholipases D3/D4 (PLD3/4). The authors of the current manuscript need to significantly expand their discussion of the paper by Singh et al., comparing and contrasting it with their own work. It is important that the authors point out in some detail how the approaches of Singh et al. led to insights that are fundamentally different from those of the current manuscript. Alternatively, the authors could provide a synthesis of both studies, showing how the roles of PLD3/4 and CLN8 may be complementary, following the wider scope of the Discussion of Bulfon et al. (https://doi.org/10.1038/s41467-024-54213-1). With the current knowledge, would it be is possible to arrive at a unified vision of BMP synthesis, or are there outstanding issues to be resolved experimentally?

Answer: We hank the reviewer for this thoughtful suggestion. We have extended the discussion on the proposed mechanisms of *S,S*-BMP synthesis (**page 15-16, paragraph 3&4, Fig. 5**). Our tracer studies and experiments of Thornburg et al. suggest that BMP synthesis can proceed without exchange of the backbone glycerol, indicating an intramolecular stereoconversion mechanism (Fig. 5c). In contrast, Singh et al. proposed a transphosphatidylation reaction that mediates the stereoconversion (**Fig. 5d**). This reaction is catalyzed by PLD3/4 and leads to the loss of the *R*-linked glycerol.

It is important to note that our observations do not exclude the PLD3/4-catalyzed reaction. Our experiments were performed with a racemic [^{13}C]GPG^{+5} mixture containing *S,S*-GPG, which can be utilized for the synthesis of *S,S*-BMP without the need for stereoconversion (Fig. 5b). Thus, both pathways of *S,S*-BMP synthesis may exist in parallel. CLN8 actually produces the LPG intermediate (*R,S*-acyl-LPG), which acts as a phosphatidyl donor in the reaction proposed by Singh et al.

We highlight the following outstanding questions that need to be clarified, particularly with regard to the mechanism of stereoconversion: (i) What is the mechanism of intramolecular stereoconversion? (ii) Do PLD3/4-catalyzed- and intramolecular stereoconversion pathways exist in parallel and does their relative contribution vary by cell type or physiological state? In order to fully elucidate the underlying molecular mechanisms of *S,S*-BMP synthesis in neuronal and peripheral cells, tracer studies using stereochemically pure *R,S*- and *S,S*-GPG as well as analytical methods distinguishing between the stereoisomers of BMP and its precursors, are required (revised **Discussion, page16**).

Reviewer #2 (Remarks on Protocol(s)):

Methods have been adequately described.

Reviewer #3 (Remarks to the Author):

In this manuscript, the authors identify CLN8 as a glycerophosphoglycerol (GPG) acyltransferase that initiates a previously unrecognized, non-canonical pathway for bis(monoacylglycero)phosphate (BMP) synthesis. Through a combination of isotope tracing, biochemical reconstitution, genetic loss-of-function models, and in vivo mouse studies, the authors demonstrate that CLN8 catalyzes the stereospecific acylation of GPG to generate lysophosphatidylglycerol, which is selectively routed toward BMP synthesis in lysosomes. Loss of

CLN8 results in BMP deficiency, lysosomal phospholipid accumulation, and phospholipidosis that can be partially rescued by BMP supplementation.

Overall, this study provides a compelling biochemical explanation linking CLN8 function to lysosomal lipid homeostasis and offers a mechanistic framework for understanding lysosomal storage defects in CLN8-associated Batten disease. Conceptually, the work adds to canonical models of phospholipid biosynthesis by demonstrating that a glycerophosphodiester, traditionally viewed as a degradation intermediate, can be directly reutilized for lipid synthesis in mammalian cells.

Comments/Suggestions:
1. While the study convincingly demonstrates that mammalian cells can reutilize GPG for BMP synthesis, the manuscript provides limited insight into how frequently or under what physiological conditions this pathway contributes meaningfully to BMP pools. GPG is generally considered a transient degradation intermediate rapidly cleared by glycerophosphodiesterases. The elevated GPG levels observed in CLN8-deficient cells may reflect pathological accumulation rather than normal metabolic flux. Thus, it remains unclear whether the CLN8-dependent pathway is a dominant contributor to BMP synthesis under basal conditions or becomes particularly important during periods of elevated membrane turnover, lysosomal stress, or disease.

Answer: We thank the reviewer for this critical comment and for prompting us to clarify the physiological context and relevance of GPG dependent BMP synthesis. We agree it is important to distinguish between what our data directly demonstrate and what remains to be established about pathway usage across conditions.

Regarding GPG stability, we did not find evidence that GPG has a short half-life in mammalian cells. Consistent with this, GPG accumulates in lysosomes of CLN3-deficient cells (PMID: 36131016), suggesting limited or absent degradation in lysosomes. To test GPG stability in serum, we incubated 1 mM [13C]GPG^{+5} with 10% human and bovine serum for 18h at 37°C and observed that only 9% and 1.5% of GPG-bound glycerol moieties were released, respectively (**Fig. R3**). This observation indicates high stability of GPG in the circulation and serum containing cell culture media. In cells, backbone glycerol from GPG is incorporated into LPG, hemi-BMP, and BMP, with minimal diversion of GPG-derived glycerol into other lipids (**Fig. 1d,h**). Overall, our data indicate that GPG is not rapidly degraded, enabling its incorporation into BMP.

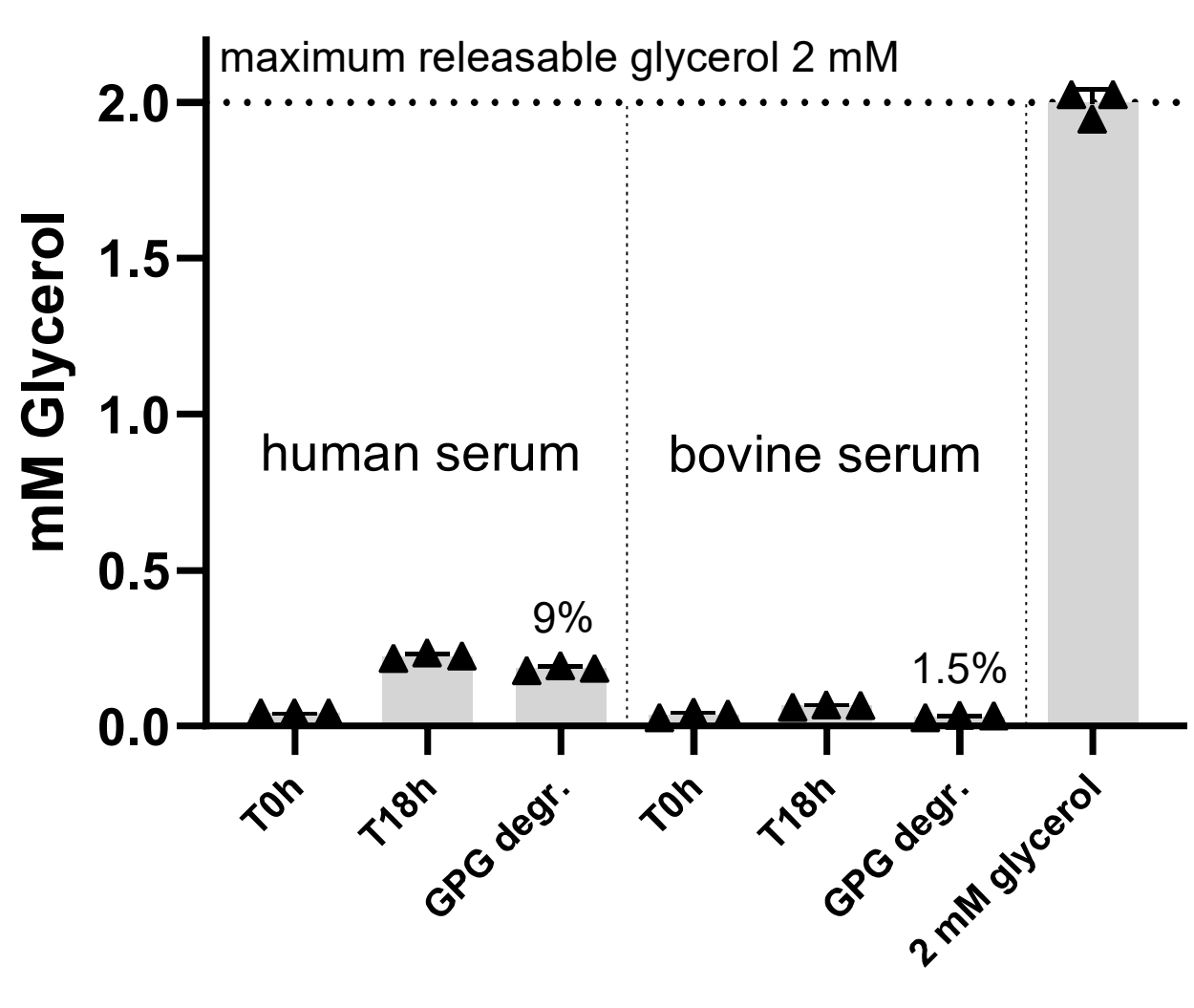

Figure R3. Stability of GPG in human and bovine serum. [13C]GPG^{+5} (1 mM) was incubated in 10% human or bovine serum diluted in PBS for 18h at 37°C. Glycerol release was quantified using a colorimetric assay (Free Glycerol Reagent, Sigma F6428) to assess GPG degradation mediated by glycerophosphodiesterase activity. Glycerol concentrations were determined at the start of the incubation period (T0) and after 18h. To calculate GPG degradation, T0 values were subtracted from T18 values. Serum incubated in the absence of GPG was used as background control.

The reviewer raised an important point regarding the conditions under which the GPG-dependent BMP synthesis pathway contributes to BMP pools. The observation that CLN8-deficient cells and mouse tissues completely lack BMP suggests that the CLN8-catalyzed reaction is essential for BMP synthesis under basal conditions. We agree that the accumulation of GPG in CLN8-deficient cells likely reflects a pathological accumulation, as the lack of CLN8 activity leads to accumulation of GPG acting as acyl acceptor in the CLN8-catalyzed reaction. Our data suggest that CLN8 is rate-limiting for the incorporation of GPG into phospholipids.

Lysosomal lipid storage disorders are frequently associated with BMP accumulation (PMID: 17805872; PMID: 18052935). CLN8 deficient cells also develop a lysosomal lipid storage disorder. Even under these conditions cells are not able to produce BMP, suggesting that BMP synthesis requires CLN8 also under pathological conditions.

2. A significant portion of the study relies on isotope-tracing experiments using relatively high concentrations of exogenous GPG (typically 250–500 µM in cells and ~55 mg/kg in vivo). While these concentrations facilitate robust detection of labeled lipids, they likely exceed physiological intracellular GPG levels. At such concentrations, tracer-driven effects may bias pathway usage, potentially exaggerating the apparent importance or selectivity of CLN8-mediated GPG acylation.

Answer: We agree that the concentrations used in our cell-based and in vivo tracer studies likely exceed physiological GPG levels. The GPG doses were chosen to ensure robust detection of labeled lipids. In cell experiments, ^{13}C-labeled LPG and -BMP were already detected at 50 µM GPG (lower concentrations were not tested), and labeling efficiency increased with higher concentrations (**Fig. R4**). These studies were done to confirm that CLN8 is essential for GPG acylation. It is important to note that, even at supraphysiological GPG concentrations, CLN8-KO cells cannot incorporate GPG into phospholipids. Furthermore, it must be considered that we can observe changes in GPG, LPG, and BMP in CLN8-KO cells in the absence of the tracer (**Fig. 3b-e**), where GPG is provided from endogenous sources.

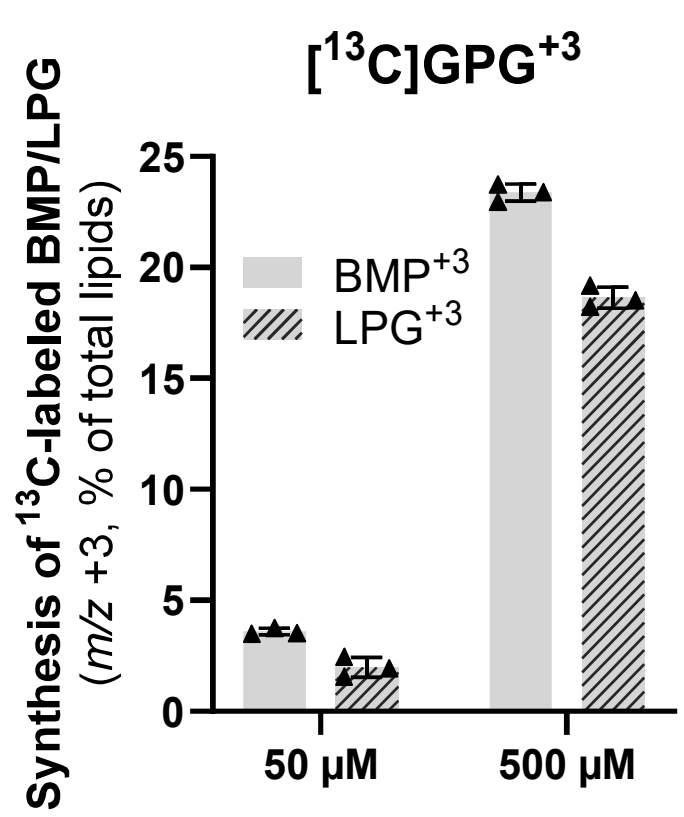

Figure R4: Dose-dependent incorporation of [^{13}C]GPG^{+3} (50 and 500 µM) into BMP and LPG of HEK293 cells. Incubations were performed for 18h under standard cell culture conditions. The results

are presented as a percentage of labeled lipids relative to total lipids for each lipid class examined.

3. The GPG tracers used are racemic mixtures. While the authors address stereochemical specificity mechanistically, the endogenous stereochemical composition of cellular GPG pools is not established. As a result, extrapolation from tracer behavior to native metabolism remains somewhat uncertain, particularly when drawing conclusions about stereoselective routing under physiological conditions. This limitation should be discussed more explicitly.

Answer: We agree with the reviewer that this is a limitation of our study. We do not currently have the means to separate stereoisomers of GPG and BMP, which would require derivatization and chiral columns. Consequently, our tracer data cannot define the endogenous stereochemical composition of cellular GPG pools or definitively assign stereoselective routing in vivo. To address this point, we have modified the discussion and indicate the limitations of the study:

Discussion, page 16, paragraph 1: In order to fully elucidate the underlying molecular mechanisms of *S,S*-BMP synthesis in neuronal and peripheral cells, tracer studies using stereochemically pure *R,S*- and *S,S*-GPG, as well as analytical methods distinguishing between the stereoisomers of BMP and its precursors, are required.

4. Most tracer experiments are conducted over 16/18-hour incubations, a duration that allows for secondary lipid remodeling, recycling, and compensatory responses. These long incubations reduce temporal resolution and make it difficult to distinguish primary, direct metabolic events from downstream consequences of prolonged tracer exposure.

Answer: We used the 16-18h incubation periods primarily to improve labeling efficiency and detection robustness. Importantly, labeled LPG and BMP were already clearly detectable after 4h (**Fig R5**). Our double-labeled tracers allow us to distinguish direct GPG acylation from incorporation of degradation products, independent of the incubation period. The formation of m/z +5 labeled BMP in the presence of [^{13}C]GPG^{+5} confirms that we monitor the direct acylation of GPG. Degradation of [^{13}C]GPG^{+5}and the reuse of ^{13}C-labeled glycerol for lipid synthesis leads to the formation of lipids with m/z +3 or m/z +2.

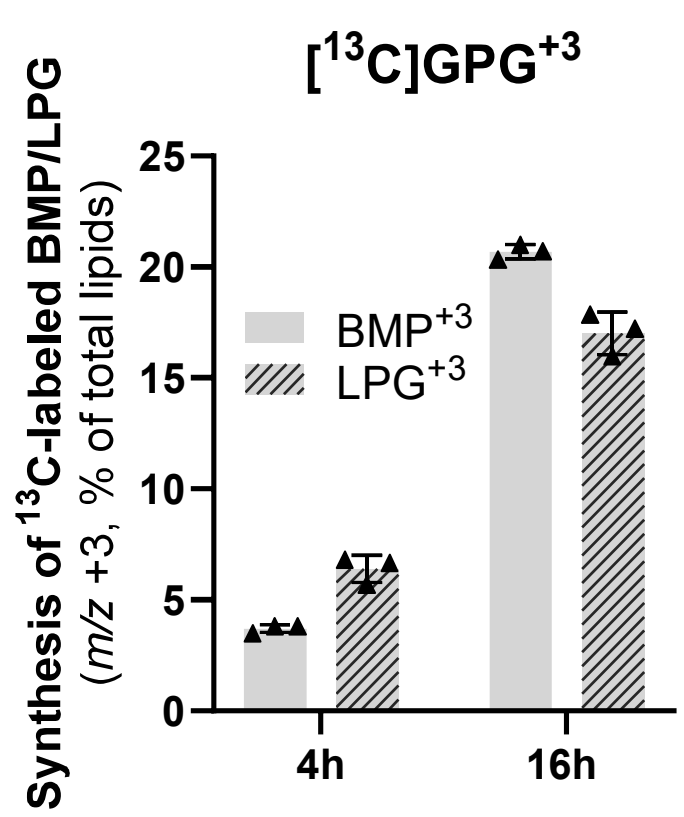

Figure R5: Time dependent incorporation of [^{13}C]GPG^{+3} (500 µM) into BMP and LPG of Hek293 cells. Incubations were performed for 4h or 16h under standard cell culture conditions. The results are presented as a percentage of labeled lipids relative to total lipids for each lipid class examined.

5. The proposed model requires selective delivery of CLN8-generated LPG from the ER to lysosomes, yet no direct trafficking mechanism is demonstrated. The absence of identifiable lipid transfer proteins, vesicular intermediates, or ER–lysosome contact site involvement leaves an important gap in the mechanistic narrative. While this may be beyond the scope of the current study, more explicit acknowledgment of this unresolved step would strengthen the overall model.

Answer: We thank the reviewer for this important comment, we have modified the discussion accordingly**:**

Discussion, page 15, paragraph 2: Although this study did not address the transport of LPG between the ER and lysosomes, our findings suggest the existence of mechanisms involving either vesicular or non-vesicular transport. Previous work demonstrated that CLN8 is an ER-associated transmembrane protein that regulates lysosomal biogenesis by promoting the ER-to-Golgi transfer of lysosomal enzymes produced at the ER. This observations indicates an additional function of CLN8 that is independent of its enzymatic activity[40,41]. Currently, it is unknown whether the total loss of CLN8 protein and the expression of inactive variants produce different phenotypes. However, inactive CLN8 variants with a point mutation in the active center cause Batten disease[15,16,22], suggesting that the enzymatic activity is essential for lysosomal homeostasis. We show that overexpression of the inactive Batten disease–associated CLN8 variant R204C cannot rescue BMP-deficiency.

6. The lipid species proposed in Figure 1 and 4 appear to be assigned primarily based on m/z shifts following isotope labeling. While this approach is informative for tracking incorporation, it is insufficient on its own for unambiguous lipid identification. MS/MS-based structural validation is necessary to confirm lipid class and acyl-chain positioning. It is possible that such analyses were performed, but this is not clearly described or presented in the manuscript and should be clarified.

Answer: A detailed characterization and validation of our MS-based method to quantify BMP and PG isomers is now shown in new **Supplementary fig. 1** (see also major point 1 and 2 of reviewer 1). We have also included the validation for the separation of BMP positional isomers using *sn*-2,2'-S,S-18:1-18:1 BMP and *sn*-3,3'-S,S-18:1-18:1 BMP standards.

7. The analysis in Figure 2 relies on differential cell lysate fractions that are presumed to be enriched for distinct subcellular compartments. However, comprehensive characterization of these fractions is not shown. Inclusion of marker-based validation would be important to confirm that the fractions correspond to the intended subcellular structures and to support the conclusions drawn from these experiments.

Answer: According to the reviewer's criticism, **Extended data fig. 1a** now includes a Western blot showing marker proteins of the analyzed subcellular fractions. These include succinate-dehydrogenase (SDHA) as a marker for mitochondria, calnexin as a marker for microsomes, and glycerinaldehyd-3-phosphate (GAPDH) as a cytosolic marker.

8. Clarification needed regarding BMP supplementation experiments: The rescue experiments utilize a non-endogenous BMP species. Although this distinction is noted, it remains unclear how much of the observed positional isomer distribution reflects physiological BMP remodeling versus cellular handling of an exogenously supplied lipid.

Answer: Previous data already suggested that *sn*-3,3'-BMP stereoisomers (*S,S* / *R,R* / *S,S*) improve the phenotype of NPC1-deficient cells (PMID: 39395801), suggesting that they are converted into the major hydrolysis-resistant *sn*-2,2' isomers. Accordingly, the use of sn-3,3'-BMP isomers offers a possibility to monitor the positional remodeling of BMP in cell experiments. We found that sn-3,3'-

S,S-BMP supplementation rescues the phospholipidosis phenotype of CLN8-deficient cells (**Fig. 3g,j**) and also tested whether CLN8 affects the positional remodeling of BMP (**Fig 3k-m**).

We have now clarified this point in the main text:

Results, page 10, paragraph 3: In rescue experiments, the addition of *sn*-3,3'-*S,S*-BMP prevented phospholipidosis in CLN8-KO cells (**Fig. 3g,h**), although *sn*-2,2'-*S,S* BMP is the native form of BMP[29–31]. Recent studies demonstrated that the 2,2' acyl-configuration of *S,S*-BMP determines its resistance to lysosomal hydrolysis[2]. To compare *sn*-3,3'-*S,S*-BMP uptake and remodeling in WT and *CLN8*-KO cells, we analyzed the cellular BMP content, FA composition, and positional isomers. The addition of BMP strongly increased cellular 18:1-18:1 BMP levels in both genotypes (**Fig. 3k**). BMP subspecies containing other FAs than 18:1 (18:0, 18:2, and 22:6) increased only in WT cells (**Fig. 3k**). Analysis of the positional isomers, which separate under the applied chromatography (**Supplementary fig. 1a**), revealed that the 3,3' and 2,3' BMP were increased 1.7 and 2.6-fold in *CLN8*-KO cells, respectively, compared to WT cells, while 2,2'-BMP levels were reduced by 60% (**Fig. 3l,m**). These observations suggest that CLN8 affects FA composition and positional remodeling of BMP. Nevertheless, *sn*-2,2'-BMP isomer levels in supplemented *CLN8*-KO cells increased 2.8-fold above basal WT levels, which was sufficient to rescue the phospholipidosis phenotype.

9. The in vivo tracer experiments fail to demonstrate BMP synthesis in brain tissue despite the neurological focus of the disease. Without further discussion, it remains unclear whether this reflects technical limitations of tracer delivery or fundamental differences in BMP metabolism in the brain.

<u>Answer:</u> We agree that the lack of brain labeling in vivo requires clearer explanations, especially given the neurological focus of the disease. In vivo tracer studies revealed that GPG is incorporated into BMP of peripheral tissues of wild-type, but not of CLN8-deficient mice. Indeed, we could not monitor the incorporation of the GPG tracer into brain lipids, because GPG does not cross the blood–brain barrier. Nevertheless, our findings strongly support a critical role for CLN8 in BMP synthesis in neuronal cells: (i) brain tissue exhibits the highest GPGAT activity (**Fig. 1c**); (ii) GPGAT activity is undetectable in the brain tissue of CLN8-deficient mice (**Fig. 4e**); and (iii) the brains of CLN8-deficient mice show depleted BMP levels (**Fig. 4f**). We conclude that there are no fundamental differences in BMP synthesis between the brain and peripheral tissues. However, our conclusions regarding the mechanisms that may drive BMP stereoconversion are based on the observation that the GPG tracer is incorporated without exchange of glycerol moieties in peripheral tissues. Because we did not detect labeling of brain BMP, our insights into mechanisms underlying stereoconversion in neuronal cells are limited. This limitation is now addressed in the discussion:

Discussion, page 16, paragraph 1: However, it must be considered that *in vivo* experiments were performed with a racemic GPG mixture containing *S,S*-GPG, which can be utilized for the synthesis of *S,S*-BMP without stereoconversion (**Fig. 5b**). Furthermore, the GPG tracer was unable to cross the blood-brain barrier, preventing insights into mechanisms that promote the *R,S* to *S,S* conversion in neuronal cells. Thus it is possible that both intramolecular stereoconversion and transphosphatidylation mechanisms contribute to *S,S*-BMP synthesis. In order to fully elucidate the underlying molecular mechanisms of *S,S*-BMP synthesis in neuronal and peripheral cells, tracer studies using stereochemically pure *R,S*- and *S,S*-GPG, as well as analytical methods distinguishing between the stereoisomers of BMP and its precursors, are required.

10. Most mechanistic conclusions are derived from HEK293 and Hap1 cells, which may not fully capture the lipid metabolic context relevant to neurodegeneration. Given the otherwise high level

of rigor and depth in the study, the limited use of more disease-relevant cellular models stands out and warrants discussion.

Answer: We agree that HEK293 and HAP1 cells do not fully recapitulate the lipid metabolic context of neurodegeneration and that this limitation should be stated clearly. We used these cells because they express the key components of the BMP synthesis pathway and have been used in recent studies to investigate BMP metabolism (Medoh et. al., Science, 2024; PMID: 37708259). Crucially, our key findings were confirmed in the *Cln8*-deficient motor neuron degeneration mouse line, suggesting that the described mechanism is unlikely to be an artefact of HEK293/HAP1 biology. The consistency between *in vitro* and *in vivo* models underscores the general physiological relevance of our findings. Nevertheless, we agree that future studies on neuronal cell types, including microglia, astrocytes and neurons, will provide more detailed insights into BMP metabolism and may reveal cell-type-specific differences.

To address the reviewers concern, we have extended our discussion and indicate the limitations of the study:

Discussion, page 14, paragraph 2: Recent studies provided new insights into the pathogenic mechanisms of Batten disease, revealing that cells lacking CLN3, CLN5, CLN8, and CLN11 develop BMP-deficiency[9,22,36–38]. BMP acts as a cofactor of lysosomal lipid hydrolases and transport proteins that promote the breakdown and export of lipids[39]. It is therefore reasonable to assume that BMP-deficiency impairs lysosomal lipid catabolism in several genetically distinct forms of Batten disease. Our observations suggest that BMP-deficiency is causal for lysosomal lipid accumulation in *CLN8*-KO cells, as BMP supplementation rescued the phospholipidosis phenotype. Thus, the supplementation of BMP may represent a therapeutic option for *CLN8*-deficiency and other lysosomal storage disorders characterized by BMP-deficiency. Although the majority of mechanistic studies were conducted in Hek293 and Hap1 cells, studies in the *mnd* mouse model confirmed that *Cln8*-deficiency is associated with the lack of GPGAT activity and BMP in the brain and peripheral tissues. Further studies in Batten disease models are needed to evaluate the therapeutic potential of BMP supplementation.
